# The Hydrating Effects of Hypertonic, Isotonic and Hypotonic Sports Drinks and Waters on Central Hydration During Continuous Exercise: A Systematic Meta-Analysis and Perspective

**DOI:** 10.1007/s40279-021-01558-y

**Published:** 2021-10-30

**Authors:** David S. Rowlands, Brigitte Hani Kopetschny, Claire E. Badenhorst

**Affiliations:** School of Sport, Exercise and Nutrition, College of Health, Massey University, Albany Highway, Auckland, New Zealand

## Abstract

**Background:**

Body-fluid loss during prolonged continuous exercise can impair cardiovascular function, harming performance. Delta percent plasma volume (*d*PV) represents the change in central and circulatory body-water volume and therefore hydration during exercise; however, the effect of carbohydrate–electrolyte drinks and water on the *d*PV response is unclear.

**Objective:**

To determine by meta-analysis the effects of ingested hypertonic (> 300 mOsmol kg^−1^), isotonic (275–300 mOsmol kg^−1^) and hypotonic (< 275 mOsmol kg^−1^) drinks containing carbohydrate and electrolyte ([Na^+^] < 50 mmol L^−1^), and non-carbohydrate drinks/water (< 40 mOsmol kg^−1^) on *d*PV during continuous exercise.

**Methods:**

A systematic review produced 28 qualifying studies and 68 drink treatment effects. Random-effects meta-analyses with repeated measures provided estimates of effects and probability of superiority (*p*_+_) during 0–180 min of exercise, adjusted for drink osmolality, ingestion rate, metabolic rate and a weakly informative Bayesian prior.

**Results:**

Mean drink effects on *d*PV were: hypertonic − 7.4% [90% compatibility limits (CL) − 8.5, − 6.3], isotonic − 8.7% (90% CL − 10.1, − 7.4), hypotonic − 6.3% (90% CL − 7.4, − 5.3) and water − 7.5% (90% CL − 8.5, − 6.4). Posterior contrast estimates relative to the smallest important effect (*d*PV = 0.75%) were: hypertonic-isotonic 1.2% (90% CL − 0.1, 2.6; *p*_+_ = 0.74), hypotonic-isotonic 2.3% (90% CL 1.1, 3.5; *p*_+_ = 0.984), water-isotonic 1.3% (90% CL 0.0, 2.5; *p*_+_ = 0.76), hypotonic-hypertonic 1.1% (90% CL 0.1, 2.1; *p*_+_ = 0.71), hypertonic-water 0.1% (90% CL − 0.8, 1.0; *p*_+_ = 0.12) and hypotonic-water 1.1% (90% CL 0.1, 2.0; *p*_+_ = 0.72). Thus, hypotonic drinks were very likely superior to isotonic and likely superior to hypertonic and water. Metabolic rate, ingestion rate, carbohydrate characteristics and electrolyte concentration were generally substantial modifiers of *d*PV.

**Conclusion:**

Hypotonic carbohydrate–electrolyte drinks ingested continuously during exercise provide the greatest benefit to hydration.

**Graphical abstract:**

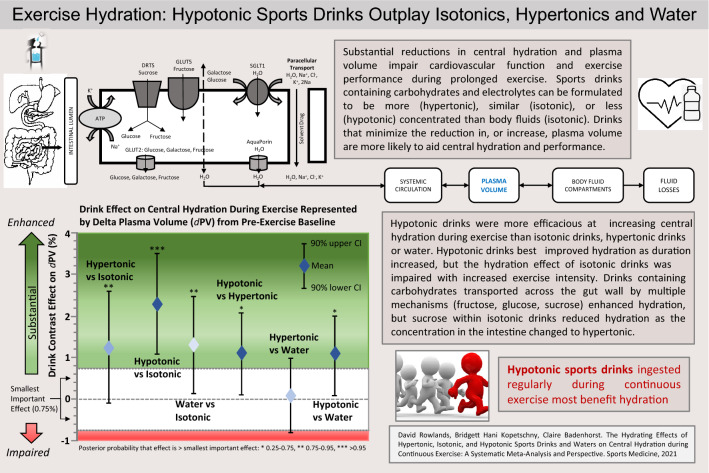

**Supplementary Information:**

The online version contains supplementary material available at 10.1007/s40279-021-01558-y.

## Key Points

**Table:** 

Some confusion exists around the effect of commonly ingested carbohydrate–electrolyte drinks and water on the hydration response during exercise.
We meta-analysed the common measure of central hydration status—delta plasma volume—and found that hypotonic carbohydrate–electrolyte drinks ingested continuously during exercise provide the greatest benefit to hydration when compared with hypertonic drinks, isotonic drinks and water.

## Introduction

Prolonged exercise leads to the loss of body fluid associated with elevated sweat rates [1–4]. Dehydration of greater than at least 3–4% of total body-water (> 2% body mass) can reduce cardiac output [5–9], increase perceived exertion [10], impair cutaneous and central thermoregulatory function [5, 8, 9, 11–13], and impair muscle blood flow [14] and endurance exercise performance in some [15–19] but not all conditions [20–22]. To offset the sometimes deleterious effects of dehydration on cardiovascular function and performance, drinks containing mostly carbohydrate and electrolytes are now widely recommended for ingestion to provide carbohydrate for energy [23, 24] and fluid to attenuate dehydration [25] and offset hyponatremia [23].

**Fig. 1:**
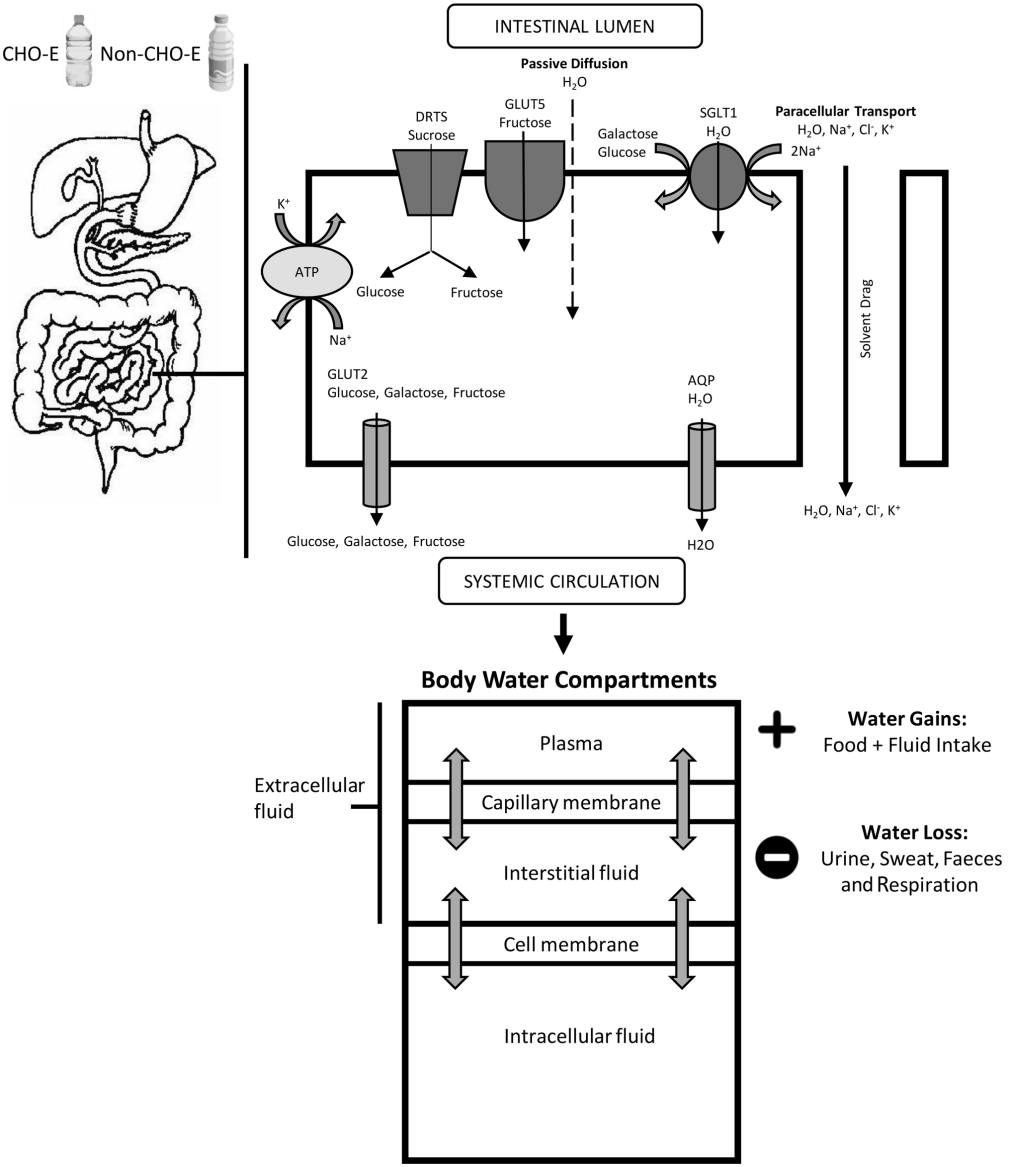
Summary of the basic physiology underlying the effect of carbohydrate–electrolyte drink (CHO-E) and non-carbohydrate–electrolyte drink (non-CHO-E) beverage ingestion on hydration. This figure summarises the processes believed responsible for the temporal relative expansion of body-water content following intestinal fluid absorption. Whole-body water exchange, gain, loss, distribution and osmotic equilibrium are indicated by the arrows across the plasma, interstitial and intracellular fluid compartments. Fluid transport across the gut epithelia occurs via passive and osmotic gradient and channel (aquaporin, AQP)-mediated processes, facilitated by carbohydrate transport and solvent drag [1, 24]. Fluid shifts across body fluid compartments occurs through a combination of rapid time-course hydrostatic pressure and osmotic pressure gradients, and slower time-course reabsorption in the kidneys [110]. *SGLT1* sodium-dependent glucose co-transporters, *GLUT5* fructose transporter, *DRTS* disaccharide-related transport system, *ATP* Na^+^/K^+^-ATPase

Because of the role of ingested carbohydrate (CHO)–electrolyte (CHO-E) (*sports drinks*) and non-carbohydrate–electrolyte (non-CHO-E) beverages (*sports waters*) on performance and health, there is considerable commercial and practical interest in the relative impact of sports drink composition on hydration during exercise, and in recent times with concern over sugar content, the emergence of less concentrated hypotonic carbohydrate–electrolyte drinks. The physiological rationale guiding the interest in (re)hydration with ingested CHO-E and non-CHO-E beverages during exercise lies in the rapid restoration or maintenance of body-fluid homeostasis, cardiovascular and thermoregulatory function [5, 9, 19, 26]. The question of what sports drink or sports water composition is better to hydrate/rehydrate during exercise depends upon the properties of the ingested drinks, which affect gastric emptying, intestinal fluid absorption, body fluid retention and (renal) excretion [1, 2, 27, 28] (Fig. 1). More comprehensive reviews on gastric emptying and intestinal absorption effects on fluid absorption are available [26, 29–31] and will not be reviewed here. However, a summary of these physiological processes is provided to assist readers in understanding the rationale for beverage formulation for the purposes of hydration during continuous exercise (Tables 1 and 2). By magnitude, the most influential factors are beverage volume and osmolality, the latter of which is determined primarily by CHO concentration and format, with sodium and other electrolytes having a lesser impact on absorption, plasma volume, and fluid retention during exercise [23, 32, 33].

**Table 1:** Carbohydrate–electrolyte beverage composition and ingestion characteristics known to influence gastric emptying (GE)

Factor	Effect
Volume	Direct proportional relationship between beverage volume and GE rate to volumes up to 600 mL, but nil association above [84], and an apparent upper threshold for GE at > 1000 mL [30, 84] High inter-individual variation [92, 93] Repeated solution ingestion to maintain high stomach volumes may aid in maintaining consistent GE rates [30]
Energy content	Glucose and total energy content have a greater inhibitory effect compared with beverage osmolality on GE [94] Inhibitory effect of 4–6% glucose solution (230–352 mOsm kg^−1^) vs. < 2% glucose and concentrations > 6% (> 350 mOsm kg^−1^) decrease GE rates [94] 8% glucose solution is emptied at a significantly slower rate when compared to 8% sucrose solution [94]
Carbohydrate type	Galactose empties faster than glucose and fructose empties faster than galactose [95] Starch empties at a similar rate to isocaloric glucose and maltodextrin and fructose empty faster than glucose [96, 97] Glucose and fructose at < 6% concentration are emptied faster than glucose, but glucose and fructose > 6% concentration are not different [98]
Osmolality	Type of carbohydrate affects osmolality and GE rates [31] Sucrose is less inhibitory than glucose at beverage osmolality 68–251 mOsm kg^−1^ [31] A glucose polymer will reduce the osmolality of the beverage and increase GE rate [99] Hyperosmolality reduces GE rates [99]
pH	Type and concentration of acids commonly used in beverages are not thought to influence GE. Stomach is acidic; beverage pH has minimal effect [29]
Temperature	Beverage temperature may affect GE, but effects are minor in size [29]
Sex	Females may have an initial faster GE rate than males due to smaller stomach generating higher intragastric pressure after the ingestion of large meals or beverage volumes [100]

**Table 2:** Carbohydrate-electrolyte beverage composition and factors known to influence intestinal absorption

Factor	Effect
Osmolality	Ingested beverages < 270 mOsmol L^−1^ may aid water absorption as a result of a favourable osmotic gradient encouraging water movement from the proximal small intestine across the mucosa [29] Electrolytes (sodium) will aid water absorption in the duodenum but will slow the rate of water absorption in the jejunum due to movement of sodium into the lumen of the jejunum down concentration gradients, reducing effectiveness of water absorption [101] Hypertonic drinks result in net efflux of water from the body into the intestinal lumen, causing a net negative effect on water absorption and plasma volume [102] Hypotonic beverages are more effective than isotonic beverages for maximal water absorption [32, 103–106]
Carbohydrate concentration and type	Active co-transport of glucose and sodium facilitates the absorption of glucose and promotes the osmotic gradients that aid water absorption in the jejunum [39] Multiple transportable carbohydrate (e.g., glucose and fructose) in the jejunum creates a favourable osmotic gradient improving water absorption through solvent drag [39] Maltodextrin reduces osmolality when compared to glucose monomer potentially facilitating an increasing water uptake [1]
Sodium concentration	In the jejunum sodium is coactively transported with carbohydrates, amino acids, organic acids and bile salts [29] The role of sodium in active nutrient transport and water absorption is considered necessary in oral hydration solutions for clinical dehydration [107], but most evidence suggests negligible impact on absorption, plasma volume and retention during exercise [23, 32, 33] Solutions containing multiple carbohydrate types produce the greatest sodium absorption rates in the duodenojejunum and jejunum [1]
pH	Most beverages are acidic to maintain shelf life and palatability Acidosis may enhance water and sodium transport but not glucose [29]
Temperature	Ingested fluid is equilibrated to body temperature and at the level of the intestine [98] Temperature of the ingested fluid is likely to have minimal influence on intestinal absorption [98]
Sex	Limited research in gender differences and intestinal absorption

*mOsmol L*^*−1*^ osmolarity

Mechanistic measures of fluid flux in hydration research have included the net rate of appearance of isotopically labelled water (deuterium oxide, D_2_O) into the plasma compartment, the delta percent plasma volume (*d*PV), and ingested fluid delivery rate to the gut and the circulation from measures of gastric emptying and intestinal absorption (e.g., triple lumen segmental perfusion methodology). Each method has limitations for inference of hydration effects. D_2_O appearance captures only unidirectional fluid flux, and therefore is useful for the determination of fluid kinetics from ingestion to the appearance in the plasma or urine, but does not provide information on the net increase in fluid available to the body [34]. Intestinal absorption measured using segmental perfusion provides inference only to the specific segment of the intestine assayed, which is a limitation because absorption rates vary along the length of the intestine [1, 35]. Foundation physiological principles specify that the cellular component of blood has a fixed osmolality, while the extracellular compartment of the body fluid is in osmotic equilibrium between the vascular and interstitial compartments (Fig. 1). Therefore, change in *d*PV does not directly trace fluid absorption or compartmental transfer kinetics, but rather provides a direct measure of the net effect of ingested beverages on real-time central hydration status; *d*PV is also by far the most widely available parameter to permit large-scale analysis and inferential conclusions on hydration.

This brings us to the physical property of osmosis—the movement of fluid across semi-permeable membranes (permeable to the solvent, but not the solute)—to categorise the hydration properties of hypertonic, isotonic and hypotonic beverages [36–39]. Inference from intestinal absorption studies suggests benefits to hydration are possible from the ingestion of water and hypotonic and isotonic drinks, but conclusions as to the comparative benefits are equivocal when measures of *d*PV and contrasting solution compositional characteristics (CHO type, CHO concentration, salt concentration) are considered, leaving consensus on the most beneficial ingested drink tonicity for hydration unclear.

Therefore, the purpose of the current study was to determine the hydrating effects of ingested hypertonic, isotonic and hypotonic CHO-E drinks and non-CHO-E waters and water during continuous endurance exercise, as measured by *d*PV, by way of systematic meta-analysis. Hydration benefits were defined as a more positive directional gain of *d*PV over exercise time following drink ingestion. The present analysis was predicted to provide clarity on what beverage osmolality category may result in better rehydration for the implied purpose of offsetting potentially harmful effects of dehydration on cardiovascular function, thermoregulation and physical performance. While it is beyond the scope of the current review to cover optimal hydration strategies for sports or the effects of dehydration on sports performance, the new analysis may aid in future work towards these objectives.

## Methods

### Protocol and Registration

The meta-analysis was conducted and reported in accordance with the guidelines stipulated in the Preferred Reporting Items for Systematic Review and Meta Analyses (PRISMA) statement and SYRCLE protocol, and first registered with CAMARADES, publication date 27 April 2017. After initial analysis, re-evaluation of the available data led to a protocol refresh to include hypertonic drinks and water contrasts as described below.

### Search Strategy, Study Selection and Data Extraction

To retrieve relevant literature on hypotonic, isotonic and hypertonic solutions and water and their effects on hydration (*d*PV) during continuous exercise a systematic search was conducted from April 2017 to January 2018 and refreshed February to 30 June 2020 using the databases Scopus (including Medline), Web of Science, SPORTDiscus, PubMed and the Cochrane Central Register of Controlled Trials (Australia/New Zealand, North America). There was no limit to publication date. Search terms are listed in Table 3. Due to the potential for reference to animals appearing in the abstracts or full texts of relevant articles, the database searches were not restricted to studies conducted in humans. Studies published as reviews, abstracts, commentaries, etc., or studies for which the subject was irrelevant (i.e., on animals, in plants, in vitro) were excluded. No limitation was placed on the literature search with respect to language; however, the search terms naturally favoured English.

**Table 3:** Search terms used to retrieve literature

Keywords and search strings used for exposure and health outcomes	Hypotonic AND (isotonic OR hypertonic OR water) AND (absorption OR dehydrat* OR rehydrat* OR hydrat* OR plasma volume) Glucose AND (fructose OR sucrose OR maltodextrin*) AND (absorption OR hydrat* OR gastric emptying) carbohydrate AND (electrolyte OR sodium OR potassium*) AND (absorption OR dehydrat* OR rehydrat* OR hydrat* OR plasma volume) Osmolality AND (tonicity or concentration*) AND (absorption OR dehydrat* OR rehydrat* OR hydrat* OR plasma volume) Fluid AND (water OR carbohydrate OR sports drinks OR beverage*) AND (absorption OR dehydrat* OR rehydrat* OR hydrat* OR plasma volume)
Manual parameter refinements used to limit to Healthy Adult Humans	1. Check Human box 2. Independent runs to combine the above selection outcome with the words: patient, aged, elderly, child, adolescent; manually review selection for exclusions 3. Independent runs to combine the above selection outcome with the words: animal* or rat or rats or mice or mouse or dog or dogs or pig or pigs or rabbit* or hamster* or monkey* or rodent* or in vitro or ex vivo; manually review selection for exclusions
Parameters used to limit the search to intervention studies	The document/record type is not categorized as one of the following: patent, case study, book chapter, book, dissertation/thesis, biography, commentary, editorial, conference abstract, review, letter to the editor, English abstract, or citation-only The subject of the record is not categorized as one of the following: plants, spermatophyte, angiosperms, dicotyledons, monocotyledons, nonhuman, poaceae, cyperales, plant composition, fruits, or rosales

*Truncated word to optimize search efficiency

Titles and abstracts were screened for relevance by scanning for exclusion criteria by two reviewers (HK and CB). Full-text publications of potentially relevant studies were retrieved and reviewed for eligibility according to the inclusion criteria by the same reviewers. Reference lists of accepted articles were manually reviewed for relevant citations to supplement the search results. The data extracted included study characteristics, such as first author, year of publication, number of participants, and intervention characteristics (continuous exercise characteristics). Data sets were extracted in the form reported: text, tables, extracted from graphed data with a digital ruler. Authors were contacted to obtain missing data. Studies where only a single drink bolus was ingested were omitted because of different absorption kinetics and effects on *d*PV versus continuous drink boluses. Only datasets for continual regular drink ingestion, typically 10- to 15-min intervals, during continuous exercise were compiled for the analysis. Pre-exercise diet was unreported or euhydrated post-prandial. Contrasts with > 50 mEq L^−1^ sodium were excluded [33, 40, 41] because of non-specificity to current commercialised formulations (> 50 mEq L^−1^ sodium considered too salty to taste [26]), the impact of that much salt being moderate-large on plasma volume expansion and retention [41], and regulatory parameters [42].

### Inclusion and Exclusion Criteria

Included studies had to represent original research appearing in full-text format in peer-reviewed journals or an unpublished full study report of human intervention studies in healthy adults during continuous fixed-workload exercise. Studies had to compare the effect of ingestion of solutions formulated to different osmolality by altering either the CHO concentration and type and/or the electrolyte concentration or type. Dosing regimens had to be specified and were classified as: hypertonic (> 300 mOsmol kg^−1^), hypotonic (< 275 mOsmol kg^−1^), isotonic (275–300 mOsmol kg^−1^) or water from tap, mineral or beverage containing non-caloric flavouring, minerals or vitamins but without CHO at < 40 mOsmol kg^−1^ [43]. All drink osmolality values were measured or in some waters were unreported (Table 4; an analysis value of zero was assigned to all water treatments, see Sect. 2.5). Drinks were to be consumed orally in all trials and the ingestion rate (mL min^−1^) of solutions was standardised with ≥ 1 mL min^−1^ representing practical intake. Hydration was evaluated with plasma volume change (delta) from stable resting baseline (*d*PV), reported in a usable way, with > 1 during-exercise samples. Studies were also excluded if they represented duplicate or kin publications, the treatment or outcome was not appropriate or uninterpretable with regard to the outcome measures, subjects were dehydrated prior to exercise, the studies were not conducted in humans, or the ingested drinks contained protein.

**Table 4:** Summary of the methodology from the 28 studies identified from the database search that measured the effect of hypotonic, isotonic and hypertonic drinks or water on delta percent plasma volume (*d*PV) during continuous exercise, or the first continuous block of intermittent exercise

Study	*n*	Experimental protocol	Sampling protocol (min of exercise)	Ingestion protocol	Formulation	Overall mean *d*PV (SD)^a^
Gisolfi et al. [37]	7	85 min of cycling exercise at 63.3% *V*O_2max_. Environmental conditions: 22 °C	0, 20, 40, 60, 80	Ingestion of 23 mL kg^−1^ BM (mean total volume 1850 mL). Initial bolus of 20% (mean 370 mL) 5 min before exercise commenced. Additional 10% drinks (mean 185 mL) were then ingested every 10 min during exercise	Iso: G 2%, S 4%, Na^+^ 17.6 mEq L^−1^, K^+^ 3.3 mEq L^−1^, Osm 295 mOsmol kg^−1^	− 10.88 (1.57)
					Hypo: G 1%, S 2%, M 3%, Na^+^ 18.2 mEq L^−1^, K^+^ 3.3 mEq L^−1^, Osm 197 mOsmol kg^−1^ H_2_O	− 7.98 (0.93)
					Hyper: G 3.25%, F 2.75%, Na^+^ 17.2 mEq L^−1^, K^+^ 3.2 mEq L^−1^, Osm 414 mOsmol kg^−1^	− 9.70 (1.10)
					Water: Na^+^ 0.06 mEq L^−1^, K^+^ 0.25 mEq L^−1^, Osm 1 mOsmol kg^−1^	− 9.68 (1.13)
Gisolfi et al. [32]	6	85 min of cycling exercise at 65% *V*O_2max_. Environmental conditions: 22 °C, RH 40%	0, 20, 40, 60, 80	Ingestion of 23 mL kg^−1^ BM (mean total volume 1400 mL). Initial bolus of 20% (mean 280 mL) 5 min before exercise commenced. Additional 10% volumes (mean 140 mL) were then ingested every 10 min during exercise	Iso 1: G 2%, S 4%, Na^+^ 17 mEq L^−1^, K^+^ 3 mEq L^−1^, Osm 283 mOsmol kg^−1^	− 9.98 (3.08)^a^
					Iso 2: G 2%, S 1%, M 3%, Na^+^ 45 mEq L^−1^, K^+^ 3 mEq L^−1^, Osm 275 mOsmol kg^−1^	− 8.60 (3.15)^a^
					Hypo 1: G 2%, S 4%, Osm 245 mOsmol kg^−1^ H_2_O	− 9.98 (3.69)^a^
					Hypo 2: G 1%, S 1%, M 4%, Na^+^ 18 mEq L^−1^, K^+^ 3 mEq L^−1^, Osm 169 mOsmol kg^−1^	− 10.1 (3.68)^a^
					Hypo 3: M 6%, Na^+^ 47 mEq L^−1^, K^+^ 3 mEq L^−1^, Osm 176 mOsmol kg^−1^	− 10.50 (3.65)^a^
Rogers et al. [59]	5	85 min of cycling exercise at 60–65% *V*O_2max_. Environmental conditions: 22 °C	0, 20, 40, 60, 80 and post exercise	Ingestion of 23 mL kg^−1^ BM (mean total volume 1647 mL). Initial bolus of 20% (mean 327 mL) 5 min before ex commenced. Additional 10% volumes (mean 163 mL) were then ingested every 10 min during exercise	Iso: G 2%, S: 4%, Na^+^ 17.6 mEq L^−1^, K^+^ 3.1 mEq L^−1^, Osm: 280 mOsmol kg^−1^	− 9.56 (2.5)
					Hypo: G 1%, S: 2%, Na^+^ 17.2 mEq L^−1^, K^+^ 3.1 mEq L^−1^, Osm: 159 mOsmol kg^−1^	− 5.54 (1.1)
					Water (flavour matched): Osm: 4 mOsmol kg^−1^	− 6.50 (9.06)
Rowlands et al. [34]	11	120 min cycling at 55% *V*O_2max_ with 5 min rest after 60 min, followed by performance test. Environmental conditions: 19–21 °C, RH 50–60%. Note only samples 20–60 min were used in the analysis prior to the 5-min pause in exercise	0, 20, 40, 60, 85, 105, 125	Ingestion of 250 mL test solution every 15 min (total: 2000 mL)	Iso: G 0.6%, F: 0.6%, S: 6.4%, Na^+^ 12 mEq L^−1^, Osm: 281 mOsmol kg^−1^	− 10.8 (3.6)
					Hypo: G 1.45%, F 1.4%, S: 1.15%, Na^+^ 8 mEq L^−1^, Osm 220 mOsmol kg^−1^	− 10.1 (5.5)
					Hyper: G 1.6%, F 1.0%, S 3.5%, Na^+^ 21 mEq L^−1^, Osm 327 mOsmol kg^−1^	− 11.0 (3.11)
					Water (artificially sweetened, flavour matched): Osm 10 mOsmol kg^−1^	− 7.93 (4.66)
Ryan et al. [60]	8	180 min cycling at 60% *V*O_2max_. Environmental conditions: 33.2 °C, RH 29.8	0, 30, 60, 90, 120, 150, 180	Ingestion of 350 mL test solution every 20 min (total 3150 mL)	Iso: G 5%, Na^+^ 0.9 mEq L^−1^, Osm 300 mOsmol kg^−1^	− 6.3 (1.02)
					Hypo 1: M 3.2%, F: 1.8%, Na^+^ 9.2 mEq L^−1^, K^+^ 4 mEq L^−1^, Osm 156 mOsmol kg^−1^	− 1.95 (1.35)
					Hypo 2: M 5%, Na^+^ 3.8 mEq L^−1^, K^+^ 0.5 mEq L^−1^, Osm 82 mOsmol kg^−1^	− 3.92 (1.28)
Criswell et al. [61]	6	115 min cycling exercise at 65% *V*O_2max._ Environmental conditions: 29–30 °C, RH 58–66%	0, 30, 60, 90, 120	Ingestion of 400 mL test solution 20 min prior to ex, 275 mL immediately prior and every 15 min during exercise	Hypo: M 5%, F 2%, Na^+^ 8 mEq L^−1^, K^+^ 5 mEq L^−1^, Osm 250 mOsmol L^−1^	− 3.74 (4.8)
					Water (distilled, deionized): Osm 0 mOsmol L^−1^	− 6.38 (4.46)
Lambert et al. [35]	6	85 min of cycling exercise 60–65% *V*O_2max_ Environmental conditions: 22 °C	0, 15, 30, 45, 60, 75, 85	Ingestion of 23 mL kg^−1^ BM (mean total volume 1914 mL). Initial bolus of 20% (mean 383 mL) 5 min before ex commenced. Additional 10% volumes (mean 191 mL) were then ingested every 10 min during exercise	Iso: G 2%, S: 4%, Na^+^ 17.8 mEq L^−1^, K^+^ 3.1 mEq L^−1^, Osm 282 mOsmol kg^−1^	− 8.48 (3.18)
					Water (deionized, flavour matched): Osm 1 mOsmol kg^−1^	− 8.23 (2.82)
Owen et al. [57]	5	120 min treadmill exercise at 65% *V*O_2max._ Environmental conditions: 35 °C, RH started at 15% but increased to 30–50% by the end	0, 30, 60, 90, 120	Ingestion of 200 mL every 20 min during exercise	Hypo: M 10%, Na^+^ 6.7 mEq L^−1^, Osm 193.7 mmol kg^−1^	− 5.03 (4.0)^a^
					Hyper: G 10%, Na^+^ 1.4 mEq L^−1^, Osm 586.3 mmol kg^−1^	− 5.6 (3.5)^a^
Powers et al. [73]	9	Cycling exercise performed to fatigue (defined as a 10% decline in power output) at 85% *V*O_2max._ Environmental conditions: 20–22 °C, RH 53–60%	0, 5, 10, 20, 30, and at exhaustion	Ingestion of 210 mL immediately prior to and every 15 min during exercise	Hypo: M 7%, Na^+^ 9.2 mEq L^−1^ K^+^ 5.7 mEq L^−1^, Cl^−^: 9.0 mEq L^−1^, Osm 231.5 mOsmol kg^−1^	− 7.4 (5.5)
					Water (artificially sweetened, flavour matched): Na^+^ 3.1 mEq L^−1^, Osm 31.2 mOsmol kg^−1^	− 7.4 (4.0)
Yaspelkis et al. [62]	12	120 min cycling exercise at 48.8% *V*O_2max._ Environmental conditions: 33 °C, RH 51.7%	0, 5, 30, 60, 90, 120	Ingestion of 3 mL kg^−1^ BM (mean 219 mL) immediately prior to and every 15 min during exercise	Hypo 1: M 2%, Na^+^ 3.48 mEq L^−1^, K^+^ 1.53 mEq L^−1^, Osm 54 mOsmol L^−1^	− 7.3 (2.6)
					Hypo 2: M 5.75%, F: 2.75%, %, Na^+^ 5.20 mEq L^−1^, K^+^ 0.51 mEq L^−1^, Osm 273 mOsmol L^−1^	− 6.7 (3.9)
					Water: Osm 0 mOsmol L^−1^	− 8.2 (3.4)
Daries et al. [55]	8	90 min treadmill exercise at 65% *V*O_2max_ followed by a 30-min performance test. Environmental conditions: 25 °C, RH 55%. Participants drank two different volumes of the same solution	0, 15, 30, 45, 60, 75, 90, and at the end of the performance run	Ingestion of either 150 (mean 130 mL) or 350 (mean 300 mL) mL 70 kg BM^−1^ every 15–20 min	Iso (150 mL·70 kg^−1^BM): M 6.9%, Na^+^ 16 mEq L^−1^, Osm 278 mOsmol L^−1^	− 6.43 (3.3)^a^
					Iso (350 mL·70 kg^−1^BM): M 6.9%, Na^+^ 16 mEq L^−1^, Osm 281 mOsmol L^−1^	− 5.8 (2.8)^a^
Davis et al. [63]	19	120 min cycling at 75% *V*O_2max_ followed by a 30-min rest period before a performance test (time trial). Environmental conditions: 26.6–27.7 °C, RH 67–68%	dPV reported at 15 min before exercise began, 0, 12, 20, 30, 40, 75 min, end, 120 min	Ingestion of 275 mL every 20 min, from 15 min into exercise protocol	Hypo: G 2.5%, Na^+^ 10.2 mEq L^−1^, K^+^ 4.86 mEq L^−1^ Osm: 187 mOsmol L^−1^	− 5.56 (5.2)^a^
					Hyper: G 20 g L^−1^, 40 g L^−1^, Na^+^ 20.4 mEq L^−1^, K^+^ 3.44 mEq L^−1^. Osm 360 mOsmol L^−1^	− 6.6 (4.0)^a^
Kingwell et al. [74]	9	160 min cycling exercise at 65% *V*O_2max_. Environmental conditions: 20–22 °C	0, 40, 80, 120, 160	Ingestion of 200 mL in the first min of ex and at 20-min intervals thereafter	Hypo: M 10%, Osm 184 mOsmol kg^−1^	− 12.88 (4.8)
Febbraio et al. [54]	6	Cycling exercise to fatigue at 70% *V*O_2peak_. Environmental conditions: 33 °C, RH 20–30%	0, 20, 40	Ingestion of 250 mL immediately prior to and at 15-min intervals during exercise	Hyper: F 1.1%, G 1.9%, S 4.0%, M 7%, Osm 390 mOsmol L^−1^	− 11.7 (5.6)
					Hyper: F 1.1%, G 1.9%, S 4.0%, Osm 330 mOsmol L^−1^	− 9.7 (2.0)
	6	Cycling exercise to fatigue at 70% *V*O_2peak_ Environmental conditions: 5 °C, RH 50%	0, 20, 40, 60, 80	Ingestion of 250 mL immediately prior to and at 15-min intervals during exercise	Hyper: F 1.1%, G 1.9%, S 4.0%, M 7%, Osm: 390 mOsmol L^−1^	− 5.3 (3.6)
					Hyper: F 1.1%, G 1.9%, S 4.0%, Osm 330 mOsmol L^−1^	− 5.0 (3.3)
	6	Cycling exercise to fatigue at 70% *V*O_2peak_ Environmental conditions: 33 °C, RH 20–30%	0, 20, 40, 60, 80	Ingestion of 250 mL immediately prior to and at 15-min intervals during exercise	Hypo: F 0.55%, G 0.95%, S 2%, Osm 240 mOsmol L^−1^	− 6.5 (3.8)
					Hyper: F 1.1%, G 1.9%, S 4.0%, Osm 330 mOsmol L^−1^	− 7.6 (4.6)
Bishop et al. [64]	9	Cycling at 75% *V*O_2max_ to fatigue. Environmental conditions: 19.3 °C (0.2 °C), RH 58% (SD 2%)	0 and at fatigue	Ingestion of 5 mL kg^−1^ BM just prior to exercise and 2 mL kg^−1^ BM every 15 min during exercise	Hyper: G 5%, Na^+^ 60 mEq (as trisodium citrate 20 mmol L^−1^), artificially sweetened cordial, Osm 361 mOsm kg^−1^	− 6.2 (5.4)
Del Coso et al. [65]	7	Cycling at 63% *V*O_2max_ for 120 min in the heat. Environmental conditions: 36 °C (SD 1 °C), RH 29% (SD 1%)	0, 15, 50, 110	Ingestion of 813 mL immediately prior to starting exercise, followed by 407 mL at each of 8, 30, 60 and 90 min	Hyper 1: G 1.6%, F: 1%, S: 3.5%, Na^+^ 22 mEq L^−1^, K^+^ 3.4 mEq L^−1^, Mg^++^ 4.2 mEq L^−1^, Cl^−^ 13.3 mEq L^−1^, Osm 345 mOsm kg^−1^	− 7.6 (1.9)
					Hyper 2: G 1.4%, F: 2.2%, S: 4.8%, Na^+^ 22 mEq L^−1^, K^+^ 1.5 mEq L^−1^, Mg^++^ 1.4 mEq L^−1^, Cl^−^ 1.7 mEq L^−1^, Osm 337 mOsm kg^−1^	− 9.0 (1.8)
					Hyper 3: G 1.3%, F: 2.2%, S: 4.6%, Na^+^ 10 mEq L^−1^, K^+^ 0.7 mEq L^−1^, Ca^++^ 0.4 mEq L^−1^, Cl^−^ 9.6 mEq L^−1^, Osm 338 mOsm kg^−1^	− 8.9 (1.9)
					Mineral water: Na^+^ 0.9 mEq L^−1^, Mg^++^ 1.8 mEq L^−1^, Ca^++^ 4.4 mEq L^−1^, Cl^−^ 1.0 mEq L^−1^, HCO_3_^−^ 4.9 mmol L^−1^, Osm 15 mOsm kg^−1^	− 7.6 (2.2)
Deuster et al. [58]	10	Running at 60–65% *V*O_2max_ for 120 min. Environmental conditions not reported	0, 30, 60, 90, 120	Ingestion of 200 mL immediately prior to starting exercise, and at 30, 60, 90 min	Hypo: CHO: 7%, M 4.6%, F 2.5%, Na^+^ 9.3 mmol L^−1^, K^+^ 5.0 mmol L^−1^, Ca^++^ 1.1 mmol L^−1^, Mg^++^ 1.1 mmol L^−1^, Cl^−^ 9.7 mmol L^−1^, Osm 250 mOsmol kg^−1^	− 3.1 (6.2)
					Water (deionized): Osm 13 mOsmol L^−1^	− 1.1 (5.38)
Fallowfield et al. [56]	8	Running at 70% *V*O_2max_ to exhaustion following 5 min warm-up at 60% *V*O_2max_. Environmental conditions: 20 °C, RH unreported	0, and at exhaustion	Ingestion of 3.0 mL kg^−1^ BM immediately prior to exercise, followed by 2.0 mL kg^−1^ BM every 15 min during exercise	Water: Osmolality not reported	− 3.5 (3.11)
Gonzalez et al. [9]	7	Cycling at 62 ± 2% *V*O_2max_ for 120 min in the heat. Environmental conditions: 35.4 °C (SD 0.2 °C), RH 48% (SD 2%)	0, 5, 30, 60, 90, 120	Ingestions of 3.60 L (SD 0.14) consumed in equal volumes at 20, 35, 50, 65, 80, 95 min of exercise	Hypo: G 0.8%, S 1.1%, Na^+^ 6.3 mEq L^−1^, K^+^ 0.8 mEq L^−1^, Cl^−^ 4.1 mEq L^−1^, Osm 88 mOsmol L^−1^ (calculated)	− 6.8 (3.0)
Ishijimaet a et al. [66]	6	Cycling at 55% *V*O_2max_ for 90 min in a hot environment. Environmental conditions: 28 °C, RH 50%	0, 90	Ingestion of 384 mL every 15 min (total volume to equal BM loss during no fluid trial)	Mineral water: Na^+^ 13.0 mEq L^−1^, K^+^ 1.3 mEq L^−1^, Ca^++^ 0.6 mEq L^−1^, Mg^++^ 0.5 mEq L^−1^, Osm 25 mOsmol L^−1^	− 3.5 (2.94)
					Hypo: F 2.8%, M 0.2%, Na^+^ 12.6 mEq L^−1^, K^+^ 1.3 mEq L^−1^, Ca^++^ 0.7 mEq L^−1^, Mg^++^ 0.5 mEq L^−1^, Osm 203 mOsmol L^−1^	− 0.1 (3.18)
Lee et al. [67]	12	Cycling at 65% *V*O_2peak_ for 75 min. Environmental conditions: 32.1 °C (SD 0.3 °C), RH 66% (SD 1%)	0, 30, 60, 75	Ingestion of 1.5 mL kg^−1^ BM in equal volumes immediately before and at 15-min intervals during exercise	Water 1: Na^+^ 2 mEq L^−1^, Osm 2 mOsmol kg^−1^	− 11.2 (2.8)
					Water 2: Na^+^ 3 mEq L^−1^, Osm 25 mOsmol kg^−1^	− 10.4 (2.9)
					Hyper: S 4.8%, G 2.0%, Na^+^ 26 mEq L^−1^, K^+^: 2.9 mEq L^−1^, Osm 338 mOsmol kg^−1^	− 10.4 (4.0)
Massicotte et al. [75]	6	Cycling at 53 ± 2% *V*O_2max_ for 120 min. Environmental conditions: 21 °C (SD 1 °C), RH 45% (SD 5%)	0, 20, 40, 60, 80, 100, 120, but only 60 and 120 min reported dPV	Ingested as six equal volumes of 235 ± 34 mL at 0, 20, 40, 60, 80, 100 min	Hyper 1: G 7%, Osm 389 mOsmol kg^−1^ (calculated)	− 7.5 (11.5)
					Hyper 2: F 7%, Osm 389 mOsmol kg^−1^ (calculated)	− 3.5 (5.9)
Maughan et al. [68]	6	Cycling at 68% *V*O_2max_ for 60 min. Environmental conditions: 21–22 °C, RH 50–65%	0, 15, 30, 45, 60	Ingestion of 100 mL immediately prior to exercise and every 10 min thereafter to 50 min of exercise	Hyper 1: G 3.6%, NaCl 17 mmol L^−1^, KCl 20 mmol L^−1^, NaHCO_3_ 18 mmol L^−1^, Osm 310 mOsmol kg^−1^	− 7.0 (2.1)
					Hyper 2: M Glucose polymer with glucose content equivalent to 916 mmol L^−1^, insignificant amounts of citrate, lactate, caffeine, Osm 630 mOsmol kg^−1^	− 9.6 (2.2)
Maughan et al. [69]	12	Cycling at 70% of *V*O_2max_ to exhaustion. Environmental conditions: 21.0 °C (SD 0.1 °C), RH 21% (SD 1%)	0, 15, 30, 45, 60 and at exhaustion	Ingestion of 100 mL immediately before and at 10-min intervals during exercise	Distilled water: Osmolality unreported	
					Isotonic: G 3.6%, Na^+^ 35 mEq L^−1^, K^+^ 20 mEq L^−1^, Cl^−^ 37 mEq L^−1^, HCO_3_^−^ 18 mmol L^−1^, Osm: 310 mOsmol kg^−1^	− 4.7 (3.9)^a^
					Hypo: G 1.6%, Na^+^ 60 mEq L^−1^, K^+^ 25 mEq L^−1^, Cl^−^ 45 mEq L^−1^, citrate 20 mmol L^−1^, Osm: 240 mOsmol kg^−1^	− 3.8 (3.6)^a^
McConell et al. [72]	7	Cycling at 69 ± 1% *V*O_2peak_ for 120 min. Environmental conditions: 21.3 °C (SD 0.3 °C), RH 43% (SD 2%)	0, 20, 60, 120	Ingestion of six equal volumes totally either (1) 2.32 L (SD 0.10) (volume required to prevent BM loss) or (2) 1.16 L (SD 0.05) (half of the volume required to prevent BM loss) immediately before and at 20, 40, 60, 80, 100 min during exercise	Distilled deionized water (1): Osm unreported	− 10.1 (3.7)
					Distilled deionized water (2): Osm unreported	− 13.7 (2.4)
McConell et al. [76]	8	Cycling at 80 ± 1% *V*O_2peak_ for 45 min. Environmental conditions: 20.9 °C (SD 0.2 °C), RH 41% (SD 2%)	0, 10, 30, 45	Ingestion of four equal volumes totally (1) 1.47 L (SD 0.05) (volume required to prevent BM loss) or (2) 0.72 L (0.03) (half of the volume required to prevent BM loss) immediately before and at 15, 30, 43 min of exercise	Water 1: Osm unreported	− 9.0 (4.5)
					Water 2: Osm unreported	− 7.1 (4.3)
Murray et al. [70]	9	Cycling at 51.8% (SD 0.8%) *V*O_2peak_ for 90 min. Environmental Conditions: 30 °C, RH 45%	0, 13, 28, 43, 58, 68, 78, 88	Ingestion of 3.0 mL kg^−1^ LBM (162 ± 8 mL) at 15, 30, 45, 60 min during exercise	Water: Osm 27 mOsmol kg^−1^	− 6.3 (4.6)
					Hyper: G 6%, Na^+^ 20 mmol L^−1^, K^+^ 3.0 mmol L^−1^, Cl^−^ 11 mmol L^−1^, H_2_PO_4_^−^ 3 mM, citrate^−^: 9 mM, Osm 405 mOsmol kg^−1^	− 5.2 (3.7)
Sanders et al. [71]	6	Cycling at 65% *V*O_2peak_ for 90 min. Environmental conditions: 32 (SD 1 °C), RH 55 (SD 5%)	0, 10, 20, 40, 60, 80, 90	Ingestion of 400 mL water immediately prior to exercise and 100 mL every 10 min during exercise up to 80 min	Tap water: Osm unreported	− 10.0 (3.6)^a^

*BM* body mass, *LBM* lean body mass, *G* glucose, *S* sucrose, *F* fructose, *M* maltodextrin/glucose polymer, *Osm* osmolality (mOsmol kg^−1^ H_2_O) or osmolarity (mOsmol L^−1^ H_2_O) as reported within the cited study, *RH* relative humidity

^a^SD unavailable in literature, therefore estimated from meta-analysis of weighted study variance

### Study Quality

Included studies were assessed for quality based on the following assessment categories: purpose and/or hypotheses stated, confounders, participant background diet and lifestyle, exercise type, study duration and measurement time frame. Each study was independently assessed by two authors (CB and HK). Discrepancies were resolved by discussion, if necessary, with a third-party arbitrator present (DR).

### Statistical Analysis

Random-effects meta-analyses realised with mixed linear models (Proc Mixed in SAS Enterprise Guide 8.2.1) provided estimates and compatibility intervals of predicted values and effects; the effects were processed to give Bayesian posterior intervals and probabilities. All analysis codes and datasets are provided in Online Supplementary Material (OSM) 1–3.

#### Statistical Model

Each estimate was weighted by the inverse of the square of its standard error (SE), and the method of setting the residual variance to unity in Proc Mixed was used [44]. The fixed effects were the drink condition (Treatment: hyper-osmolar (hypertonic), iso-osmolar (isotonic), hypo-osmolar (hypotonic), water) interacted with time in tertiles of the dataset (TimeBin: < 30, 30–63, 63–180 min). Drink osmolarity was added as a linear numeric covariate (Treatment*TimeBin*DrinkOsM) to estimate the modifying effect of ingested drink osmolality on *d*PV of each CHO-containing drink. Predicted *d*PV and effects were adjusted to the mean osmolarity of the drinks in each condition. Three other linear numeric covariates interacted with condition and time were included: drink ingestion rate (the total volume ingested divided by the final *d*PV sampling time); heat index (calculated from the reported study ambient temperature and relative humidity using the tool provided by the National Weather Service at wpc.ncep.noaa.gov/html/heatindex.shtml); and metabolic rate (mean oxygen consumption rate during exercise in L min^−1^). Sweat rates, acclimation status, prior diet and urine production were not reported with sufficient consistency to allow inclusion in the model.

The random effects were: study identity (StudyID), to account for between-study heterogeneity; a unique identity for each exercise bout (ExptID) nested within StudyID, to account for Treatment as a repeated measure within studies between bouts; and a unique identity for each estimate (EstimateID) nested within StudyID, to account for TimeBin as a repeated measure within bouts. Variances were assumed to have a normal distribution, and negative values were permitted [45]. The variances of the random effects were combined and presented as a standard deviation (SD) representing unexplained typical uncertainty in the predicted mean *d*PV for a given drink at a given time in a new setting.

#### Inferential Framework

The Bayesian analysis promoted by Greenland [46] was used, since it allows practical analysis and interpretation of the prior as a probability distribution of the expected magnitude. Each effect on *d*PV was modified by a generic normally distributed weakly informative prior centred on zero with a 90% confidence interval (± 6.25% *d*PV; see below) that excluded very large effects on *d*PV drawn from the within-subject variability in endurance cycling performance and relationship to change in plasma volume. The prior distribution provided a reasonable physiological coverage of the possible mean effect. A more informative prior could not be justified since it would inevitably be biased by the current authors' knowledge of the published effects around the selected studies.

We used data for the relationship of change in PV after a (de)hydration intervention with change cycling performance time in healthy trained athletes, and information on the smallest meaningful effect on performance, to derive an estimate of the smallest important change in *d*PV from which to base statistical estimates of superior outcomes. The pooled average increase in PV (+ 3.6%, SD 8.8%) was associated with a pattern for improved time-trial performance (− 1.9%, SD 2.5%) [47]. The average coefficient of variation (CV%) for endurance road cycling time trial (TT) performance in a single event was 1.3% (90% CL 0.9–2.4%) [48]. The value for the smallest important effect that improves the chances of winning is 0.3 × CV% for performance [49]. This information provides an estimate for the smallest important effect (SIE) on *d*PV of 0.75% (i.e., 0.4 × 3.6/1.9). Accordingly, thresholds for moderate, large, very large and extremely large effect sizes were modelled and determined to be 0.9, 1.6, 2.5 and 4.0 times the CV, respectively [50].

For all outcomes, uncertainty was presented as 90% compatibility limits (CLs) (equivalent to Bayesian credibility) [51]. The unadjusted and Bayesian posterior probabilities were the area of the sampling *t*-distribution of the effect statistic relative to the SIE. Probabilities of increasing or superior (*p*_+_) or decreasing or inferior (*p*_–_) relative to the reference provided the compatibility measure that the effect is substantially different to the reference condition [46]. In a case where both *p*-values were < 0.05, the outcome represented equivalency. To assist with evidence-based qualification of outcomes, posterior probabilities that were compatible with an outcome > SIE were binned into conservative descriptors within a framework of information drawn from sport science and medicine [50] and climate change science [52], where: *p* = 0.25–0.75, about as likely (substantial) as not (equivalent); *p* = 0.75–0.95, likely; *p* = 0.95–0.995, very likely; *p* > 0.995, virtually certain. Effects with inadequate precision (i.e., *p*_–_ > 0.05 and *p*_+_ > 0.05) were noted as unclear or inconclusive.

#### Moderating Effects of Carbohydrate and Electrolyte Composition and Effective Intestinal Osmolality

Another appropriate analysis provided a model to account for the moderating effects of drink CHO composition on *d*PV. The model implementation was justified based on the premise of reducing all ingested CHO to monosaccharide functional equivalents providing the effective intestinal osmolality following (the rapid) digestion in the small intestine. This approach ignores the rapid water absorption through the duodenal leaky segment and the opposing secretion of fluid into the lumen with hypertonic intestinal contents [1, 38].

An analysis of the effect of the three CHO parameters concentration, format and transportable-CHO type was complexed by the multiple underlying levels and digestion and absorption interactions, all with numeric effects. Accordingly, CHO concentration was modelled as the total in grams percent volume (g/vol%). Carbohydrate format, whether monosaccharide, disaccharide or glucose polymer/maltodextrin, was coded in SAS using a two-level dummy variable, where monosaccharide = 0, and each of the disaccharide and glucose polymers were coded as a fraction of the gram percent concentration, respectively. This approach to coding had the effect of weighting the drink composition by make-up of the CHO format. The effective, post-digestion fructose and glucose (F:G) ratio of total ingested CHO was coded as the absolute g% ratio; for example, a solution comprising 20% fructose, 30% glucose polymer and 50% sucrose had an F:G value of 0.818. All four terms were interacted with TimeBin*Treatment.

Accounting for the di- and polysaccharide and electrolyte concentration allowed for an analysis of the ingested drink adjusted to the effective intestinal luminal osmolality following CHO digestion. This analysis was generated whereby the disaccharide concentration in mOsM kg^−1^ units (i.e., concentration in g 100 mL^−1^ divided by 180 g mol^−1^ times 1000/0.1) was doubled and the mass glucose polymer concentration divided by the molar mass of glucose (180 g mol^−1^). The total electrolyte composition was converted to mEq L^−1^ by multiplying the cation concentration by the valency to produce the total electrolyte mOsM concentration. Adding the CHO to the electrolyte osmolality produced the sum effective osmolality of the drink.

#### Generation of the Inverse Standard Error for Weighting

The inverse SE^2^ for the dependent variable was generated from the published or imputed SD for the dependent variable (*d*PV). Where the SD was not provided by study authors, the imputation was made by meta-regression of the natural log of the SD (logSD), by *d*PV adjusted for treatment, weighted by the inverse standard error for the logSD, which was 1/2DF (i.e., 1/(2*(subjectn-1)); W.G. Hopkins, personal communication, 2018). The number of imputed SD represented 17% of total SD. The SE was calculated from SD/SQRT(subjectn).

#### Heterogeneity

Heterogeneity was determined from the SD of the study and sample estimate residuals, expressed as the within-study average *t*-value, derived from the random effects solution. Visual inspection of a funnel plot of *t*-value versus SE for each study and individual estimate heterogeneity revealed data symmetry. No *t*-values exceeded 3.5, the threshold above which there is a 5% chance of at least one value in the absence of heterogeneity [45]. The rationale for choosing this approach was based on the argument that heterogeneity in a meta-analysis refers to real differences between effect magnitudes, which arise not from sampling variation but from moderation of the effect by differences between studies in subject characteristics, environmental factors, study design, measurement techniques or method of analysis [45]. The typical practice of testing for heterogeneity with the *I*^2^ statistic is problematic, because non-significance does not usually exclude the possibility of substantial heterogeneity, and neither the *I*^2^ nor the related *Q* statistic properly represent the magnitude of heterogeneity [53].

## Results

**Fig. 2:**
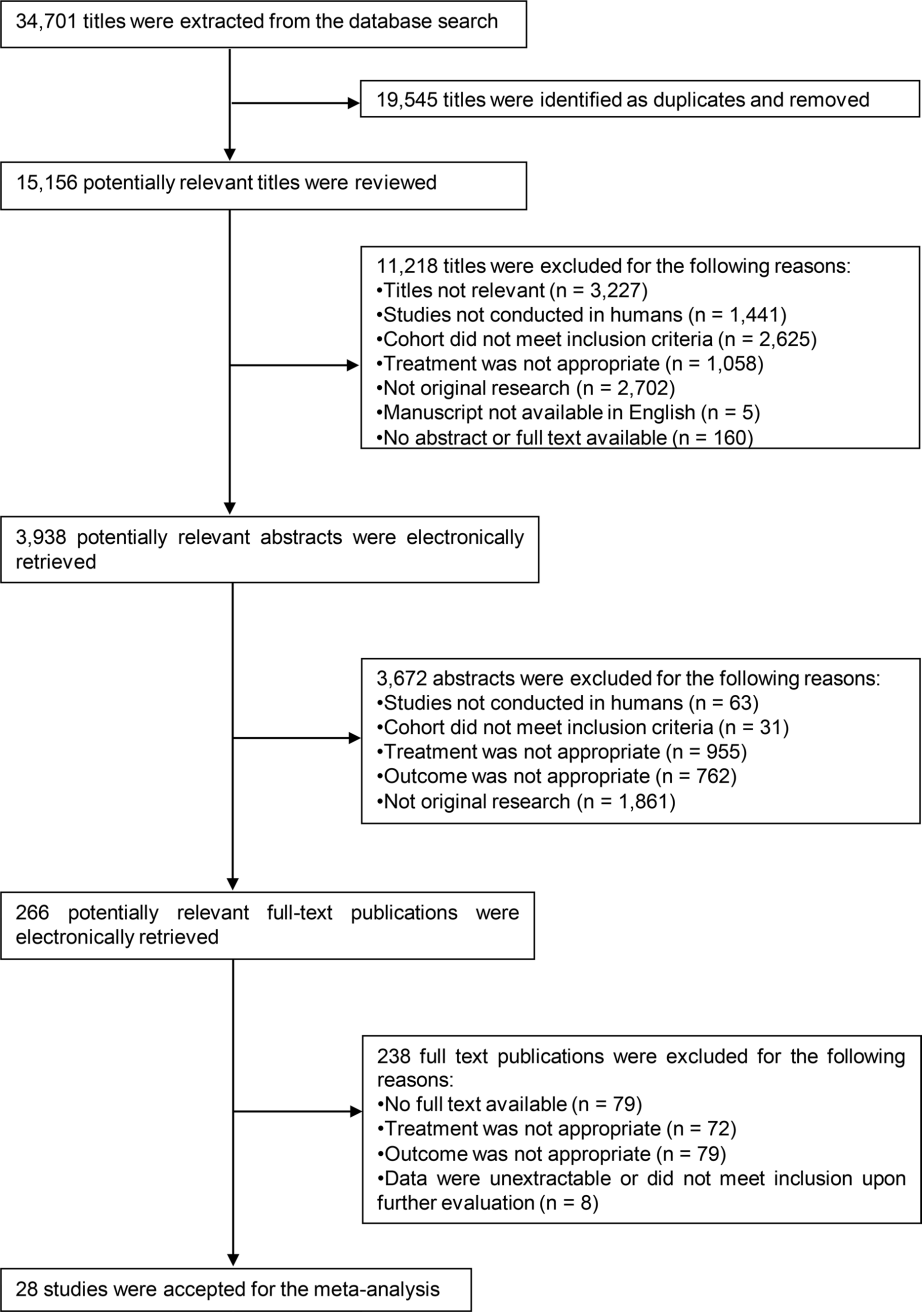
Preferred Reporting Items for Systematic Review and Meta Analyses (PRISMA) style summary of systematic review analysis workflow

The systematic literature search returned 266 potentially eligible studies (Fig. 2). We only considered the hydrating effects of ingested beverages during continuous exercise study settings due to methodological and physiological issues associated with disruption to plasma volume homeostasis and fluid shifts during intermittent exercise. The first bout of 60-min continuous exercise was included from one study using intermittent exercise [34]. Following review for eligibility and exclusions, a total of 28 publications with 29 study identities were extracted due to one publication containing three distinct samples and environmental conditions [54] (Table 4). Of the study identities, all utilised cycling ergometry except four, which utilised treadmill running [55–58]. There were insufficient studies to model the effect of exercise mode on outcomes. In total there were 68 unique drink treatment conditions of between one to five conditions per study, with a total of 258 measures of *d*PV over 0–180 min of continuous exercise utilised within the meta-analysis.

### Summary of Articles

Of the 28 trials included in the systematic review, 23 were randomized crossover trials (RXTs) [9, 32, 34, 35, 37, 55–72] and five were double blinded [34, 60, 61, 63, 67], while only two investigations were single blind [64, 70] (Table 4). Five trials were assumed to be non-RXT due to the authors not stating a randomized study design in the methods [54, 73–76]. The mean age of participants was 26.3 years, height 175.7 cm and weight 71.0 kg. The mean *V*O_2peak_ was 60.1 mL kg^−1^ min^−1^, with 13 studies recruiting healthy and non-athletes [32, 35, 37, 56, 58, 59, 64, 66–70, 75] and 15 studies indicating recruitment of well-trained or elite cyclists, runners and triathletes [9, 34, 54, 55, 57, 60–63, 65, 71–74, 76]. Average study sample size was eight, with data collected from 210 male and 16 female participants.

Twenty-four of the trials used a cycle ergometer and four used treadmill running [55–58]. The longest exercise duration was 180 min [60], with 12 prescribing 120 min [9, 34, 57, 58, 61–63, 65, 72, 74–76], four prescribing 90 min [55, 66, 70, 71] and six prescribing < 90 min [32, 35, 37, 59, 67, 68]. Four of the trials prescribed exercise until exhaustion at a set intensity of 70% *V*O_2max_ [54, 64, 69, 73]. All trials were sub-maximal at 48–85% *V*O_2max_.

Eleven trials reported participants completing the exercise task in hot conditions [9, 54, 57, 60–62, 65–67, 70, 71]: 30–36 °C, relative humidity 29–65%. The remaining trials reported thermoneutral conditions: 21–26 °C, relative humidity 21–55% (Table 4).

All studies provided participants with a beverage prior to exercise of between 100 and 813 mL. Thirteen of the trials provided servings at 15-min intervals, six trials used 10-min feeding intervals, six used 20-min intervals, and only two used 30-min intervals. The per-serving beverage volume ingested during exercise ranged from 100 to 407 mL (Table 4). Across all qualifying studies, the pre-exercise diet was reported as euhydrated post-prandial or unreported; accordingly, composition was not factored into the analysis due to lack of data.

### Heterogeneity and Descriptive Statistics for Modifying Covariates

The between-study variance (heterogeneity) of *d*PV derived from the random effects model (unadjusted for Bayesian prior) was expressed as a SD to describe the typical difference in the observed effect between studies. For the main effects meta-analysis model, adjusted for metabolic rate and average ingestion rate, the between-study SD (StudyID random effect) was 2.7% (90% CL 1.8, 3.3; SE 1.5%). These SDs should be doubled prior to inference relative to the reference SD of 2.62% used herein [50, 77]. Accordingly, the standardised mean difference for between-study heterogeneity was 1.03, or a moderate effect size. The within-study random variance between multiple levels of treatment (ExptID) expressed as an SD was 0.8% (90% CL 0.2, 1.2), while the within-study random variance between sample time points within a treatment (EstimateID) was 0.6% (90% CL 0.2, 0.8). Descriptive statistics for the modifying covariates are given in Table 5.

**Table 5:** Descriptive statistics for the study design-modifying covariates and drink-mediating parameters by ingested drink osmolality category with respect to the meta-analysis of the effect of ingested drink osmolality on *d*PV during continuous exercise

Variable; drink (*n*)	Hypertonic (67)	Hypotonic (77)	Isotonic (42)	Water (72)
Heat index	88.1 (14.6); 74.4, 121.8	86.1 (9.4); 76.4, 105.5	79.3 (4.7); 76.9, 90.0	83.5 (9.4); 74.4, 101.6
Temperature (°C)	24.3 (9.2); 5, 36	27.7 (5.7); 20, 35.4	24.1 (4.2); 20, 33.2	25.3 (5.2); 20, 36
Humidity (%)	42.6 (14.4); 21, 67.5	46.2 (10.7); 25, 67.5	46.8 (7.9); 29.8, 55	47.4 (11.2); 21, 65
Average drink ingestion rate (mL min^−1^)	14.7 (5.3); 7.2, 31	17 (5); 6.7, 30	18.1 (3.4); 8.7, 21.8	17.3 (6.9); 6.7, 32.7
Metabolic rate (L min^−1^)	2.7 (0.5); 1.6, 3.5	2.7 (0.5); 1.9, 3.8	2.6 (0.2); 2.2, 2.8	2.8 (0.6); 1.6, 4
Ingested drink osmolality (mOsM kg^−1^)	383.3 (87); 310, 630	178.4 (61.8); 54, 273	285.5 (8.5); 275, 300	9.6 (10.9); 0, 31.2
Total [carbohydrate] (g/vol%)	7.68 (3.49); 3.6, 16.5	5.49 (2.44); 1.92, 10	6.21 (0.71); 5, 7.6	0 (0); 0, 0
[Fructose] (g/vol%)	0.83 (1.33); 0, 7	0.63 (0.99); 0, 2.8	0.04 (0.15); 0, 0.59	0 (0); 0, 0
[Glucose] (g/vol%)	4.16 (3.92); 0, 16.5	0.53 (0.75); 0, 2.5	1.81 (1.58); 0, 5	0 (0); 0, 0
[Sucrose] (g/vol%)	2.08 (2.09); 0, 4.8	0.66 (1.08); 0, 4	2.26 (2.2); 0, 6.36	0 (0); 0, 0
[Glucose polymer] (g/vol%)	0.63 (2.01); 0, 7	3.67 (3.18); 0, 10	2.09 (3.03); 0, 6.9	0 (0); 0, 0
Fraction disaccharide	0.23 (0.28); 0, 0.67	0.17 (0.26); 0, 0.67	0.36 (0.34); 0, 0.84	0 (0); 0, 0
Fraction polysaccharide	0.04 (0.14); 0, 0.5	0.57 (0.41); 0, 1	0.31 (0.44); 0, 1	0 (0); 0, 0
Effective fructose:glucose ratio	0.37 (0.39); 0, 1.2	0.48 (1.59); 0, 14	0.29 (0.31); 0, 0.99	0 (0); 0, 0
Total [electrolyte] (mEq L^−1^)	51.6 (33.2); 0, 120	25.6 (22.0); 0, 100	37.2 (23.4); 1.8, 96	1.7 (4.8); 0, 32.1
Adjusted drink osmolality (mOsM kg^−1^)	488.3 (178.5); 320.0, 926.7	340.4 (135.4); 130.8, 578.9	392.0 (48.8); 289.6, 456.2	0 (0); 0, 0

Data values for each variable are the meta-analysed unweighted mean (SD); range (minimum, maximum), where n is the total number of single sample estimates. Metabolic rate is the mean oxygen consumption rate during exercise

### Main Effects

**Fig. 3:**
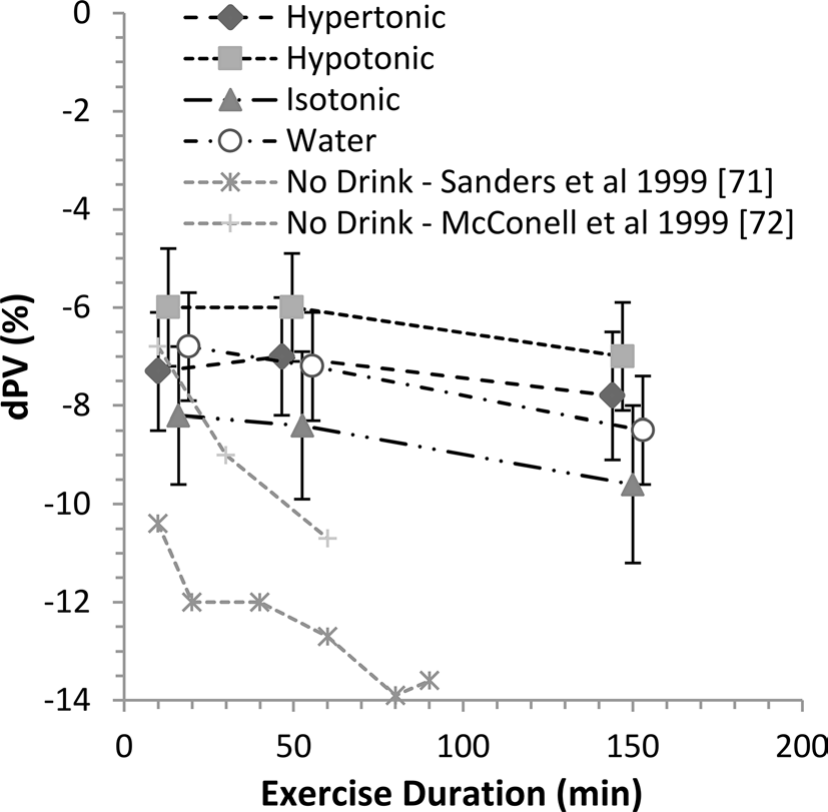
Effect of hypotonic, isotonic and hypertonic drinks and water ingestion during continuous endurance exercise on delta plasma volume (*d*PV) by TimeBin. Data are the estimates and 90% compatibility limits (CLs) from the full fixed-effects model adjusted for the modifiers metabolic rate and drink ingestion rate. Plot placement along the *x*-axis is in the mid-point of the TimeBin (< 30, 30–63, 63–180 min), with data points offset by 3–6 min for presentation clarity. Also shown is the mean *d*PV response when no drink is ingested under thermoneutral [76] and heat stress [71] environmental conditions

**Fig. 4:**
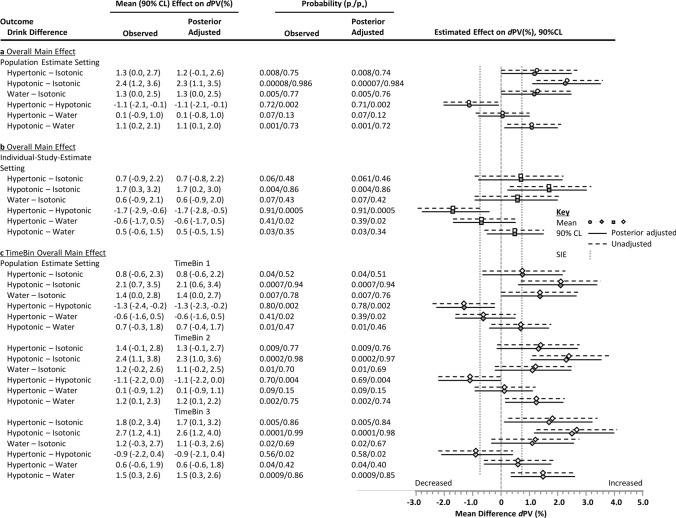
Differences in the effect of ingested hypotonic, isotonic and hypertonic drinks and water on delta plasma volume (*d*PV) during continuous exercise. Data are estimates and 90% compatibility limits (CLs) from the meta-analysis adjusted for metabolic rate and average drink ingestion rate partitioned into the unadjusted data-derived estimate and CL (unfilled symbols, hashed lines), and the posterior estimate and CL incorporating the weakly informative prior (filled symbols, solid lines). Panels include: **a** the overall effect for the population mean setting; **b** the overall effect for an individual-study setting (i.e., the fixed effect adjusted for random effects); and **c** the overall effect for the population mean setting apportioned by TimeBin, where the TimeBins were < 30, 30–63, and 63–180 min. The two directional probabilities were provided based upon the probability that a given drink contrast is compatible with a substantial increasing (*p*_+_) or decreasing (*p*_-_) effect relative to the smallest important effect (SIE) defined as 0.75% *d*PV. *p*-values are rounded to two significant figures relating to probability bins (see Sect. 2). Carbohydrate–electrolyte (CHO-E) drink categories were: hypertonic > 300 mOsmol·kg^−1^, hypotonic < 275 mOsmol·kg^−1^, isotonic 275–300 mOsmol·kg^−1^, water/non-CHO-E solutions < 40 mOsmol·kg^−1^

Estimates for the meta-analytical mean effect of the CHO-E and non-CHO-E drinks on *d*PV by TimeBin are shown in Fig. 3, where the greatest reduction in plasma volume during exercise occurred with isotonic and the least reduction with hypotonic drinks. The overall mean *d*PV estimates were: hypertonic − 7.4% (90% CL − 8.5, − 6.3); hypotonic − 6.3% (90% CL − 7.4, − 5.3); isotonic − 8.7% (90% CL − 10.1, − 7.4); water − 7.5% (90% CL − 8.5, − 6.4). Contrasts are shown in Fig. 4. With respect to the overall main effect in the population estimate setting, the adjusted mean *d*PV reduction was attenuated the most with the hypotonic drink, with the effect size and precision highest and most compatible with a substantial hydration effect relative to the isotonic drink, followed with lower probability by water/non-CHO-E and hypertonic drink contrasts. Time course (TimeBin) effects for the overall mean effect considering the precision of estimate of the mean were largely consistent with the overall mean effects, except for the hypotonic–water difference likely > 63 min, and the hypertonic–hypotonic difference about as likely as not > 30 min.

We also analysed the predicted individual-study estimate setting outcome—the mean single-study outcome after adjustment for study random effects. In this setting, the attenuating effect on *d*PV of the hypotonic drink relative to the isotonic and hypertonic drink contrasts remained likely, but the compatibility of a substantial effect of water relative to isotonic was made unclear, of hypotonic on water attenuated, and of hypertonic on water increased but remaining as likely as not (Fig. 4). Together, the individual-study estimate setting outcomes suggest unaccounted for study-effects were influencing the population estimate main effect, mostly in the water and hypertonic drink associated samples.

### Adjustment for Modifying Covariates

**Fig. 5:**
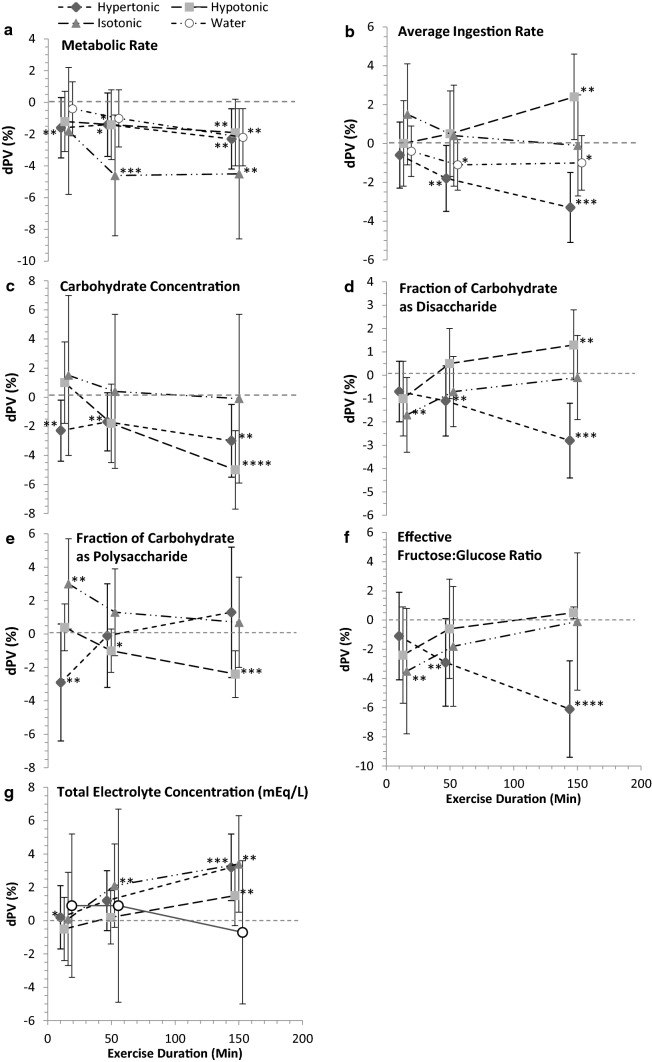
Modifying effects of study-design and drink compositional parameters on delta plasma volume (*d*PV) during continuous endurance exercise relating to hypotonic, isotonic, hypertonic drink and water ingestion. Effects represent the *d*PV response within a given drink category to 2SD of the modifiers: **a** metabolic rate and **b** drink ingestion rate, and the carbohydrate parameters **c** concentration, **d** disaccharide and **e** polysaccharide fractions, and **f** effective (after digestion) fructose:glucose ratio, and **g** total electrolyte concentration. Data are the estimate and 90% compatibility limits (CLs) from the full fixed effects model adjusted for ingested osmolality and the individual modifier, and the weakly informative Bayesian prior. Plot placement along the *x*-axis is in the mid-point of the TimeBin, with data points offset by 3–6 min for presentation clarity. Posterior probability of a substantial increase or decrease, relative to smallest important effect (SIE) (0.75% *d*PV), were binned for efficiency of data presentation denoted with star (*) symbols: *p* > 0.25 < 0.75*; *p* > 0.75 < 0.95**; *p* > 0.95 < 0.995***; *p* > 0.995****. Drink categories were hypertonic > 300 mOsmol·kg^−1^, hypotonic < 275 mOsmol·kg^−1^, isotonic 275–300 mOsmol·kg^−1^, water, non-carbohydrate solutions < 40 mOsmol·kg^−1^. *mEq/L* milliequivalents per litre

The independent modifying effects of the study-design parameters metabolic rate and drink ingestion rate, and the drink carbohydrate composition parameters and electrolyte concentration affecting *d*PV during continuous endurance exercise relating to the ingested CHO-E and non-CHO-E drink conditions are shown in Fig. 5. Effects on *d*PV presented are 2SDs of the modifier and are adjusted for the Bayesian prior. Shrinkage was observed in several of the larger magnitude estimates (OSM 3).

#### Exercise Metabolic Rate

Metabolic rate was associated with lower *d*PV; the overall largest effect was with isotonic drinks: isotonic − 4.0% (90% CL − 7.6, − 0.4); hypertonic − 1.8% (90% CL − 3.6, − 0.1); hypotonic − 1.6% (90% CL − 3.4, 0.3); and water − 1.2% (90% CL − 2.9, 0.4) (Fig. 5a).

#### Drink Ingestion Rate

Increasing the drink ingestion rate lowered overall mean *d*PV when hypertonic drinks (− 1.8%; 90% CL − 3.3, − 0.2) were ingested from the second tertial Timebin. However, ingesting the hypotonic drink had no impact on *d*PV early in exercise, but likely increased *d*PV in the final Timebin (Fig. 5b).

#### Drink Carbohydrate Composition

**Fig. 6:**
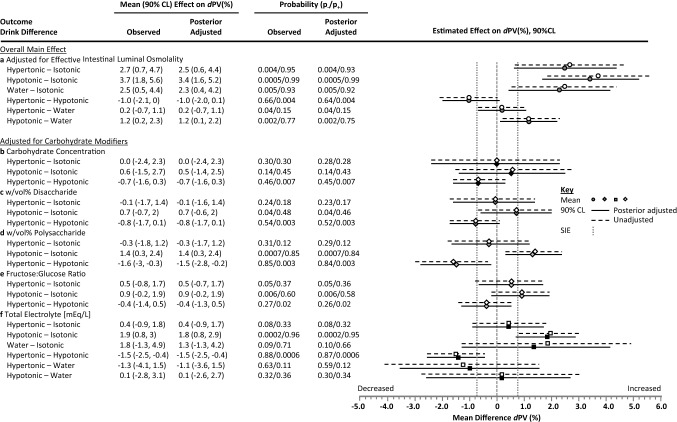
Differences in the effect of ingested hypotonic, isotonic and hypertonic drinks and water on delta plasma volume (*d*PV) during continuous exercise when adjusted for individual drink compositional modifiers. Data are estimates and 90% compatibility limits (CLs) from the meta-analysis adjusted for metabolic rate and average drink ingestion rate partitioned into the unadjusted observed mean and CL (unfilled symbols, hashed lines), and the posterior mean and CL incorporating the weakly-informative prior (filled symbols, solid lines). Panels include: **a** the overall main effect on *d*PV when adjusted for the effective intestinal luminal osmolality after carbohydrate disaccharide and polysaccharide hydrolysis, and the effect on *d*PV when adjusting for carbohydrate composition modifiers (see Fig. 5 for raw effect of modifiers on *d*PV) when osmolality is the ingested value for; **b** carbohydrate concentration (%); **c** carbohydrate weight (g)/volume percent (w/vol%) disaccharide; **d** w/vol% polysaccharide; **e** the effective fructose:glucose ratio; and **f** for the effect of total electrolyte concentration. The two directional probabilities were provided based upon the probability that a given drink contrast is compatible with a substantial increasing (*p*_+_) or decreasing (*p*_-_) effect relative to the smallest important effect (SIE) defined as 0.75% *d*PV. *p*-values are rounded to two significant figures relating to probability bins (see Sect. 2). Carbohydrate-electrolyte (CHO-E) drink categories were: hypertonic > 300 mOsmol·kg^−1^, hypotonic < 275 mOsmol·kg^−1^, isotonic 275–300 mOsmol·kg^−1^, water/non-CHO-E solutions < 40 mOsmol·kg^−1^. *mEq/L* milliequivalents per litre

Only during the third Timebin and with the hypotonic and isotonic drinks was there evidence that was likely compatible with a modifying effect of CHO concentration (Fig. 5c), but with no clear drink difference (Fig. 6b)*.* Evidence compatible with substantial effect sizes on *d*PV was found with the fractions of CHO as disaccharide (Fig. 5d) and polysaccharide (Figs. 5e, 6d), and the effective fructose:glucose ratio. Noteworthy was a small overall − 1.5% (90% CL − 2.8, − 0.3) reduction in *d*PV with the fraction of CHO as disaccharide when the drink was hypertonic (Fig. 5d), and the time effect of hypotonic (decreasing *d*PV) in response to the fraction of CHO as a polysaccharide (Fig. 5e).

#### Drink Electrolyte Concentration

Electrolytes were mostly favourable on *d*PV (Fig. 5g), with increases by the third TimeBin in isotonic, hypertonic and hypotonic drinks and a moderating effect in the hypotonic-water difference (Fig. 6f).

#### Effective Intestinal Luminal Osmolality

**Fig. 7:**
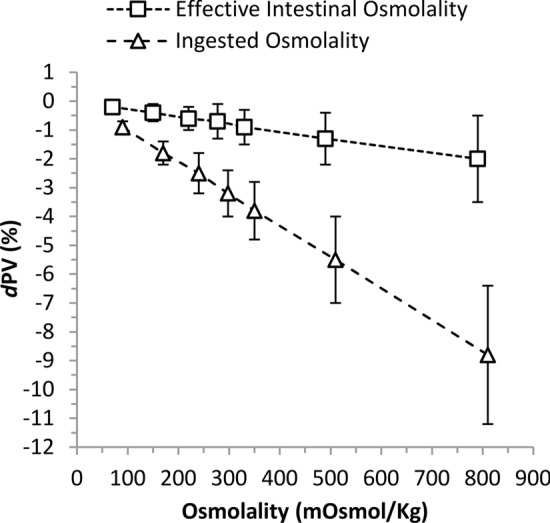
The effect of ingested drink osmolality (∆) and effective intestinal luminal osmolality after carbohydrate disaccharide and polysaccharide hydrolysis (□) on delta plasma volume (*d*PV). Data are mean estimate and 90% compatibility interval for eight representative levels of osmolality, derived from the random effects model with Treatment*Osmolality the moderator term with adjustment for metabolic rate and average ingestion rate. *mOsmol/kg* osmolality

The meta-analytical relationship between ingested osmolality and the effective intestinal luminal osmolality after di- and polysaccharide CHO digestion prior to absorption (ignoring any trans-epithelial water flux) is shown in Fig. 7. For every 100 mOsM kg^−1^ increase in ingested osmolality, *d*PV decreased by − 1.1% (90% CL − 1.4, − 0.8). Adjusting for the effect of CHO digestion—all CHO monosaccharide units and net effective osmolality—reduced a 100 mOsM kg^−1^ increase to a *d*PV decrease of − 0.3% (90% CL − 0.4, − 0.1).

Overall, the meta-analytical outcome of the effect of adjusting carbohydrate composition for effective intestinal osomolality on *d*PV (Fig. 6a) was to marginally increase the effect-size difference and compatibility (posterior probability) of a substantial difference between treatments; most evident, relative to the isotonic control. The adjustment effect was to increase by ~ 60% the width of the compatibility interval (from ~ 2.5% to ~ 4%) for the isotonic drink contrasts, which were most evidently affected by carbohydrate modifiers (Fig. 5) and contained the highest compositional representation of disaccharide (sucrose) content of the drinks evaluated (Table 5). Adjusting for the prior had a minor shrinkage effect on all data-derived estimates except the hypertonic-water contrast (Fig. 6; OSM 3).

## Discussion

The current analysis revealed that hypotonic CHO-E drinks ingested during continuous exercise very likely maintain better central hydration (*d*PV) when compared with the ingestion of isotonic CHO-E drinks. While evidence was less compatible, hypotonic CHO-E drinks were also more likely than not better central hydrators than hypertonic CHO-E drinks and non-CHO-E drinks/water, although the latter contrast was more likely trivial after accounting for between-study random effects. The attenuation of the reduction in *d*PV with the hypotonic drink was apparent, even after adjustment for exercise intensity (metabolic rate) and drink ingestion-rate modifiers, with a lesser reduction in *d*PV observed with hypertonic solutions and water. Integration of the Bayesian prior had no substantial impact on the data-driven primary meta-analysis. The prior did, however, cause substantial shrinkage within the 2SD of the modifier effect analysis, which is unsurprising given the effect size of the modifier. Overall, the analysis of probability finds evidence compatible with the conclusion that hypotonic CHO-E solutions provide the best hydration outcomes during exercise, even after considering remaining uncertainty after accounting for study design modifiers and random effects.

Some confusion in the literature and in commercial settings exists around the relative hydration efficacy of hypotonic versus isotonic drinks and water, because of what appear to be conflicting outcomes produced with different methodology and physiological measurement sites. Using triple-lumen intestinal segmental perfusion methodology at rest, Shi et al. [39] showed that both intestinal fluid absorption flux rate and net solute movement were higher with three 6% or 8% isotonic solutions that contained multiple-transportable CHO (MTC) as glucose, maltodextrin and sucrose controlled for [Na^+^], relative to hypotonic solutions with the same CHO concentrations but containing the single-transportable maltodextrin CHO source; however, in all cases the *d*PV pattern trended higher with the hypotonic solution, probably because of faster proximal intestinal fluid absorption indicated by the increase to iso-osmotic conditions in the distal segment (290–295 mOsmol kg^−1^). Gisolfi et al. [37] perfused hypotonic and isotonic solutions with 6% MTC into the proximal intestine of individuals at rest, with a drink osmolality difference due to maltodextrin replacing glucose. The intestinal fluid absorption flux rate was equivalent, but a relatively increased *d*PV was in favour of the hypotonic solution. In the distal intestine, as water flux follows solute transport across the apical membrane of the intestinal epithelial cell, the CHO facilitatory effects on intestinal absorption rates may be explained by solvent drag, whereby the transport of MTC creates a greater osmotic gradient [1, 39] supporting increased water draw, relative to a single transportable CHO (e.g., glucose) (Fig. 1). During continuous exercise, a drink containing MTC produced higher plasma D_2_O accumulation versus glucose [78]. With an isotonic drink comprising mostly sucrose and maltodextrin (Powerade, 7.6% CHO, 281 mOsmol kg^−1^) and a mildly hypertonic drink comprising sucrose, fructose and glucose (Gatorade, 6.0% CHO, 327 mOsmol kg^−1^), plasma D_2_O accumulation was substantially lower relative to a hypotonic drink (Mizone Rapid, 3.9% CHO, 220 mOsmol kg^−1^) comprising sucrose, fructose and glucose [34] (Table 4). The calculated intestinal osmolality of the isotonic and hypotonic drinks after digestion was 480 and 243 mOsmol kg^−1^, respectively, suggesting that the hypotonic drink remained functionally hypotonic, and that fluid absorption from isotonic solutions containing di- and polysaccharides will be relatively delayed versus a hypotonic solution, probably accounting for the lower *d*PV values.

Epithelial processes relating to water absorption by solvent drag, modified by CHO concentration and composition, were confirmed within the analysis (Fig. 5). As CHO drives osmotic pressure within the gut lumen, the effect of concentration was affirmed on *d*PV from the regression observation for the effective intestinal osmolality analysis: on average for every 100 mOsmol kg^−1^, *d*PV declined − 0.3% (Fig. 7). In support, higher CHO concentrations negatively correlated with water absorption in the proximal small intestine [1, 29]. Sucrose as an MTC promotes greater solute and water flux compared to isocaloric glucose [39], but the effect is likely limited to situations where glucose concentration is above the maximal glucose transporter (SGLT1) saturation capacity [79]. At carbohydrate concentration above the SGLT1, sucrose, being an MTC, promotes higher water flux since fructose is absorbed alternatively (GLUT5) [80]. However, results from perfusion studies are equivocal on the use of sucrose to increase water absorption. Some research has suggested equivalent fluid uptake rates from glucose and sucrose solutions [81], while in others the greatest benefit appears to arise from 6% versus 8% MTC and maltodextrin [1, 39].

An important observation was evidence compatible with a small benefit of hypotonic drinks to *d*PV compared to non-CHO-E/water drinks. On the other hand, non-CHO-E/water drinks also provided small benefit to hydration compared with isotonic drinks, although the contrast was made unclear after adjusting for study random effects (individual-study estimate setting analysis Fig. 4b), suggesting a level of within-study confounding bias contributed to the population estimate (Fig. 4a). Nevertheless, in the sensitivity analysis for the non-CHO-E/water-isotonic contrast (Fig. 6a), effect size and posterior probability remained compatible with a likely substantial benefit. These observations suggest that within the current data setting, both sports waters and hypotonic CHO-E drinks are superior to isotonic CHO-E drinks for hydrating during exercise. A possible explanation is that segmental fluid absorption is dictated by pore size, which progressively declines from proximal to distal. Therefore, faster water flux in the proximal duodenal segment (0–30 cm) will enhance fluid absorption from solutions of low carbohydrate concentration and tonicity facilitated by the osmotic gradient [1]. With a range of solutions comprising MTC and osmolality, there was no difference in intestinal water absorption rate versus water [38, 57, 59], which is probably secondary to rapid CHO-absorption rates in the jejunum promoting jejunal fluid absorption [1] that is secondary in turn to higher CHO-transporter density [24] compensating for the hypotonic effect at the proximal segment [37, 39].

Exercise intensity (metabolic rate covariate) substantially decreased *d*PV in all drinks, an effect twofold worse in the isotonic drink (Fig. 5a). Exercise, particularly intense exercise, can cause splanchnic hypoperfusion, driving gut mucosal ischemia [82], although effects on permeability are less clear [83]. A concentrated isotonic drink produced lower gut comfort versus a hypotonic drink [34]. Hypotonic drinks and water on the other hand increase unilateral water flux [34], which could attenuate the effects of blood-flow restriction on gut comfort during high-intensity exercise. Ingested fluid volume influences gastric emptying (GE), with a larger single volume emptied faster than a smaller up to 600 mL volume [30, 84]. Repeated ingestion maintains higher gastric volume and GE [30].

Including electrolytes, primarily sodium, in a sports drink increases body water retention through plasma Na^+^ concentration [40, 85–87]. In the current analysis, electrolyte concentration increased the effect on *d*PV with the hypertonic, isotonic and hypotonic drinks, but not water, later in exercise (Fig. 5g), suggesting electrolytes are an important mechanism influencing hydration in CHO-E drinks. On the other hand, the exclusion of sodium from glucose solutions did not influence water absorption in the small intestine [33]; the bi-directional movement of sodium across the mucosa of the proximal intestine possibly negates any pre-requisite for exogenous sodium in glucose solutions [1]. Gisolfi et al. [32] determined the effect of carbohydrate and Na^+^ concentration on intestinal absorption and plasma volume. Intestinal fluid absorption was faster with the hypotonic beverages, but plasma volume expansion was greater in the isotonic solution containing Na^+^ concentration 45 mEq L^−1^. A role for electrolytes, particularly Na^+^, however, is likely during prolonged exercise and in the heat. Body fluid loss can lead to relative hyponatremia since sweat Na^+^ concentration is ~ 20–80 mmol L^−1^ [88]. Failure to replace the excreted Na^+^, such as by consuming plain water, can lower plasma osmolality, increasing diuresis and leading to possible clinical deleterious effects [89]. During rehydration, increasing Na^+^ concentration by 1, 31, 40 and 50 mmol L^−1^ within a 2% sports drink stepwise increased rehydration, associated with reduced urine output [85]. In the current analysis, we excluded drinks with Na^+^ concentration > 50 mmol L^−1^ because of unfavourable palatability inhibiting drinking by reduced ad libitum consumption [90]. Furthermore, there are few commercial sports drinks or waters with [Na^+^] > 40 mmol L^−1^. Thus, the results from this meta-analysis support the addition of some electrolytes into sports drinks.

### Limitations

The *d*PV was assumed to represent net central body-water status during exercise justified theoretically from foundation physiological principles of compartmental fluid dynamics; whether this assumption holds requires empirical investigation. It is debatable whether the effort is warranted when compared to the comparatively understudied performance phenotype. Included studies were of limited sample size (majority *n* ≤ 12) and *d*PV was not the primary outcome in most, perhaps producing higher between-study errors compared to if *d*PV was a primary outcome. An estimate of the reliability for plasma volume (ICC 0.96) [91], the within-subject SD = SD*sqrt (1 − *r*) = 0.288%, where the composite SD for plasma volume was 1.44%, resulted in a traditional sample size of *n* = 18 (5% *α*, 20% *β* error) to detect 0.2SD, suggesting higher sample sizes are warranted. The effective intestinal osmolality analysis is based on an assumed universal intestinal luminal osmolality, which may not hold. Finally, the methods available for *d*PV provide differing levels of precision, suggesting the analysis method be considered as a random effect in future analyses.

## Conclusions

The regular ingestion of hypotonic CHO-E sports drinks during continuous exercise produced a greater attenuation of the exercise-mediated plasma volume decline, compared to isotonic and hypertonic drinks, and to non-CHO-E water/water. Additionally, the population estimate and sensitivity analyses provided outcomes compatible with sports waters hydrating better than isotonic CHO-E drinks. The sensitivity analysis suggested that isotonic drinks containing di- (sucrose) or polysaccharides present more like a hypertonic of equal carbohydrate concentration, reducing the hydration effect. The modifier analysis supported prior research indicating the inclusion of multiple transportable CHO in CHO-E drinks through an increased intestinal water absorption rate and net central hydration by increasing the threshold for transporter saturation and solvent drag. Electrolytes increase plasma volume retention and attenuate diuresis, assisting with retention of internal body fluid volume, which was seen to benefit hydration later in exercise in all CHO-E drinks. Isotonic drinks are most disadvantageous to hydration under conditions of higher metabolic rate. The greater the volume of hypertonic drink ingested, the worse the *d*PV outcome; but the opposite effect was true with the hypotonic drink as exercise duration progressed. Future research could investigate the efficacy of hypotonic CHO-E beverages on exercise performance during exercise in thermoneutral and hot conditions, relative to more concentrated solutions and non-CHO-E or water; drinks should be formulated with multiple-CHO types and contain sodium, and research designs should be adequately powered and use appropriate environmental conditions and exercise loads and reliable work tests (Table 6).

**Table 6:** Future research design considerations

Study design	Methods
Effect of hydration status on performance	Intervention on the mechanisms (e.g., *V*O_2max_, maximal cardiac output, blood volume, thermoregulation, muscle perfusion) Establish smallest meaningful effect size of *d*PV on cardiovascular performance and association with both continuous-type (e.g., endurance running, triathlon) and intermittent-type (e.g., international football, rugby, cricket, US football) of physical performance Consider effects of heat and cold stress, drink ingestion rate and exercise intensity
Determination of hydration rate	*d*PV Accumulation of deuterium oxide (D_2_O) following ingestion spike [34]
Large sample crossover study	Sufficient sample size to determine the effects of individual beverage components affecting hydration Drink contrasts, e.g.: 1. Hypotonic without sodium 2. Hypotonic with sodium (15–20 mmol L^−1^) 3. Isotonic drink with monosaccharides 4. Isotonic with di- and polysaccharides 5. Plain water (non-CHO water)
Beverage formulation	Hypotonic preferably in the range of 200–260 mOsmol L^−1^ appears optimal from review of the literature Must contain multiple transportable carbohydrates 6. The more favourable effect sizes in the literature are associated with fructose-maltodextrin blends. Superior to glucose/maltodextrin only approaching or over saturation concentration for glucose (0.5–0.8 g of ingested glucose/min range) 7. Favourable equivalent fructose:glucose ratio 0.8–1.0 to maximise carbohydrate absorption [108]
Ingestion rates	Standardised: mL W^−1^ to normalise for metabolic heat production Regular feedings to maintain optimal gastric emptying flow into duodenum during continuous exercise Ingestion rate to minimise dehydration because dehydration may impair gut blood flow and hence the impact of CHO on absorption Account for differences in body size, gender, metabolic and work rate
Exercise prescription	Cycle ergometer for applicability to a larger group of individuals Consideration of intermittent exercise at high intensities as it is likely to reduce intestinal absorption due to reducing gastric emptying rate [109] and disrupt the treatment differential established during steady-state exercise conditions [34] Consideration of methodological complication of intermittent exercise that during exercise, disruptions to the physiological *d*PV differential appear to neutralise the effect of drink treatment established during steady-state exercise [34], making investigation into the effects of drink osmolality on performance within intermittent sport structures probably more challenging
Biological sex	Inclusion of males and females Females are expected to be more variable (higher standard error) associated with endocrine and vascular physiological associated with menstrual cycle. Females have smaller stomachs so consideration of scaling beverage ingestion volume may be considered if volumes are over a threshold-volume of concern Study should be powered to ensure that measured effects of hydration can be detected between sexes Consideration of transgender individuals based on endocrine evaluation Consideration to balancing the cohort based on sex-influenced primary physiological, physical parameters
Environmental conditions	Initially in thermoneutral conditions (e.g., 20 °C, 50% humidity) Progression to heat stress conditions (e.g., 30 °C, 70% humidity) to reflect frequent sporting environmental conditions (e.g., Tokyo Olympics)

*dPV* delta percent plasma volume, *mOsmol L*^*−1*^ osmolarity

## Supplementary Information

Below is the link to the electronic supplementary material.

Supplementary file1 (DOCX 143 kb)

Supplementary file2 (XLSX 293 kb)

Supplementary file3 (XLSX 56 kb)

## Declarations

### Availability of Data

Results are presented within the article. The input and processed full-analysis datasets are in Online Supplemental Material 2 and 3.

### Code Availability

SAS code for the random effects meta-analysis is in given in Online Supplementary Material 1.

### Registration

Initial protocol registered with CAMARADES, publication date 27 April 2017.
