# Supplementary material for: The Hydrating Effects of Hypertonic, Isotonic and Hypotonic Sports Drinks and Waters on Central Hydration During Continuous Exercise: A Systematic Meta-Analysis and Perspective
Source: Sports Med. 2021 Oct 30;52(2):349–75. doi: 10.1007/s40279-021-01558-y (PMC8803723; doi:10.1007/s40279-021-01558-y)
Supplement: Supplementary file 1 — Supplementary file1 (DOCX 143 kb) [file 40279_2021_1558_MOESM1_ESM.docx]

**A. SAS code for primary mixed model**

filename in "C:\Users\dsrowlan\OneDrive - Massey University\Documents\Massey\Massey_2020\

Research\Frucor_Hydration Review\

ECSS_2018_Manuscript meta-analysis\New_Dec 2019\Working SAS_Feb 2020\data.txt";

options linesize=**150** pagesize=**500**;

**data** dat1;

infile in missover firstobs=**2** delimiter='09'x;

*RowNo=_n_;

length Study $ **30** ; *length Strgth $ 10 Magni $ 4;

input Study $ Source $ StudyID ExptID EstimateID contrast constrastn studytype $ Treat $ subjectn restexerciserecovery $

ex_type $ ex_mode $ ex_intensity_PCVOtwomax Votwomax_ml_kg_min Bodyweight Votwomax_l_min

metabolicrate thermal_conditions $ TempC Humidity time drink_vol

Total_fluid avg_ingestion_rate drinkOsM_measured drinkOsM_calc binOsm SD dPV SEM PV_Method $ Status $;

if time>**0**;

*if restexerciserecovery ne "recovery";

*if restexerciserecoveryne "rest";

*if ex_type ne "intermit";

*if dPV ne ".";

if StudyID ne **26**;*was repetition of Duester 1992 coded as 1991;

if StudyID ne **19**;*Below 1995 unusalbe data;

if StudyID ne **20**;*Bishop 2000 unusalbe data;

if StudyID ne **39**;*Utter t>3.5;

*if StudyID ne 40;*Utter t>3.5 and signle study data point;

if StudyID ne **22**;*CheeKeong not Dill and Costill method, and running and water;

if StudyID ne **37**;*Ryan 1998;

if StudyID ne **28**;*Galloway Maughan 1998;

if StudyID ne **25**;*Del Coso 2009;

*inclusion criterion to add - more that one primary outcome during continuous exercise;

*if StudyID ne 27;*Fellowfield. only 1 data point and it was crap;

*if StudyID ne 30;*Fellowfield. only 1 data point and it was crap;

if EstimateID ne **607**; *gonzalaz hypertonic;

if EstimateID ne **608**; *gonzalaz hypertonic;

if EstimateID ne **609**; *gonzalaz hypertonic;

if EstimateID ne **610**; *gonzalaz hypertonic;

if EstimateID ne **611**; *gonzalaz hypertonic;

if StudyID=**40** and ExptID=**2** then delete;

if StudyID ne **24**;*Davis et al 1992. dPV relative to first exercise sample;

*if bindosm ne "3";

*select when analysing overal mean effects for all measures only excluding time effects. 0 when want time ffects;

*if CHOcovar=0;

drop starting_hydration_state restexerciserecovery ex_type;

*if ex_mode ne "run";

*if thermal_conditions ne "heat";

*if thermal_conditions ne "cool";

*if StudyID=8 then TempC=22;*rest;

*if StudyID=9 then TempC=22;*rest;

*if StudyID=8 then thermal_conditions="neutral";

*if StudyID=9 then thermal_conditions="neutral";

*if restexerciserecovery="rest" then ex_type="rest";

*if ex_type ne "rest";

*VOkg=metabolicrate/Bodyweight;

*folddPV= *converting dPV into fold then log transform;

*Outliers raw residuals contrasts with t score > 3.5 for publication bias;

/*

if contrast=34 and time=30 then delete;

if contrast=33 and time=30 then delete;

if contrast=29 and time=120 then delete;

if contrast=33 and time=5 then delete;

if contrast=34 and time=5 then delete;

*/

*if treat="Water" and drinkOsM<50 then delete;

if metabolicrate="." then metabolicrate=ex_intensity_PCVOtwomax/**100***Votwomax_l_min;

**run**;

*proc print data=dat1;

*run;

*first metaregress logSD = meandPV covariates to generate outp for missing logSD from dPV;

**Proc** **means** data=dat1;

var SD;

output out=inputdatSDcount n=n;

**run**;

**data** dat2;

set dat1;

logSD=log(SD);*SD is either published or calculated from published SEM;

SElogSD=**1**/(**2***(subjectn-**1**));*/2DF, here WiSD -s the SE, is the SE for the log of the SD, or weighting factor. So, SE=1/2DF.

to derive the weighting factor => 1/SE^2 = 4*DF^2;

InvSE2=**1**/SElogSD****2**;

folddPV=(**100**+dPV)/**100**;

logdPV=**100***log(folddPV);

DrinkOsM=DrinkOsM_measured;

if DrinkOsM_measured="." then DrinkOsM=drinkOsM_calc;

*if Treat="Water" then DrinkOsM=0 else DrinkOsM=DrinkOsM-287.5; *standardising OsM to mean body OsM;

if Treat="Water" and DrinkOsM>**40** then delete;*minerals waters are 3-39 mOsM and omitting problem drinks. https://sgsm.ch/fileadmin/user_upload/Zeitschrift/54-2006-3/Osmolality_54_3_06.pdf;

if Treat="Water" then

do;

DrinkOsM=**0**;

end;

else

do;

DrinkOsM=DrinkOsM-**287.5**;

end;

**run**;

ods listing close;

*Treat: hyper hypo iso water;

*to generate a meta-analytical dataset that contains residuals for each estimateID of dPV, which can then be used as the imputed sample SE;

**proc** **mixed** covtest data=dat2 cl alpha=**0.1**;*nobound;

class StudyID Treat; *covariates TempC Humidity avg_ingestion_rate ;

weight InvSE2;

model logSD=dPV Treat/cl s noint DDFM=sat OUTP=residuals alpha=**0.1**;*first model Oct practice;

estimate "SD @ -10 dPV exerc iso" dPV -**10** treat **0** **0** **1** **0**/Cl alpha=**0.1**;* estimate "SD @ -10 dPV exerc iso" int 1 dPV -10 restexerciserecovery 1 0 treat 0 1/Cl alpha=0.1 **use this if don't have noint in the model statement. noint allows for individual intercepts and slopes for each level of nomimals Important.;

estimate "chge for 1 unit cnge indPV" dPV **1**/Cl alpha=**0.1**;

estimate "chge for 5 unit cnge indPV" dPV **5**/Cl alpha=**0.1**;

estimate "chge 1 unit cnge hypo-iso" Treat **1** -**1**/Cl alpha=**0.1**;

*estimate "chge 1 unit cnge for exercise-rest" restexerciserecovery 1 -1/Cl alpha=0.1;

ods output residuals=outp;

ods output estimates=est;

**run**;

ods listing;

**data** est1;

set est;

array a estimate lower upper;

do over a;

a=exp(a);

end;

**proc** **print** data=est1;

**run**;

*proc print data=residuals;

*run;

**data** dat3;

set residuals;

if logSD="." then logSD=Pred;*pulls out the imputed residual for dPV with mssing SD;

SD=exp(logSD);

Var=SD*SD;

SE=SD/SQRT(subjectn);

WiRE=**1**/(SE*SE);

*centeredtime=(time-87.5)/180;*for hypo vs iso only;

**run**;

*proc print data=dat3;

*run;

**Proc** **means** data=dat3;

var SE;

output out=inputdatSEcount n=n;

**run**;

* to generate mean values for temp and hum for studyID with missing;

**data** dat5;

set dat3;

if thermal_conditions ne "heat";

if thermal_conditions ne "cool";

*if Humidity ne ".";

**run**;

**proc** **means** noprint data=dat5;

var Humidity TempC;

by studyID;

output out=neutralhumidityout mean=;*mean TempC for neutral conditions;

**Proc** **sort** data=dat5;

by StudyID;

**run**;

**proc** **means** noprint data=neutralhumidityout;

var Humidity TempC;

*weight meanWiRE;

output out=neutralhumidityoutbystudy mean=;*mean TempC for neutral conditions;

**data** dat61;

set neutralhumidityoutbystudy;

meanhumidity=Humidity;

meanTempC=TempC;

**run**;

**data** dat6;

set dat3;

if _n_=**1** then

set dat61(keep=meanhumidity meanTempC);

if Humidity="." then Humidity=meanhumidity;

**run**;

**data** dat62;

set dat6;

if TempC="." then TempC=meanTempC;

**run**;

**heat stress index WBGT;

*www.weather.gov/media/epz/wxcalc/heatIndex.pdf;

*www.weather.gov/media/epz/wxcalc/tempConvert.pdf;

**data** dat7;

set dat62;

*set dat3;

tempF=(**9**/**5**)*TempC+**32**;*temp converstion to F;

heatindex=-**42.379**+(**2.04901523***tempF)+(**10.14333127***Humidity)-(**0.22475541***tempF*Humidity)-(**6.83783**/**1000***tempF*tempF)

-(**5.481717**/**100***Humidity*Humidity)+(**1.22874**/**1000***tempF*tempF*Humidity)

+(**8.5282**/**10000***tempF*Humidity*Humidity)-(**1.99**/**1000000***tempF*tempF*Humidity*Humidity);

**run**;

**Proc** **corr** data=dat7;

var TempC Humidity heatindex avg_ingestion_rate dPV DrinkOsM metabolicrate;

**run**;

title "to get time bins";

**proc** **freq** data=dat7;

tables time;

*by ex_type;

**run**;

**data** dat9;

set dat7;

length TimeBin $ **8**;*sets the character limits for output;

if time<**31** then TimeBin="1.<30 ";

if time>**31** and time<**63** then TimeBin="2.30-63 ";

if time>**63** then TimeBin="3.63-180 ";

**run**;

/*

data dat9;

set dat7;

length TimeBin $ 8;*sets the character limits for output;

if time<31 then TimeBin="1.<30 ";

if time>31 and time<60 then TimeBin="2.30-60 ";

if time>60 then TimeBin="3.60-225 ";

run;

*/

/*

proc sort data=dat9;

by Treat;

run;

proc means noprint data=dat9 maxdec=1;

var dPV;

weight WiRE;

by Treat;

output out=meansbytreat mean=mean stddev=SD;

run;

Proc print data=meansbytreat;

run;

*/

/*

data dat10;

set dat9;

length TreatBin $ 10;*sets the character limits for output;

if drinkOsM <185 then TreatBin="1.<185 ";

if 185< drinkOsM <274 then TreatBin ="2.185-275 ";

if drinkOsM >274 then TreatBin ="3.275-300 ";

run;

*/

/*

data datisomeans;

set dat3;

*if Treat="Hypo";*aim is to explore the premise that isotonic is the best hydration drink during exercise;

run;

proc means noprint data=datisomeans;

var Var;

weight WiRE;

output out=metaVar mean=aveVar;*mean TempC for neutral conditions;

Data datmetaSD;

set metaVar;

avemetaSD=SQRT(aveVar);

run;

title "average overall weighted metaSD";

proc print data=datmetaSD;

var avemetaSD;

run;

*/

**proc** **sort** data=dat9;

by TimeBin Treat;

**run**;

**proc** **means** noprint data=dat9 maxdec=**1**;

var Var;

*weight WiRE;

by TimeBin Treat;

output out=metaSDVar mean=mean;

**run**;

**data** metaSDVar2;

set metaSDVar;

metaSD=sqrt(mean);

by TimeBin Treat;

**run**;

title10 "unweighted mean SD of timebin treat";

**proc** **print** data=metaSDVar2;

var TimeBin Treat metaSD;

**run**;

**proc** **sort** data=dat9;

by TimeBin Treat;

**run**;

**proc** **means** noprint data=dat9 maxdec=**1**;

var dPV;

weight WiRE;

by TimeBin Treat;

output out=metameandPV mean=mean;

**run**;

**data** metameandPV2;

set metameandPV;

metadPV=mean;

by TimeBin Treat;

**run**;

title "meta mean weighted dPV timebin treat";

**proc** **print** data=metameandPV2;

var TimeBin Treat metadPV;

**run**;

**proc** **sort** data=dat9;

by Treat;

**run**;

**proc** **means** noprint data=dat9 maxdec=**1**;

var Var;

*weight WiRE;

by Treat;

output out=metaSDVaroverall mean=mean;

**run**;

**data** metaSDVar2overall;

set metaSDVaroverall;

metaSD=sqrt(mean);

by Treat;

**run**;

title "unweighted mean SD by treat";*SDs should not be weighted;

**proc** **print** data=metaSDVar2overall;

var Treat metaSD;

**run**;

**proc** **means** noprint data=dat9 maxdec=**1**;

var Var;

weight WiRE;

output out=metaSDVaroverall mean=mean;

**run**;

**data** metaSDVar2overall;

set metaSDVaroverall;

metaSD=sqrt(mean);

**run**;

title " weighted mean SD overall all treats";

**proc** **print** data=metaSDVar2overall;

var metaSD;

**run**;

**proc** **means** noprint data=dat9 maxdec=**1**;

var dPV;

weight WiRE;

by Treat;

output out=metameandPVoverall mean=mean;

**run**;

**data** metameandPV2overall;

set metameandPVoverall;

metadPV=mean;

by Treat;

**run**;

title "meta mean dPV timebin treat";

**proc** **print** data=metameandPV2overall;

var Treat metadPV;

**run**;

/*

Data datmetaSD;

set metaSDVar;

avemetaSD=SQRT(aveVar);

run;

*/

title8 "Raw Means ALL for covariates";

**proc** **means** data=dat9 maxdec=**1**;

var heatindex TempC Humidity avg_ingestion_rate metabolicrate drinkOsM;*drinkOsM;

*by Treat;

output out=meansallcovariates mean=mean stddev=SD min=min max=max;

**run**;

**proc** **sort** data=dat9;

by Treat;

**run**;

title9 "Raw Means BY TREAT for covariates";

**proc** **means** data=dat9 maxdec=**1**;

var heatindex TempC Humidity avg_ingestion_rate metabolicrate DrinkOSM;*drinkOsM;

by Treat;

output out=meanstreatcovariates mean=mean stddev=SD min=min max=max;

**run**;

**proc** **standard** data=dat9 out=dat11 mean=**0** std=**0.5**;

var heatindex TempC Humidity metabolicrate avg_ingestion_rate ;*for the coefficients, 1 gives 2SD; *drinkOsM not standarised;

*by ex_type;

**run**;

*proc print data=dat10;

*run;

/*

data predict;

StudyID=0;

EstimateID=0;

ExptID=0;

*xVarPost2=0;

*xVarPost3=0;

*if Treat=2 then xVarPost2=1;

*if Treat=3 then xVarPost3=1;

*SameSubjects=1;

*GrpConExp="Expt";

*Diet=0;

*Intensity="2.Mod";

*Total10h=1.5;

*&dep=&dep1;

*if &logflag then &dep=100*log(&dep);

*/

**data** dat22;

set dat11;*predict;

if dPV ne ".";

**run**;

*Primary model;

ods listing close;

**proc** **mixed** covtest data=dat22 cl alpha=**0.1** nobound CONVH=**1E-6** convf=**1E-6**;*nobound;*forcing it to converge at 6 dp;

class StudyID TimeBin ExptID Treat EstimateID ex_mode;

weight WiRE;

*model dPV=TimeBin*treat/noint cl s DDFM=satalpha=0.1;

*standarised covariates for later avg_ingestion_rate*Treat heatindex*Treat metabolicrate*Treat;

*modifying and mediating covariates; *heatindex was found not to be an important modifer, so not continued with;

*model dPV=TimeBin*Treat TimeBin*Treat*DrinkOsM TimeBin*Treat*avg_ingestion_rate TimeBin*Treat*avg_ingestion_rate TimeBin*Treat*heatindex/noint cl DDFM=sat outp=pred outpm=predm residual alpha=0.1; *model to generate residuals for t vs SE plot for assymetry. When running turn off random effects;

*model dPV=TimeBin*Treat TimeBin*Treat*DrinkOsM TimeBin*Treat*metabolicrate/noint cl DDFM=sat outp=pred outpm=predm residual alpha=0.1;

*model dPV=TimeBin*Treat TimeBin*Treat*DrinkOsM TimeBin*Treat*avg_ingestion_rate/noint cl DDFM=sat outp=pred outpm=predm residual alpha=0.1;

*model dPV=TimeBin*Treat TimeBin*Treat*DrinkOsM TimeBin*Treat*heatindex/noint cl DDFM=sat outp=pred outpm=predm residual alpha=0.1;

*model dPV=TimeBin*Treat TimeBin*Treat*DrinkOsM/noint cl s DDFM=sat outp=pred outpm=predm residual alpha=0.1;

*model dPV=TimeBin*Treat TimeBin*Treat*heatindex TimeBin*Treat*metabolicrate TimeBin*Treat*avg_ingestion_rate/noint cl s DDFM=sat outp=pred outpm=predm alpha=0.1;

model dPV=TimeBin*Treat TimeBin*Treat*DrinkOsM TimeBin*Treat*metabolicrate TimeBin*Treat*avg_ingestion_rate/noint cl DDFM=sat outp=pred outpm=predm alpha=**0.1**;

random StudyID StudyID*ExptID StudyID*EstimateID/;

Parms **5** **1** **1** **1**/hold=**4**;

*clamping residual covariance to 1 means the weighting factor is the random effect for each dependent variable. Residual can be negative variance in this instance;

*references: Cassar et al 2016 Human Reproduction, pp. 1–13, 2016 doi:10.1093/humrep/dew243;

*Weston 2014 Sports Med (2014) 44:1005–1017, DOI 10.1007/s40279-014-0180-z;

*Yang 2003 A review of random effects modelling in SAS (release 8.2);

****do for Osm e.g. 350 600 1200... 220 150. range of OsM generally2SD accros studies, but something sensible;

/*

random StudyID StudyID*ExptID StudyID*EstimateID/s;

Parms 5 1 1 1/hold=4;

…provides the random effect for ExptID and EstimateID nested within StudyID, and provides the identical random effect solution to the following code, which may be more commonly used in SAS…

random StudyID /s;

random ExptID(StudyID) /s;

random EstimateID(StudyID) /s;

Parms 5 1 1 1/hold=4;

Note, the Parms 5 1 1 1/hold=4; is required to hold the residual error at unity to permit the Study weighting factor (1/SE) to become the residual error for each dependent variable.

*/

estimate " ";

estimate "modifying effect of covars on dPV";;

estimate "Effects of 2SD on dPV";

estimate "DrinkOsM all" TimeBin*Treat*DrinkOsM **174** **126.6** **17** **0** **174** **126.6** **17** **0** **174** **126.6** **17** **0**/cl divisor=**12** alpha=**0.1**;

estimate "DrinkOsM hyper" TimeBin*Treat*DrinkOsM **174** **0** **0** **0** **174** **0** **0** **0** **174** **0** **0** **0**/cl divisor=**3** alpha=**0.1**;

estimate "DrinkOsM hypo" TimeBin*Treat*DrinkOsM **0** **126.6** **0** **0** **0** **126.6** **0** **0** **0** **126.6** **0** **0**/cl divisor=**3** alpha=**0.1**;

estimate "DrinkOsM iso" TimeBin*Treat*DrinkOsM **0** **0** **17** **0** **0** **0** **17** **0** **0** **0** **17** **0**/cl divisor=**3** alpha=**0.1**;

estimate "DrinkOsM water" TimeBin*Treat*DrinkOsM **0** **0** **0** **0** **0** **0** **0** **0** **0** **0** **0** **0**/cl divisor=**3** alpha=**0.1**;

estimate "Timebin1. ";

estimate "DrinkOsM all" TimeBin*Treat*DrinkOsM **174** **126.6** **17** **0** **0** **0** **0** **0** **0** **0** **0** **0**/cl divisor=**4** alpha=**0.1**;

estimate "DrinkOsM hypo" TimeBin*Treat*DrinkOsM **0** **126.6** **0** **0** **0** **0** **0** **0** **0** **0** **0**/cl divisor=**1** alpha=**0.1**;

estimate "DrinkOsM iso" TimeBin*Treat*DrinkOsM **0** **0** **17** **0** **0** **0** **0** **0** **0** **0** **0** **0** **0**/cl divisor=**1** alpha=**0.1**;

estimate "DrinkOsM water" TimeBin*Treat*DrinkOsM **0** **0** **0** **0** **0** **0** **0** **0** **0** **0** **0** **0**/cl divisor=**1** alpha=**0.1**;

estimate "Timebin2. ";

estimate "DrinkOsM all" TimeBin*Treat*DrinkOsM **0** **0** **0** **0** **174** **126.6** **17** **0** **0** **0** **0** **0**/cl divisor=**4** alpha=**0.1**;

estimate "DrinkOsM hyper" TimeBin*Treat*DrinkOsM **0** **0** **0** **0** **174** **0** **0** **0** **0** **0** **0** **0**/cl divisor=**1** alpha=**0.1**;

estimate "DrinkOsM hypo" TimeBin*Treat*DrinkOsM **0** **0** **0** **0** **0** **126.6** **0** **0** **0** **0** **0** **0**/cl divisor=**1** alpha=**0.1**;

estimate "DrinkOsM iso" TimeBin*Treat*DrinkOsM **0** **0** **0** **0** **0** **0** **17** **0** **0** **0** **0** **0**/cl divisor=**1** alpha=**0.1**;

estimate "DrinkOsM water" TimeBin*Treat*DrinkOsM **0** **0** **0** **0** **0** **0** **0** **0** **0** **0** **0** **0**/cl divisor=**1** alpha=**0.1**;

estimate "Timebin3. ";

estimate "DrinkOsM all" TimeBin*Treat*DrinkOsM **0** **0** **0** **0** **0** **0** **0** **0** **174** **126.6** **17** **0**/cl divisor=**4** alpha=**0.1**;

estimate "DrinkOsM hyper" TimeBin*Treat*DrinkOsM **0** **0** **0** **0** **0** **0** **0** **0** **174** **0** **0** **0**/cl divisor=**1** alpha=**0.1**;

estimate "DrinkOsM hypo" TimeBin*Treat*DrinkOsM **0** **0** **0** **0** **0** **0** **0** **0** **0** **126.6** **0** **0**/cl divisor=**1** alpha=**0.1**;

estimate "DrinkOsM iso" TimeBin*Treat*DrinkOsM **0** **0** **0** **0** **0** **0** **0** **0** **0** **0** **17** **0**/cl divisor=**1** alpha=**0.1**;

estimate "DrinkOsM water" TimeBin*Treat*DrinkOsM **0** **0** **0** **0** **0** **0** **0** **0** **0** **0** **0** **0**/cl divisor=**1** alpha=**0.1**;

estimate " ";

estimate "Effects of 2SD or other of Covariates";

estimate "Metabolicrate all" TimeBin*Treat*metabolicrate **1** **1** **1** **1** **1** **1** **1** **1** **1** **1** **1** **1**/cl divisor=**12** alpha=**0.1**;

estimate "Metabolicrate hyper" TimeBin*Treat*metabolicrate **1** **0** **0** **0** **1** **0** **0** **0** **1** **0** **0** **0**/cl divisor=**3** alpha=**0.1**;

estimate "Metabolicrate hypo" TimeBin*Treat*metabolicrate **0** **1** **0** **0** **0** **1** **0** **0** **0** **1** **0** **0**/cl divisor=**3** alpha=**0.1**;

estimate "Metabolicrate iso" TimeBin*Treat*metabolicrate **0** **0** **1** **0** **0** **0** **1** **0** **0** **0** **1** **0**/cl divisor=**3** alpha=**0.1**;

estimate "Metabolicrate water" TimeBin*Treat*metabolicrate **0** **0** **0** **1** **0** **0** **0** **1** **0** **0** **0** **1**/cl divisor=**3** alpha=**0.1**;

estimate "Timebin1. ";

estimate "Metabolicrate all" TimeBin*Treat*metabolicrate **1** **1** **1** **1** **0** **0** **0** **0** **0** **0** **0** **0**/cl divisor=**4** alpha=**0.1**;

estimate "Metabolicrate hyper" TimeBin*Treat*metabolicrate **1** **0** **0** **0** **0** **0** **0** **0** **0** **0** **0** **0**/cl divisor=**1** alpha=**0.1**;

estimate "Metabolicrate hypo" TimeBin*Treat*metabolicrate **0** **1** **0** **0** **0** **0** **0** **0** **0** **0** **0**/cl divisor=**1** alpha=**0.1**;

estimate "Metabolicrate iso" TimeBin*Treat*metabolicrate **0** **0** **1** **0** **0** **0** **0** **0** **0** **0** **0** **0** **0**/cl divisor=**1** alpha=**0.1**;

estimate "Metabolicrate water" TimeBin*Treat*metabolicrate **0** **0** **0** **1** **0** **0** **0** **0** **0** **0** **0** **0**/cl divisor=**1** alpha=**0.1**;

estimate "Timebin2. ";

estimate "Metabolicrate all" TimeBin*Treat*metabolicrate **0** **0** **0** **0** **1** **1** **1** **1** **0** **0** **0** **0**/cl divisor=**4** alpha=**0.1**;

estimate "Metabolicrate hyper" TimeBin*Treat*metabolicrate **0** **0** **0** **0** **1** **0** **0** **0** **0** **0** **0** **0**/cl divisor=**1** alpha=**0.1**;

estimate "Metabolicrate hypo" TimeBin*Treat*metabolicrate **0** **0** **0** **0** **0** **1** **0** **0** **0** **0** **0** **0**/cl divisor=**1** alpha=**0.1**;

estimate "Metabolicrate iso" TimeBin*Treat*metabolicrate **0** **0** **0** **0** **0** **0** **1** **0** **0** **0** **0** **0**/cl divisor=**1** alpha=**0.1**;

estimate "Metabolicrate water" TimeBin*Treat*metabolicrate **0** **0** **0** **0** **0** **0** **0** **1** **0** **0** **0** **0**/cl divisor=**1** alpha=**0.1**;

estimate "Timebin3. ";

estimate "Metabolicrate all" TimeBin*Treat*metabolicrate **0** **0** **0** **0** **0** **0** **0** **0** **1** **1** **1** **1**/cl divisor=**4** alpha=**0.1**;

estimate "Metabolicrate hyper" TimeBin*Treat*metabolicrate **0** **0** **0** **0** **0** **0** **0** **0** **1** **0** **0** **0**/cl divisor=**1** alpha=**0.1**;

estimate "Metabolicrate hypo" TimeBin*Treat*metabolicrate **0** **0** **0** **0** **0** **0** **0** **0** **0** **1** **0** **0**/cl divisor=**1** alpha=**0.1**;

estimate "Metabolicrate iso" TimeBin*Treat*metabolicrate **0** **0** **0** **0** **0** **0** **0** **0** **0** **0** **1** **0**/cl divisor=**1** alpha=**0.1**;

estimate "Metabolicrate water" TimeBin*Treat*metabolicrate **0** **0** **0** **0** **0** **0** **0** **0** **0** **0** **0** **1**/cl divisor=**1** alpha=**0.1**;

/*

estimate "heatindex all" TimeBin*Treat*heatindex 1 1 1 1 1 1 1 1 1 1 1 1/cl divisor=12 alpha=0.1;

estimate "heatindex hyper" TimeBin*Treat*heatindex 1 0 0 0 1 0 0 0 1 0 0 0/cl divisor=3 alpha=0.1;

estimate "heatindex hypo" TimeBin*Treat*heatindex 0 1 0 0 0 1 0 0 0 1 0 0/cl divisor=3 alpha=0.1;

estimate "heatindex iso" TimeBin*Treat*heatindex 0 0 1 0 0 0 1 0 0 0 1 0/cl divisor=3 alpha=0.1;

estimate "heatindex water" TimeBin*Treat*heatindex 0 0 0 1 0 0 0 1 0 0 0 1/cl divisor=3 alpha=0.1;

*/

estimate " ";

estimate "Effects of 2SD or other of Covariates";

estimate "AvgIngestionRate all" TimeBin*Treat*avg_ingestion_rate **1** **1** **1** **1** **1** **1** **1** **1** **1** **1** **1** **1**/cl divisor=**12** alpha=**0.1**;

estimate "AvgIngestionRate hyper" TimeBin*Treat*avg_ingestion_rate **1** **0** **0** **0** **1** **0** **0** **0** **1** **0** **0** **0**/cl divisor=**3** alpha=**0.1**;

estimate "AvgIngestionRate hypo" TimeBin*Treat*avg_ingestion_rate **0** **1** **0** **0** **0** **1** **0** **0** **0** **1** **0** **0**/cl divisor=**3** alpha=**0.1**;

estimate "AvgIngestionRate iso" TimeBin*Treat*avg_ingestion_rate **0** **0** **1** **0** **0** **0** **1** **0** **0** **0** **1** **0**/cl divisor=**3** alpha=**0.1**;

estimate "AvgIngestionRate water" TimeBin*Treat*avg_ingestion_rate **0** **0** **0** **1** **0** **0** **0** **1** **0** **0** **0** **1**/cl divisor=**3** alpha=**0.1**;

estimate "Timebin1. ";

estimate "AvgIngestionRate all" TimeBin*Treat*avg_ingestion_rate **1** **1** **1** **1** **0** **0** **0** **0** **0** **0** **0** **0**/cl divisor=**4** alpha=**0.1**;

estimate "AvgIngestionRate hyper" TimeBin*Treat*avg_ingestion_rate **1** **0** **0** **0** **0** **0** **0** **0** **0** **0** **0** **0**/cl divisor=**1** alpha=**0.1**;

estimate "AvgIngestionRate hypo" TimeBin*Treat*avg_ingestion_rate **0** **1** **0** **0** **0** **0** **0** **0** **0** **0** **0**/cl divisor=**1** alpha=**0.1**;

estimate "AvgIngestionRate iso" TimeBin*Treat*avg_ingestion_rate **0** **0** **1** **0** **0** **0** **0** **0** **0** **0** **0** **0** **0**/cl divisor=**1** alpha=**0.1**;

estimate "AvgIngestionRate water" TimeBin*Treat*avg_ingestion_rate **0** **0** **0** **1** **0** **0** **0** **0** **0** **0** **0** **0**/cl divisor=**1** alpha=**0.1**;

estimate "Timebin2. ";

estimate "AvgIngestionRate all" TimeBin*Treat*avg_ingestion_rate **0** **0** **0** **0** **1** **1** **1** **1** **0** **0** **0** **0**/cl divisor=**4** alpha=**0.1**;

estimate "AvgIngestionRate hyper" TimeBin*Treat*avg_ingestion_rate **0** **0** **0** **0** **1** **0** **0** **0** **0** **0** **0** **0**/cl divisor=**1** alpha=**0.1**;

estimate "AvgIngestionRate hypo" TimeBin*Treat*avg_ingestion_rate **0** **0** **0** **0** **0** **1** **0** **0** **0** **0** **0** **0**/cl divisor=**1** alpha=**0.1**;

estimate "AvgIngestionRate iso" TimeBin*Treat*avg_ingestion_rate **0** **0** **0** **0** **0** **0** **1** **0** **0** **0** **0** **0**/cl divisor=**1** alpha=**0.1**;

estimate "AvgIngestionRate water" TimeBin*Treat*avg_ingestion_rate **0** **0** **0** **0** **0** **0** **0** **1** **0** **0** **0** **0**/cl divisor=**1** alpha=**0.1**;

estimate "Timebin3. ";

estimate "AvgIngestionRate all" TimeBin*Treat*avg_ingestion_rate **0** **0** **0** **0** **0** **0** **0** **0** **1** **1** **1** **1**/cl divisor=**4** alpha=**0.1**;

estimate "AvgIngestionRate hyper" TimeBin*Treat*avg_ingestion_rate **0** **0** **0** **0** **0** **0** **0** **0** **1** **0** **0** **0**/cl divisor=**1** alpha=**0.1**;

estimate "AvgIngestionRate hypo" TimeBin*Treat*avg_ingestion_rate **0** **0** **0** **0** **0** **0** **0** **0** **0** **1** **0** **0**/cl divisor=**1** alpha=**0.1**;

estimate "AvgIngestionRate iso" TimeBin*Treat*avg_ingestion_rate **0** **0** **0** **0** **0** **0** **0** **0** **0** **0** **1** **0**/cl divisor=**1** alpha=**0.1**;

estimate "AvgIngestionRate water" TimeBin*Treat*avg_ingestion_rate **0** **0** **0** **0** **0** **0** **0** **0** **0** **0** **0** **1**/cl divisor=**1** alpha=**0.1**;

****;

estimate " ";

estimate "DrinkOsm V.likely d Analysis";

estimate "TimeBinMeansOverall mean setting";*automatically adjusted for standardised covariates. If not standardised then need to put in the covariate;

estimate "Hyper" TimeBin*Treat **1** **0** **0** **0** **1** **0** **0** **0** **1** **0** **0** **0** TimeBin*Treat*DrinkOsM **95.8** **0** **0** **0** **95.8** **0** **0** **0** **95.8** **0** **0** **0**/cl divisor=**3** alpha=**0.1**;

estimate "Hypo" TimeBin*Treat **0** **1** **0** **0** **0** **1** **0** **0** **0** **1** **0** **0** TimeBin*Treat*DrinkOsM **0** -**109.1** **0** **0** **0** -**109.1** **0** **0** **0** -**109.1** **0** **0**/cl divisor=**3** alpha=**0.1**;

estimate "Iso" TimeBin*Treat **0** **0** **1** **0** **0** **0** **1** **0** **0** **0** **1** **0** TimeBin*Treat*DrinkOsM **0** **0** -**2.0** **0** **0** **0** -**2.0** **0** **0** **0** -**2.0** **0**/cl divisor=**3** alpha=**0.1**;

estimate "Water" TimeBin*Treat **0** **0** **0** **1** **0** **0** **0** **1** **0** **0** **0** **1** /cl divisor=**3** alpha=**0.1**;

estimate "Hyper-iso" TimeBin*Treat **1** **0** -**1** **0** **1** **0** -**1** **0** **1** **0** -**1** **0** TimeBin*Treat*DrinkOsM **95.8** **0** **2.0** **0** **95.8** **0** **2.0** **0** **95.8** **0** **2.0** **0**/cl divisor=**3** alpha=**0.1**;

estimate "Hypo-iso" TimeBin*Treat **0** **1** -**1** **0** **0** **1** -**1** **0** **0** **1** -**1** **0** TimeBin*Treat*DrinkOsM **0** -**109.1** **2.0** **0** **0** -**109.1** **2.0** **0** **0** -**109.1** **2.0** **0**/cl divisor=**3** alpha=**0.1**;

estimate "Water-iso" TimeBin*Treat **0** **0** -**1** **1** **0** **0** -**1** **1** **0** **0** -**1** **1** TimeBin*Treat*DrinkOsM **0** **0** **2.0** **0** **0** **0** **2.0** **0** **0** **0** **2.0** **0**/cl divisor=**3** alpha=**0.1**;

estimate "Hyper-Hypo" TimeBin*Treat **1** -**1** **0** **0** **1** -**1** **0** **0** **1** -**1** **0** **0** TimeBin*Treat*DrinkOsM **95.8** **109.1** **0** **0** **95.8** **109.1** **0** **0** **95.8** **103.1** **0** **0**/cl divisor=**3** alpha=**0.1**; *check iso is a postivie then ave covar is a -103.1;

estimate "Hyper-Water" TimeBin*Treat **1** **0** **0** -**1** **1** **0** **0** -**1** **1** **0** **0** -**1** TimeBin*Treat*DrinkOsM **95.8** **0** **0** **0** **95.8** **0** **0** **0** **95.8** **0** **0** **0**/cl divisor=**3** alpha=**0.1**;

estimate "Hypo-Water" TimeBin*Treat **0** **1** **0** -**1** **0** **1** **0** -**1** **0** **1** **0** -**1** TimeBin*Treat*DrinkOsM **0** -**109.1** **0** **0** **0** -**109.1** **0** **0** **0** -**109.1** **0** **0**/cl divisor=**3** alpha=**0.1**;

estimate "TimeBinMeansOverall indiv setting";*automatically adjusted for standardised covariates. If not standardised then need to put in the covariate;

estimate "Hyper" TimeBin*Treat **1** **0** **0** **0** **1** **0** **0** **0** **1** **0** **0** **0** TimeBin*Treat*DrinkOsM **95.8** **0** **0** **0** **95.8** **0** **0** **0** **95.8** **0** **0** **0**|StudyID **1** StudyID*ExptID **1** StudyID*EstimateID **1**/cl divisor=**3** alpha=**0.1**;

estimate "Hypo" TimeBin*Treat **0** **1** **0** **0** **0** **1** **0** **0** **0** **1** **0** **0** TimeBin*Treat*DrinkOsM **0** -**109.1** **0** **0** **0** -**109.1** **0** **0** **0** -**109.1** **0** **0**|StudyID **1** StudyID*ExptID **1** StudyID*EstimateID **1**/cl divisor=**3** alpha=**0.1**;

estimate "Iso" TimeBin*Treat **0** **0** **1** **0** **0** **0** **1** **0** **0** **0** **1** **0** TimeBin*Treat*DrinkOsM **0** **0** -**2.0** **0** **0** **0** -**2.0** **0** **0** **0** -**2.0** **0**|StudyID **1** StudyID*ExptID **1** StudyID*EstimateID **1**/cl divisor=**3** alpha=**0.1**;

estimate "Water" TimeBin*Treat **0** **0** **0** **1** **0** **0** **0** **1** **0** **0** **0** **1** |StudyID **1** StudyID*ExptID **1** StudyID*EstimateID **1**/cl divisor=**3** alpha=**0.1**;

estimate "Hyper-iso" TimeBin*Treat **1** **0** -**1** **0** **1** **0** -**1** **0** **1** **0** -**1** **0** TimeBin*Treat*DrinkOsM **95.8** **0** **2.0** **0** **95.8** **0** **2.0** **0** **95.8** **0** **2.0** **0**|StudyID **1** StudyID*ExptID **1** StudyID*EstimateID **1**/cl divisor=**3** alpha=**0.1**;

estimate "Hypo-iso" TimeBin*Treat **0** **1** -**1** **0** **0** **1** -**1** **0** **0** **1** -**1** **0** TimeBin*Treat*DrinkOsM **0** -**109.1** **2.0** **0** **0** -**109.1** **2.0** **0** **0** -**109.1** **2.0** **0**|StudyID **1** StudyID*ExptID **1** StudyID*EstimateID **1**/cl divisor=**3** alpha=**0.1**;

estimate "Water-iso" TimeBin*Treat **0** **0** -**1** **1** **0** **0** -**1** **1** **0** **0** -**1** **1** TimeBin*Treat*DrinkOsM **0** **0** **2.0** **0** **0** **0** **2.0** **0** **0** **0** **2.0** **0**|StudyID **1** StudyID*ExptID **1** StudyID*EstimateID **1**/cl divisor=**3** alpha=**0.1**;

estimate "Hyper-Hypo" TimeBin*Treat **1** -**1** **0** **0** **1** -**1** **0** **0** **1** -**1** **0** **0** TimeBin*Treat*DrinkOsM **95.8** **109.1** **0** **0** **95.8** **109.1** **0** **0** **95.8** **103.1** **0** **0**|StudyID **1** StudyID*ExptID **1** StudyID*EstimateID **1**/cl divisor=**3** alpha=**0.1**; *check iso is a postivie then ave covar is a -103.1;

estimate "Hyper-Water" TimeBin*Treat **1** **0** **0** -**1** **1** **0** **0** -**1** **1** **0** **0** -**1** TimeBin*Treat*DrinkOsM **95.8** **0** **0** **0** **95.8** **0** **0** **0** **95.8** **0** **0** **0**|StudyID **1** StudyID*ExptID **1** StudyID*EstimateID **1**/cl divisor=**3** alpha=**0.1**;

estimate "Hypo-Water" TimeBin*Treat **0** **1** **0** -**1** **0** **1** **0** -**1** **0** **1** **0** -**1** TimeBin*Treat*DrinkOsM **0** -**109.1** **0** **0** **0** -**109.1** **0** **0** **0** -**109.1** **0** **0**|StudyID **1** StudyID*ExptID **1** StudyID*EstimateID **1**/cl divisor=**3** alpha=**0.1**;

estimate " ";

estimate "DrinkOsm V.likely d Analysis";

estimate "TimeBin1.<31 mean setting";

estimate "Hyper" TimeBin*Treat **1** **0** **0** **0** **0** **0** **0** **0** **0** **0** **0** **0** TimeBin*Treat*DrinkOsM **95.8** **0** **0** **0** **0** **0** **0** **0** **0** **0** **0** **0**/cl divisor=**1** alpha=**0.1**;

estimate "Hypo" TimeBin*Treat **0** **1** **0** **0** **0** **0** **0** **0** **0** **0** **0** **0** TimeBin*Treat*DrinkOsM **0** -**109.1** **0** **0** **0** **0** **0** **0** **0** **0** **0** **0**/cl divisor=**1** alpha=**0.1**;

estimate "Iso" TimeBin*Treat **0** **0** **1** **0** **0** **0** **0** **0** **0** **0** **0** **0** TimeBin*Treat*DrinkOsM **0** **0** -**2.0** **0** **0** **0** **0** **0** **0** **0** **0** **0**/cl divisor=**1** alpha=**0.1**;

estimate "Water" TimeBin*Treat **0** **0** **0** **1** **0** **0** **0** **0** **0** **0** **0** **0** /cl divisor=**1** alpha=**0.1**;

estimate "Hyper-iso" TimeBin*Treat **1** **0** -**1** **0** **0** **0** **0** **0** **0** **0** **0** **0** TimeBin*Treat*DrinkOsM **95.8** **0** **2.0** **0** **0** **0** **0** **0** **0** **0** **0** **0**/cl divisor=**1** alpha=**0.1**;

estimate "Hypo-iso" TimeBin*Treat **0** **1** -**1** **0** **0** **0** **0** **0** **0** **0** **0** **0** TimeBin*Treat*DrinkOsM **0** -**109.1** **2.0** **0** **0** **0** **0** **0** **0** **0** **0** **0**/cl divisor=**1** alpha=**0.1**;

estimate "Water-iso" TimeBin*Treat **0** **0** -**1** **1** **0** **0** **0** **0** **0** **0** **0** **0** TimeBin*Treat*DrinkOsM **0** **0** **2.0** **0** **0** **0** **0** **0** **0** **0** **0** **0**/cl divisor=**1** alpha=**0.1**;

estimate "Hyper-Hypo" TimeBin*Treat **1** -**1** **0** **0** **0** **0** **0** **0** **0** **0** **0** **0** TimeBin*Treat*DrinkOsM **95.8** **109.1** **0** **0** **0** **0** **0** **0** **0** **0** **0** **0**/cl divisor=**1** alpha=**0.1**; *check iso is a postivie then ave covar is a -103.1;

estimate "Hyper-Water" TimeBin*Treat **1** **0** **0** -**1** **0** **0** **0** **0** **0** **0** **0** **0** TimeBin*Treat*DrinkOsM **95.8** **0** **0** **0** **0** **0** **0** **0** **0** **0** **0** **0**/cl divisor=**1** alpha=**0.1**;

estimate "Hypo-Water" TimeBin*Treat **0** **1** **0** -**1** **0** **0** **0** **0** **0** **0** **0** **0** TimeBin*Treat*DrinkOsM **0** -**109.1** **0** **0** **0** **0** **0** **0** **0** **0** **0** **0**/cl divisor=**1** alpha=**0.1**;

estimate "TimeBin2.31-60 mean setting";

estimate "Hyper" TimeBin*Treat **0** **0** **0** **0** **1** **0** **0** **0** **0** **0** **0** **0** TimeBin*Treat*DrinkOsM **0** **0** **0** **0** **95.8** **0** **0** **0** **0** **0** **0** **0**/cl divisor=**1** alpha=**0.1**;

estimate "Hypo" TimeBin*Treat **0** **0** **0** **0** **0** **1** **0** **0** **0** **0** **0** **0** TimeBin*Treat*DrinkOsM **0** **0** **0** **0** **0** -**109.1** **0** **0** **0** **0** **0** **0**/cl divisor=**1** alpha=**0.1**;

estimate "Iso" TimeBin*Treat **0** **0** **0** **0** **0** **0** **1** **0** **0** **0** **0** **0** TimeBin*Treat*DrinkOsM **0** **0** **0** **0** **0** **0** -**2.0** **0** **0** **0** **0** **0**/cl divisor=**1** alpha=**0.1**;

estimate "Water" TimeBin*Treat **0** **0** **0** **0** **0** **0** **0** **1** **0** **0** **0** **0** /cl divisor=**1** alpha=**0.1**;

estimate "Hyper-iso" TimeBin*Treat **0** **0** **0** **0** **1** **0** -**1** **0** **0** **0** **0** **0** TimeBin*Treat*DrinkOsM **0** **0** **0** **0** **95.8** **0** **2.0** **0** **0** **0** **0** **0**/cl divisor=**1** alpha=**0.1**;

estimate "Hypo-iso" TimeBin*Treat **0** **0** **0** **0** **0** **1** -**1** **0** **0** **0** **0** **0** TimeBin*Treat*DrinkOsM **0** **0** **0** **0** **0** -**109.1** **2.0** **0** **0** **0** **0** **0**/cl divisor=**1** alpha=**0.1**;

estimate "Water-iso" TimeBin*Treat **0** **0** **0** **0** **0** **0** -**1** **1** **0** **0** **0** **0** TimeBin*Treat*DrinkOsM **0** **0** **0** **0** **0** **0** **2.0** **0** **0** **0** **0** **0**/cl divisor=**1** alpha=**0.1**;

estimate "Hyper-Hypo" TimeBin*Treat **0** **0** **0** **0** **1** -**1** **0** **0** **0** **0** **0** **0** TimeBin*Treat*DrinkOsM **0** **0** **0** **0** **95.8** **109.1** **0** **0** **0** **0** **0** **0**/cl divisor=**1** alpha=**0.1**; *check iso is a postivie then ave covar is a -103.1;

estimate "Hyper-Water" TimeBin*Treat **0** **0** **0** **0** **1** **0** **0** -**1** **0** **0** **0** **0** TimeBin*Treat*DrinkOsM **0** **0** **0** **0** **95.8** **0** **0** **0** **0** **0** **0** **0**/cl divisor=**1** alpha=**0.1**;

estimate "Hypo-Water" TimeBin*Treat **0** **0** **0** **0** **0** **1** **0** -**1** **0** **0** **0** **0** TimeBin*Treat*DrinkOsM **0** **0** **0** **0** **0** -**109.1** **0** **0** **0** **0** **0** **0**/cl divisor=**1** alpha=**0.1**;

estimate "TimeBin3.60-180 mean setting";

estimate "Hyper" TimeBin*Treat **0** **0** **0** **0** **0** **0** **0** **0** **1** **0** **0** **0** TimeBin*Treat*DrinkOsM **0** **0** **0** **0** **0** **0** **0** **0** **95.8** **0** **0** **0**/cl divisor=**1** alpha=**0.1**;

estimate "Hypo" TimeBin*Treat **0** **0** **0** **0** **0** **0** **0** **0** **0** **1** **0** **0** TimeBin*Treat*DrinkOsM **0** **0** **0** **0** **0** **0** **0** **0** **0** -**109.1** **0** **0**/cl divisor=**1** alpha=**0.1**;

estimate "Iso" TimeBin*Treat **0** **0** **0** **0** **0** **0** **0** **0** **0** **0** **1** **0** TimeBin*Treat*DrinkOsM **0** **0** **0** **0** **0** **0** **0** **0** **0** **0** -**2.0** **0**/cl divisor=**1** alpha=**0.1**;

estimate "Water" TimeBin*Treat **0** **0** **0** **0** **0** **0** **0** **0** **0** **0** **0** **1** /cl divisor=**1** alpha=**0.1**;

estimate "Hyper-iso" TimeBin*Treat **0** **0** **0** **0** **0** **0** **0** **0** **1** **0** -**1** **0** TimeBin*Treat*DrinkOsM **0** **0** **0** **0** **0** **0** **0** **0** **95.8** **0** **2.0** **0**/cl divisor=**1** alpha=**0.1**;

estimate "Hypo-iso" TimeBin*Treat **0** **0** **0** **0** **0** **0** **0** **0** **0** **1** -**1** **0** TimeBin*Treat*DrinkOsM **0** **0** **0** **0** **0** **0** **0** **0** **0** -**109.1** **2.0** **0**/cl divisor=**1** alpha=**0.1**;

estimate "Water-iso" TimeBin*Treat **0** **0** **0** **0** **0** **0** **0** **0** **0** **0** -**1** **1** TimeBin*Treat*DrinkOsM **0** **0** **0** **0** **0** **0** **0** **0** **0** **0** **2.0** **0**/cl divisor=**1** alpha=**0.1**;

estimate "Hyper-Hypo" TimeBin*Treat **0** **0** **0** **0** **0** **0** **0** **0** **1** -**1** **0** **0** TimeBin*Treat*DrinkOsM **0** **0** **0** **0** **0** **0** **0** **0** **95.8** **103.1** **0** **0**/cl divisor=**1** alpha=**0.1**; *check iso is a postivie then ave covar is a -103.1;

estimate "Hyper-Water" TimeBin*Treat **0** **0** **0** **0** **0** **0** **0** **0** **1** **0** **0** -**1** TimeBin*Treat*DrinkOsM **0** **0** **0** **0** **0** **0** **0** **0** **95.8** **0** **0** **0**/cl divisor=**1** alpha=**0.1**;

estimate "Hypo-Water" TimeBin*Treat **0** **0** **0** **0** **0** **0** **0** **0** **0** **1** **0** -**1** TimeBin*Treat*DrinkOsM **0** **0** **0** **0** **0** **0** **0** **0** **0** -**109.1** **0** **0**/cl divisor=**1** alpha=**0.1**;

ods output covparms=cov;

ods output residualsdPV=residualsdPV;

ods output lsmeans=lsm;

*ods output diffs=lsmdif;

ods output estimates=est;

ods output solutionf=solf;

**run**;

ods listing;

**data** covbtwn;

set cov;

SE2=stderr****2**;

if covparm ne "Residual";

**proc** **means** noprint;

var estimate se2;

output out=covbtwn1 sum=;

**data** covbtwn2;

covparm="Btwn settings";

set covbtwn1;

StdErr=sqrt(se2);

*alpha=&alpha;

lower=estimate+probit(alpha/**2**)*StdErr;*probit not working;

upper=estimate-probit(alpha/**2**)*StdErr;

Zvalue=estimate/StdErr;

**run**;

**data** cov1;

set cov covbtwn2;

if covparm ne "Residual";

DegFree=**2***Zvalue****2**;

a=**1**;b=**1**;c=**1**;

if estimate<**0** then a=-**1**;

if lower<**0** then b=-**1**;

if upper<**0** then c=-**1**;

SD=a*sqrt(a*estimate);

lower=b*sqrt(b*lower);

upper=c*sqrt(c*upper);

array r SD lower upper;

*if &logflag=1 then do over r;

*r=100*exp(r/100)-100;

*end;

*CLtd=sqrt(upper/lower);

CLpm=(upper-lower)/**2**;

Units="Raw";

*if &logflag then Units="%";

title2 "Random effects as SD";

options ls=**100**;

**proc** **print** data=cov1 noobs;

var covparm Units SD CLpm lower upper alpha DegFree;

format SD lower upper CLpm **5.2** DegFree **5.**;

**run**;

title2 "MBI for random effects";

**data** cov1;

set cov covbtwn2;

if covparm ne "Residual";

DF=**999**;

*MagniThresh=abs(&MagniThresh)/2;

MagniThresh=abs(**0.2*****2.62**)/**2**;*0.2 times baseline SD for dPV as a percent of mean;

*if &logflag then Magnithresh=100*exp(log(1+abs(&magnithresh)/100)/2)-100;

ProbPos=(**1**-ProbT(-(estimate-abs(MagniThresh))/StdErr,DF));

ProbNeg=ProbT(-(estimate+abs(MagniThresh))/StdErr,DF);

*if &LogFlag=1 then do;

if Magnithresh>**0** then do;

ProbPos=(**1**-ProbT(-(estimate-(MagniThresh))/StdErr,DF));

ProbNeg=ProbT(-(estimate+(MagniThresh))/StdErr,DF);

end;

else do;

ProbPos=(**1**-ProbT(-(estimate+(MagniThresh))/StdErr,DF));

ProbNeg=ProbT(-(estimate-(MagniThresh))/StdErr,DF);

end;

*end;

ProbTriv=**1**-ProbPos-ProbNeg;

ORPosNeg=ProfPos/(**1**-ProbPos)/(ProbNeg/(**1**-ProbNeg));

ORNegPos=**1**/ORPosNeg;

ClinFlag=**1**; *want all inferences to be clinical initially;

*if index(label,"2SD") then ClinFlag=0; *covariates definitely need to be non-clinical;

*mechanistic inferences as frequentist;

*ClinFlag=0;

ClearOrNot="unclear";

Strgth="";

Magni="";

ORPosNeg=**.**; ORNegPos=**.**;

if ProbNeg<**0.05** or ProbPos<**0.05** then ClearOrNot="@0.1% ";

if ProbNeg<**0.005** or ProbPos<**0.005** then ClearOrNot="@0.01% ";

if ClearOrNot ne "unclear" then do;

Magni="+ive ";

if estimate<**0** then Magni="-ive ";

if ProbPos>**0.05** or ProbNeg>**0.05** then Strgth="improbable";

if ProbPos>**0.25** or ProbNeg>**0.25** then Strgth="about as likely as not";

if ProbPos>**0.75** or ProbNeg>**0.75** then Strgth="likely ";

if ProbPos>**0.95** or ProbNeg>**0.95** then Strgth="v.likely ";

if ProbPos>**0.995** or ProbNeg>**0.995** then Strgth="virtually_certain";

end;

if ClearOrNot ne "unclear" and ProbTriv>**75** then do;

Magni="triv.";

Strgth="weak ";

if ProbTriv>**0.95** then Prob="v.likely ";

if ProbTriv>**0.995** then Prob="virtually_certain";

end;

*end;

output;

**data** cov2;

set cov1;

a=**1**;b=**1**;c=**1**;

if estimate<**0** then a=-**1**;

if lower<**0** then b=-**1**;

if upper<**0** then c=-**1**;

SD=a*sqrt(a*estimate);

lower=b*sqrt(b*lower);

upper=c*sqrt(c*upper);

DegFree=**2***Zvalue****2**;

array r SD lower upper;

/*

if &logflag=1 then do over r;

r=100*exp(r/100)-100;

end;

*/

*CLtd=sqrt(upper/lower);

CLpm=(upper-lower)/**2**;

Units="Raw";

*if &logflag then Units="%";

**run**;

options ls=**120** ps=**80**;

**proc** **print** data=cov2 noobs;

*where clinflag=0;

var covparm Units SD CLpm lower upper alpha

MagniThresh ProbNeg ProbTriv ProbPos Strgth Magni ClearOrNot;

format SD CLpm lower upper MagniThresh **5.2** Probt best5. ProbNeg ProbTriv ProbPos **5.1** DegFree **5.0**;

title3 "Non-clinical inferences for SD representing random effects";

*title4 "threshold is half the smallest difference for means";

**run**;

********************;

**data** est1;

set est;

*Units="Raw dPV";

CLpm=(Upper+Lower)/**2**;/*

*if &logflag then Units="factor";

array a estimate lower upper;

if &logflag then do over a;

a=exp(a/100); *all are factors;

end;

CLtd=sqrt(Upper/Lower);

if estimate=1 then do; estimate=.; Units=""; end;*/

**run**;

/*

title2 "Fixed-effect estimates";

proc print noobs data=est1;

var Label Estimate CLpm Lower Upper alpha;

format Estimate Lower Upper CLpm 5.2;

run;

*/

*Decisions for fixed effects;

*clin and non-clin MBIs are output and listed separately;

/* The thresholds for small, v.likely , large, very large and extremely large standardised mean differences (SMD) were 1, 3, 6, 10 and 20 times the SIE threshold, respectively (corresponding to 0.2, 0.6, 1.2, 2.0 and 4.0 times the SD) [1]. The thresholds were also used to evaluate the magnitude of the numeric linear modifier where done by multiplying the beta-coefficients (slopes) in the model by two between-subject SD and between-study SD, respectively. The thresholds were halved for evaluation of the magnitude of the random-effect SDs [2].

1. Cassar S, et al Human Rep. 2016;31:2619-31.

2. Smith TB and Hopkins WG. Med Sci Sports Exerc. 2011;43:2155-60.

*/

title3 "Decisions for fixed effects";

**data** est2;

set est1;

length Strgth $ **10** Magni $ **4**;

MagniThresh=**0.2*****2.62**; *%SD baseline PV from young mean 2.03% Davy, K. P., & Seals, D. R. (1994). Total blood volume in healthy young and older men. J Appl Physiol (1985), 76(5), 2059-2062. doi: 10.1152/jappl.1994.76.5.2059

age 40-49 yr sample 3.2% from Yiengst, M. J., & Shock, N. W. (1962). Blood and plasma volume in adult males. J Appl Physiol, 17, 195-198. doi: 10.1152/jappl.1962.17.2.195;

/*

MagniThresh=0.2*2.6135; *2.6135

Hyper 2.68787

Hypo 3.07517

Iso 3.04675

Water 1.16584;

*if _n_=1 then set covarSD(keep=stddev);

*/

*SD=stddev;

Cn=**1**-**3**/(**4***df-**1**); *bias is due to uncertainty in SD;

ES=Cn*estimate/(MagniThresh/**0.2**);*SD;

*NCparm=tValue*sqrt((DFdenom+1)/(DFnum+1));

*alpha=0.1;

*CL_pc=alpha*100;

ESlower=ES/tValue*TINV(alpha/**2**,df,tvalue);

ESupper=ES/tValue*TINV(**1**-alpha/**2**,df,tvalue);

*Changed from Changes to Frequentist Probabilities by deleting 100*;

ProbPos=(**1**-ProbT(-(estimate-abs(MagniThresh))/StdErr,DF));

ProbNeg=ProbT(-(estimate+abs(MagniThresh))/StdErr,DF);

*if &LogFlag=1 then do;

if Magnithresh>**0** then do;

ProbPos=(**1**-ProbT(-(estimate-(MagniThresh))/StdErr,DF));

ProbNeg=ProbT(-(estimate+(MagniThresh))/StdErr,DF);

end;

else do;

ProbPos=(**1**-ProbT(-(estimate+(MagniThresh))/StdErr,DF));

ProbNeg=ProbT(-(estimate-(MagniThresh))/StdErr,DF);

end;

*end;

ProbTriv=**1**-ProbPos-ProbNeg;

ORPosNeg=ProfPos/(**1**-ProbPos)/(ProbNeg/(**1**-ProbNeg));

ORNegPos=**1**/ORPosNeg;

ClinFlag=**1**; *want all inferences to be clinical initially;

*if index(label,"2SD") then ClinFlag=0; *covariates definitely need to be non-clinical;

/*

*clinical inferences;

if clinflag then do;

ChPos=ProbPos; ChNeg=ProbNeg;

if MagniThresh<0 then do;

ChPos=ProbNeg; ChNeg=ProbPos;

end;

Prob=""; Magni=""; ClearOrNot="unclear";

if ChNeg<0.5 then do;

ClearOrNot="@25/.5%";

if ChNeg<0.1 then ClearOrNot="@5/.1% ";

if ProbTriv>25 then Magni="triv";

if ChPos>25 then Magni="bene";

Prob=“about as likely as not”;

if ChPos>75 or ProbTriv>75 then Prob="likely ";

if ChPos>95 or ProbTriv>95 then Prob="v.likely";

if ChPos>99.5 or ProbTriv>99.5 then Prob=“virtually certain”;

end;

else do; *i.e., ChNeg>0.5;

if ChPos<25 then do;

ClearOrNot="@25/.5%";

if ChPos<5 then ClearOrNot="@5/.1% ";

if ProbTriv>25 then Magni="triv";

if ChNeg>25 then Magni="harm";

Prob=“about as likely as not”;

if ChNeg>75 or ProbTriv>75 then Prob="likely ";

if ChNeg>95 or ProbTriv>95 then Prob="v.likely";

if ChNeg>99.5 or ProbTriv>99.5 then Prob=“virtually certain”;

end;

end;

if ClearOrNot="unclear" and

(MagniThresh>0 and ORPosNeg>25/75/(0.5/99.5) or MagniThresh<0 and ORNegPos>25/75/(0.5/99.5))

then do;

ClearOrNot="OR>66.3";

Magni="bene";

if ChPos>25 then Prob=“about as likely as not”;

if ChPos>75 then Prob="likely ";

if ChPos>95 then Prob="v.likely";

if ChPos>99.5 then Prob=“virtually certain”;

end;

output;

end;

*/

*mechanistic inferences as frequentist;

*ClinFlag=0;

ClearOrNot="unclear";

Strgth="";

Magni="";

ORPosNeg=**.**; ORNegPos=**.**;

if ProbNeg<**0.05** or ProbPos<**0.05** then ClearOrNot="@0.1% ";

if ProbNeg<**0.005** or ProbPos<**0.005** then ClearOrNot="@0.01% ";

if ClearOrNot ne "unclear" then do;

Magni="+ive ";

if estimate<**0** then Magni="-ive ";

if ProbPos>**0.05** or ProbNeg>**0.05** then Strgth="about as likely as not";

if ProbPos>**0.25** or ProbNeg>**0.25** then Strgth="about as likely as not";

if ProbPos>**0.75** or ProbNeg>**0.75** then Strgth="likely ";

if ProbPos>**0.95** or ProbNeg>**0.95** then Strgth="v.likely ";

if ProbPos>**0.995** or ProbNeg>**0.995** then Strgth="virtually_certain";

end;

if ClearOrNot ne "unclear" and ProbTriv>**75** then do;

Magni="triv.";

Strgth="weak ";

if ProbTriv>**0.95** then Prob="v.likely ";

if ProbTriv>**0.995** then Prob="virtually_certain";

end;

*end;

output;

/*

*mechanistic inferences;

*ClinFlag=0;

ClearOrNot="unclear";

Prob="";

Magni="";

ORPosNeg=.; ORNegPos=.;

if ProbNeg<0.05 or ProbPos<0.05 then ClearOrNot="@90% ";

if ProbNeg<0.005 or ProbPos<0.005 then ClearOrNot="@99% ";

if ClearOrNot ne "unclear" then do;

Magni="+ive ";

if estimate<0 then Magni="-ive ";

if ProbPos>0.05 or ProbNeg>0.05 then Prob="unlikely";

if ProbPos>0.25 or ProbNeg>0.25 then Prob=“about as likely as not”;

if ProbPos>0.75 or ProbNeg>0.75 then Prob="likely ";

if ProbPos>0.95 or ProbNeg>0.95 then Prob="v.likely";

if ProbPos>0.995 or ProbNeg>0.995 then Prob=“virtually certain”;

end;

if ClearOrNot ne "unclear" and ProbTriv>75 then do;

Magni="triv.";

Prob="likely ";

if ProbTriv>0.95 then Prob="v.likely";

if ProbTriv>0.995 then Prob=“virtually certain”;

end;

*end;

output;

*/

/*

data est2;

Units="Raw ";

if &logflag then Units="factor";

set est1;

if estimate=0 then do;

estimate=.; magnithresh=.; magni=""; clearornot=""; Prob=""; Units="";

end;

array a estimate lower upper;

if &logflag then do over a;

a=exp(a/100); *all are factors;

end;

CLtd=sqrt(Upper/lower);

rename df=DegFree;

run;

*/

**data** est3;

set est2;

*if substr(Label,1,1)="." then delete;

*if substr(Label,1,1)="*" then Label=substr(Label,2);

*options ls=135 ps=80;

title5 "Non-clinical inferences";

title6 "Magnithresh is smallest change";

**proc** **print** data=est3 noobs;

*where clinflag=0;

var label estimate lower upper ES ESlower ESupper alpha DF

MagniThresh ProbNeg ProbTriv ProbPos Probt Strgth Magni ClearOrNot;

format estimate CLpm lower upper MagniThresh **5.1** ES ESlower ESupper **5.2** Probt best5. ProbNeg ProbTriv

ProbPos **5.4** DF **5.0**;

**run**;

**B. Full analysis with CHO modifier adjustments**

filename in "C:\Users\dsrowlan\OneDrive - Massey University\

Documents\Massey\Massey_2020\Research\Frucor_Hydration Review\

ECSS_2018_Manuscript meta-analysis\New_Dec 2019\

Working SAS_Feb 2020\Dat12wCHO_2.txt";

options linesize=**150** pagesize=**500**;

**data** dat12;

infile in missover firstobs=**2** delimiter='09'x;

*RowNo=_n_;

length Study $ **30** ; *length Strgth $ 10 Magni $ 4;

input StudyID ExptID EstimateID Study $ Source $ contrast

constrastn studytype $ Treat $ subjectn ex_mode $ ex_intensity_PCVOtwomax

Votwomax_ml_kg_min Bodyweight Votwomax_l_min metabolicrate

thermal_conditions $ TempC Humidity time drink_vol

Total_fluid avg_ingestion_rate drinkOsM_measured units $

drinkOsM_calc binOsm SD dPV SEM PV_Method $ logSD SElogSD InvSE2

folddPV logdPV DrinkOsM Pred StdErrPred DF Alpha Lower Upper

Resid Var SE WiRE meanhumidity meanTempC tempF heatindex

TimeBin $ CHOConc_Tot__gPC Frucconc_gPC glucconc_gPC sucrconc_gPC

maltoseconc_gPC Polyconc_gPC FrDi FrPoly Effective_Frucratio NamEq

KmEq MgmEq CamEq;

**run**;

**Data** dat13;

set dat12;

mEqtot=(**2***NamEq)+(**2***KmEq)+(MgmEq***3**)+(CamEq***3**);*units are mEq/L;

DrinkOsMAdj=(CHOConc_Tot__gPC/**180*****1000**/**0.1**)+mEqtot+**10**;*10 to allow for flavourings in drinks and background salts in water;

if Treat="Water" then DrinkOsMAdj=**0**;

**run**;

title8 "Raw Means ALL for covariates";

**proc** **means** data=dat13 maxdec=**1**;

var heatindex TempC Humidity avg_ingestion_rate metabolicrate drinkOsM CHOConc_Tot__gPC Frucconc_gPC

glucconc_gPC sucrconc_gPC maltoseconc_gPC Polyconc_gPC FrDi FrPoly Effective_Frucratio mEqtot DrinkOsMAdj;*drinkOsM;

*by Treat;

output out=meansallcovariates mean=mean stddev=SD min=min max=max;

**run**;

**proc** **sort** data=dat13;

by Treat;

**run**;

title9 "Raw Means BY TREAT for covariates- note Dat12 input aveinrate and MR already std'ised";

**proc** **means** data=dat13 maxdec=**2**;

var heatindex TempC Humidity avg_ingestion_rate metabolicrate drinkOsM CHOConc_Tot__gPC Frucconc_gPC

glucconc_gPC sucrconc_gPC maltoseconc_gPC Polyconc_gPC FrDi FrPoly Effective_Frucratio mEqtot DrinkOsMAdj;*drinkOsM;

by Treat;

output out=meanstreatcovariates mean=mean stddev=SD min=min max=max;

**run**;

**data** dat14;

set dat13;

*if treat ne "water";

**run**;

**proc** **standard** data=dat14 out=dat11 mean=**0** std=**0.5** noprint;

var heatindex TempC Humidity CHOConc_Tot__gPC

Frucconc_gPC glucconc_gPC sucrconc_gPC maltoseconc_gPC Polyconc_gPC FrDi FrPoly Effective_Frucratio mEqtot;*for the coefficients, 1 gives 2SD; *drinkOsM not standarised;

*by ex_type;

**run**;

**Treat: hyper hypo iso water;;

ods listing close;

**proc** **mixed** covtest data=dat11 cl alpha=**0.1** nobound CONVH=**1E-6** convf=**1E-6**;*nobound;*forcing it to converge at 6 dp;

class StudyID TimeBin ExptID Treat EstimateID;

weight WiRE;

*model dPV=TimeBin*treat/noint cl s DDFM=satalpha=0.1;

*standarised covariates for later avg_ingestion_rate*Treat heatindex*Treat metabolicrate*Treat;

*modifying and mediating covariates; *heatindex was found not to be an important modifer, so not continued with;

*model dPV=TimeBin*Treat TimeBin*Treat*DrinkOsM TimeBin*Treat*avg_ingestion_rate TimeBin*Treat*avg_ingestion_rate TimeBin*Treat*heatindex/noint cl DDFM=sat outp=pred outpm=predm residual alpha=0.1; *model to generate residuals for t vs SE plot for assymetry. When running turn off random effects;

*model dPV=TimeBin*Treat TimeBin*Treat*DrinkOsM TimeBin*Treat*metabolicrate/noint cl DDFM=sat outp=pred outpm=predm residual alpha=0.1;

*model dPV=TimeBin*Treat TimeBin*Treat*DrinkOsM TimeBin*Treat*avg_ingestion_rate/noint cl DDFM=sat outp=pred outpm=predm residual alpha=0.1;

*model dPV=TimeBin*Treat TimeBin*Treat*DrinkOsM TimeBin*Treat*heatindex/noint cl DDFM=sat outp=pred outpm=predm residual alpha=0.1;

*model dPV=TimeBin*Treat TimeBin*Treat*DrinkOsM/noint cl s DDFM=sat outp=pred outpm=predm residual alpha=0.1;

*model dPV=TimeBin*Treat TimeBin*Treat*heatindex TimeBin*Treat*metabolicrate TimeBin*Treat*avg_ingestion_rate/noint cl s DDFM=sat outp=pred outpm=predm alpha=0.1;

*model dPV=TimeBin*Treat TimeBin*Treat*DrinkOsM TimeBin*Treat*metabolicrate TimeBin*Treat*avg_ingestion_rate/noint cl DDFM=sat outp=pred outpm=predm alpha=0.1;

**full model with adjustment for carbohydrate properties;

**how - two indicator dummy variables. Varaibe 1-proportion of Disaccharide, variable 2 - propotion as polysacharide. mono=0 and Di=fraction of drink as a Di and Poly=fraction of drink as Poly;

*1. run model adjusted for CHO but with input Drink OsM; *TimeBin*Treat*FrDi TimeBin*Treat*FrPoly TimeBin*Treat*mEqtot;

* ;

*design modifers with effects: TimeBin*Treat*avg_ingestion_rate TimeBin*Treat*metabolicrate;

*CHO Modofers;

*confirmed substantial independent modifiers. All 3 together completely distorts the

treatment effects: TimeBin*Treat*CHOConc_Tot__gPC TimeBin*Treat*Effective_Frucratio TimeBin*Treat*FrDi;

*confirmed minor modifier, : TimeBin*Treat*FrPoly;

*confired minor modifer, : TimeBin*Treat*mEqtot;

model dPV=TimeBin*Treat TimeBin*Treat*DrinkOsM

TimeBin*Treat*mEqtot

/noint cl DDFM=sat outp=pred outpm=predm alpha=**0.1**;

*2. to estimate the raw, at gut wall, CHO effects, run model but with Effective Functional OsM, adjused for [CHO] and F:G;

*model dPV=TimeBin*Treat TimeBin*Treat*DrinkOsMAdj TimeBin*Treat*metabolicrate TimeBin*Treat*avg_ingestion_rate

TimeBin*Treat*CHOConc_Tot__gPC TimeBin*Treat*Effective_Frucratio/noint cl DDFM=sat outp=pred outpm=predm alpha=0.1;

*random int ExptID Timebin*Treat/subject=StudyID ;*Treat*StudyID, Int is StudyID;

random StudyID StudyID*ExptID StudyID*EstimateID/;

Parms **5** **1** **1** **1**/hold=**4**;

*clamping residual covariance to 1 means the weighting factor is the random effect for each dependent variable. Residual can be negative variance in this instance;

*references: Cassar et al 2016 Human Reproduction, pp. 1–13, 2016 doi:10.1093/humrep/dew243;

*Weston 2014 Sports Med (2014) 44:1005–1017, DOI 10.1007/s40279-014-0180-z;

*Yang 2003 A review of random effects modelling in SAS (release 8.2);

****do for Osm e.g. 350 600 1200... 220 150. range of OsM generally2SD accros studies, but something sensible;

*****finsih updateing 2SD coeffecients:;

/*

estimate " ";

estimate "modifying effect of covars on dPV";

estimate "after ajusting for CHO properties";

estimate "Effects of 2SD on dPV";

estimate "DrinkOsM all" TimeBin*Treat*DrinkOsM 178.2 127.4 16.2 0 178.2 127.4 16.2 0 178.2 127.4 16.2 0/cl divisor=12 alpha=0.1;

estimate "DrinkOsM hyper" TimeBin*Treat*DrinkOsM 178.2 0 0 0 178.2 0 0 0 178.2 0 0 0/cl divisor=3 alpha=0.1;

estimate "DrinkOsM hypo" TimeBin*Treat*DrinkOsM 0 127.4 0 0 0 127.4 0 0 0 127.4 0 0/cl divisor=3 alpha=0.1;

estimate "DrinkOsM iso" TimeBin*Treat*DrinkOsM 0 0 16.2 0 0 0 16.2 0 0 0 16.2 0/cl divisor=3 alpha=0.1;

estimate "DrinkOsM water" TimeBin*Treat*DrinkOsM 0 0 0 0 0 0 0 0 0 0 0 0/cl divisor=3 alpha=0.1;

estimate "Timebin1. ";

estimate "DrinkOsM all" TimeBin*Treat*DrinkOsM 178.2 127.4 16.2 0 0 0 0 0 0 0 0 0/cl divisor=4 alpha=0.1;

estimate "DrinkOsM hyper" TimeBin*Treat*DrinkOsM 178.2 0 0 0 0 0 0 0 0 0 0 0/cl divisor=1 alpha=0.1;

estimate "DrinkOsM hypo" TimeBin*Treat*DrinkOsM 0 127.4 0 0 0 0 0 0 0 0 0/cl divisor=1 alpha=0.1;

estimate "DrinkOsM iso" TimeBin*Treat*DrinkOsM 0 0 16.2 0 0 0 0 0 0 0 0 0 0/cl divisor=1 alpha=0.1;

estimate "DrinkOsM water" TimeBin*Treat*DrinkOsM 0 0 0 0 0 0 0 0 0 0 0 0/cl divisor=1 alpha=0.1;

estimate "Timebin2. ";

estimate "DrinkOsM all" TimeBin*Treat*DrinkOsM 0 0 0 0 178.2 127.4 16.2 0 0 0 0 0/cl divisor=4 alpha=0.1;

estimate "DrinkOsM hyper" TimeBin*Treat*DrinkOsM 0 0 0 0 178.2 0 0 0 0 0 0 0/cl divisor=1 alpha=0.1;

estimate "DrinkOsM hypo" TimeBin*Treat*DrinkOsM 0 0 0 0 0 127.4 0 0 0 0 0 0/cl divisor=1 alpha=0.1;

estimate "DrinkOsM iso" TimeBin*Treat*DrinkOsM 0 0 0 0 0 0 16.2 0 0 0 0 0/cl divisor=1 alpha=0.1;

estimate "DrinkOsM water" TimeBin*Treat*DrinkOsM 0 0 0 0 0 0 0 0 0 0 0 0/cl divisor=1 alpha=0.1;

estimate "Timebin3. ";

estimate "DrinkOsM all" TimeBin*Treat*DrinkOsM 0 0 0 0 0 0 0 0 178.2 127.4 16.2 0/cl divisor=4 alpha=0.1;

estimate "DrinkOsM hyper" TimeBin*Treat*DrinkOsM 0 0 0 0 0 0 0 0 178.2 0 0 0/cl divisor=1 alpha=0.1;

estimate "DrinkOsM hypo" TimeBin*Treat*DrinkOsM 0 0 0 0 0 0 0 0 0 127.4 0 0/cl divisor=1 alpha=0.1;

estimate "DrinkOsM iso" TimeBin*Treat*DrinkOsM 0 0 0 0 0 0 0 0 0 0 16.2 0/cl divisor=1 alpha=0.1;

estimate "DrinkOsM water" TimeBin*Treat*DrinkOsM 0 0 0 0 0 0 0 0 0 0 0 0/cl divisor=1 alpha=0.1;

*/

/*

estimate " ";

estimate "Effects of 2SD Metabolicrate";

estimate "Metabolicrate all" TimeBin*Treat*metabolicrate 1 1 1 1 1 1 1 1 1 1 1 1/cl divisor=12 alpha=0.1;

estimate "Metabolicrate hyper" TimeBin*Treat*metabolicrate 1 0 0 0 1 0 0 0 1 0 0 0/cl divisor=3 alpha=0.1;

estimate "Metabolicrate hypo" TimeBin*Treat*metabolicrate 0 1 0 0 0 1 0 0 0 1 0 0/cl divisor=3 alpha=0.1;

estimate "Metabolicrate iso" TimeBin*Treat*metabolicrate 0 0 1 0 0 0 1 0 0 0 1 0/cl divisor=3 alpha=0.1;

estimate "Metabolicrate water" TimeBin*Treat*metabolicrate 0 0 0 1 0 0 0 1 0 0 0 1/cl divisor=3 alpha=0.1;

estimate "Timebin1. ";

estimate "Metabolicrate all" TimeBin*Treat*metabolicrate 1 1 1 1 0 0 0 0 0 0 0 0/cl divisor=4 alpha=0.1;

estimate "Metabolicrate hyper" TimeBin*Treat*metabolicrate 1 0 0 0 0 0 0 0 0 0 0 0/cl divisor=1 alpha=0.1;

estimate "Metabolicrate hypo" TimeBin*Treat*metabolicrate 0 1 0 0 0 0 0 0 0 0 0/cl divisor=1 alpha=0.1;

estimate "Metabolicrate iso" TimeBin*Treat*metabolicrate 0 0 1 0 0 0 0 0 0 0 0 0 0/cl divisor=1 alpha=0.1;

estimate "Metabolicrate water" TimeBin*Treat*metabolicrate 0 0 0 1 0 0 0 0 0 0 0 0/cl divisor=1 alpha=0.1;

estimate "Timebin2. ";

estimate "Metabolicrate all" TimeBin*Treat*metabolicrate 0 0 0 0 1 1 1 1 0 0 0 0/cl divisor=4 alpha=0.1;

estimate "Metabolicrate hyper" TimeBin*Treat*metabolicrate 0 0 0 0 1 0 0 0 0 0 0 0/cl divisor=1 alpha=0.1;

estimate "Metabolicrate hypo" TimeBin*Treat*metabolicrate 0 0 0 0 0 1 0 0 0 0 0 0/cl divisor=1 alpha=0.1;

estimate "Metabolicrate iso" TimeBin*Treat*metabolicrate 0 0 0 0 0 0 1 0 0 0 0 0/cl divisor=1 alpha=0.1;

estimate "Metabolicrate water" TimeBin*Treat*metabolicrate 0 0 0 0 0 0 0 1 0 0 0 0/cl divisor=1 alpha=0.1;

estimate "Timebin3. ";

estimate "Metabolicrate all" TimeBin*Treat*metabolicrate 0 0 0 0 0 0 0 0 1 1 1 1/cl divisor=4 alpha=0.1;

estimate "Metabolicrate hyper" TimeBin*Treat*metabolicrate 0 0 0 0 0 0 0 0 1 0 0 0/cl divisor=1 alpha=0.1;

estimate "Metabolicrate hypo" TimeBin*Treat*metabolicrate 0 0 0 0 0 0 0 0 0 1 0 0/cl divisor=1 alpha=0.1;

estimate "Metabolicrate iso" TimeBin*Treat*metabolicrate 0 0 0 0 0 0 0 0 0 0 1 0/cl divisor=1 alpha=0.1;

estimate "Metabolicrate water" TimeBin*Treat*metabolicrate 0 0 0 0 0 0 0 0 0 0 0 1/cl divisor=1 alpha=0.1;

*/

/*

estimate "heatindex all" TimeBin*Treat*heatindex 1 1 1 1 1 1 1 1 1 1 1 1/cl divisor=12 alpha=0.1;

estimate "heatindex hyper" TimeBin*Treat*heatindex 1 0 0 0 1 0 0 0 1 0 0 0/cl divisor=3 alpha=0.1;

estimate "heatindex hypo" TimeBin*Treat*heatindex 0 1 0 0 0 1 0 0 0 1 0 0/cl divisor=3 alpha=0.1;

estimate "heatindex iso" TimeBin*Treat*heatindex 0 0 1 0 0 0 1 0 0 0 1 0/cl divisor=3 alpha=0.1;

estimate "heatindex water" TimeBin*Treat*heatindex 0 0 0 1 0 0 0 1 0 0 0 1/cl divisor=3 alpha=0.1;

*/

/*

estimate " ";

estimate "Effects of 2SD AvgIngestionRate";

estimate "AvgIngestionRate all" TimeBin*Treat*avg_ingestion_rate 1 1 1 1 1 1 1 1 1 1 1 1/cl divisor=12 alpha=0.1;

estimate "AvgIngestionRate hyper" TimeBin*Treat*avg_ingestion_rate 1 0 0 0 1 0 0 0 1 0 0 0/cl divisor=3 alpha=0.1;

estimate "AvgIngestionRate hypo" TimeBin*Treat*avg_ingestion_rate 0 1 0 0 0 1 0 0 0 1 0 0/cl divisor=3 alpha=0.1;

estimate "AvgIngestionRate iso" TimeBin*Treat*avg_ingestion_rate 0 0 1 0 0 0 1 0 0 0 1 0/cl divisor=3 alpha=0.1;

estimate "AvgIngestionRate water" TimeBin*Treat*avg_ingestion_rate 0 0 0 1 0 0 0 1 0 0 0 1/cl divisor=3 alpha=0.1;

estimate "Timebin1. ";

estimate "AvgIngestionRate all" TimeBin*Treat*avg_ingestion_rate 1 1 1 1 0 0 0 0 0 0 0 0/cl divisor=4 alpha=0.1;

estimate "AvgIngestionRate hyper" TimeBin*Treat*avg_ingestion_rate 1 0 0 0 0 0 0 0 0 0 0 0/cl divisor=1 alpha=0.1;

estimate "AvgIngestionRate hypo" TimeBin*Treat*avg_ingestion_rate 0 1 0 0 0 0 0 0 0 0 0/cl divisor=1 alpha=0.1;

estimate "AvgIngestionRate iso" TimeBin*Treat*avg_ingestion_rate 0 0 1 0 0 0 0 0 0 0 0 0 0/cl divisor=1 alpha=0.1;

estimate "AvgIngestionRate water" TimeBin*Treat*avg_ingestion_rate 0 0 0 1 0 0 0 0 0 0 0 0/cl divisor=1 alpha=0.1;

estimate "Timebin2. ";

estimate "AvgIngestionRate all" TimeBin*Treat*avg_ingestion_rate 0 0 0 0 1 1 1 1 0 0 0 0/cl divisor=4 alpha=0.1;

estimate "AvgIngestionRate hyper" TimeBin*Treat*avg_ingestion_rate 0 0 0 0 1 0 0 0 0 0 0 0/cl divisor=1 alpha=0.1;

estimate "AvgIngestionRate hypo" TimeBin*Treat*avg_ingestion_rate 0 0 0 0 0 1 0 0 0 0 0 0/cl divisor=1 alpha=0.1;

estimate "AvgIngestionRate iso" TimeBin*Treat*avg_ingestion_rate 0 0 0 0 0 0 1 0 0 0 0 0/cl divisor=1 alpha=0.1;

estimate "AvgIngestionRate water" TimeBin*Treat*avg_ingestion_rate 0 0 0 0 0 0 0 1 0 0 0 0/cl divisor=1 alpha=0.1;

estimate "Timebin3. ";

estimate "AvgIngestionRate all" TimeBin*Treat*avg_ingestion_rate 0 0 0 0 0 0 0 0 1 1 1 1/cl divisor=4 alpha=0.1;

estimate "AvgIngestionRate hyper" TimeBin*Treat*avg_ingestion_rate 0 0 0 0 0 0 0 0 1 0 0 0/cl divisor=1 alpha=0.1;

estimate "AvgIngestionRate hypo" TimeBin*Treat*avg_ingestion_rate 0 0 0 0 0 0 0 0 0 1 0 0/cl divisor=1 alpha=0.1;

estimate "AvgIngestionRate iso" TimeBin*Treat*avg_ingestion_rate 0 0 0 0 0 0 0 0 0 0 1 0/cl divisor=1 alpha=0.1;

estimate "AvgIngestionRate water" TimeBin*Treat*avg_ingestion_rate 0 0 0 0 0 0 0 0 0 0 0 1/cl divisor=1 alpha=0.1;

*/

/*

estimate " ";

estimate "Effects of 2SD FrDi";

estimate "FrDi all" TimeBin*Treat*FrDi 1 1 1 1 1 1 1 1 1 1 1 1/cl divisor=12 alpha=0.1;

estimate "FrDi hyper" TimeBin*Treat*FrDi 1 0 0 0 1 0 0 0 1 0 0 0/cl divisor=3 alpha=0.1;

estimate "FrDi hypo" TimeBin*Treat*FrDi 0 1 0 0 0 1 0 0 0 1 0 0/cl divisor=3 alpha=0.1;

estimate "FrDi iso" TimeBin*Treat*FrDi 0 0 1 0 0 0 1 0 0 0 1 0/cl divisor=3 alpha=0.1;

*estimate "FrDi water" TimeBin*Treat*FrDi 0 0 0 1 0 0 0 1 0 0 0 1/cl divisor=3 alpha=0.1;

estimate "Timebin1. ";

estimate "FrDi all" TimeBin*Treat*FrDi 1 1 1 1 0 0 0 0 0 0 0 0/cl divisor=4 alpha=0.1;

estimate "FrDi hyper" TimeBin*Treat*FrDi 1 0 0 0 0 0 0 0 0 0 0 0/cl divisor=1 alpha=0.1;

estimate "FrDi hypo" TimeBin*Treat*FrDi 0 1 0 0 0 0 0 0 0 0 0/cl divisor=1 alpha=0.1;

estimate "FrDi iso" TimeBin*Treat*FrDi 0 0 1 0 0 0 0 0 0 0 0 0 0/cl divisor=1 alpha=0.1;

*estimate "FrDi water" TimeBin*Treat*FrDi 0 0 0 1 0 0 0 0 0 0 0 0/cl divisor=1 alpha=0.1;

estimate "Timebin2. ";

estimate "FrDi all" TimeBin*Treat*FrDi 0 0 0 0 1 1 1 1 0 0 0 0/cl divisor=4 alpha=0.1;

estimate "FrDi hyper" TimeBin*Treat*FrDi 0 0 0 0 1 0 0 0 0 0 0 0/cl divisor=1 alpha=0.1;

estimate "FrDi hypo" TimeBin*Treat*FrDi 0 0 0 0 0 1 0 0 0 0 0 0/cl divisor=1 alpha=0.1;

estimate "FrDi iso" TimeBin*Treat*FrDi 0 0 0 0 0 0 1 0 0 0 0 0/cl divisor=1 alpha=0.1;

*estimate "FrDi water" TimeBin*Treat*FrDi 0 0 0 0 0 0 0 1 0 0 0 0/cl divisor=1 alpha=0.1;

estimate "Timebin3. ";

estimate "FrDi all" TimeBin*Treat*FrDi 0 0 0 0 0 0 0 0 1 1 1 1/cl divisor=4 alpha=0.1;

estimate "FrDi hyper" TimeBin*Treat*FrDi 0 0 0 0 0 0 0 0 1 0 0 0/cl divisor=1 alpha=0.1;

estimate "FrDi hypo" TimeBin*Treat*FrDi 0 0 0 0 0 0 0 0 0 1 0 0/cl divisor=1 alpha=0.1;

estimate "FrDi iso" TimeBin*Treat*FrDi 0 0 0 0 0 0 0 0 0 0 1 0/cl divisor=1 alpha=0.1;

*estimate "FrDi water" TimeBin*Treat*FrDi 0 0 0 0 0 0 0 0 0 0 0 1/cl divisor=1 alpha=0.1;

*/

/*

estimate " ";

estimate "Effects of 2SD FrPoly";

estimate "FrPoly all" TimeBin*Treat*FrPoly 1 1 1 1 1 1 1 1 1 1 1 1/cl divisor=12 alpha=0.1;

estimate "FrPoly hyper" TimeBin*Treat*FrPoly 1 0 0 0 1 0 0 0 1 0 0 0/cl divisor=3 alpha=0.1;

estimate "FrPoly hypo" TimeBin*Treat*FrPoly 0 1 0 0 0 1 0 0 0 1 0 0/cl divisor=3 alpha=0.1;

estimate "FrPoly iso" TimeBin*Treat*FrPoly 0 0 1 0 0 0 1 0 0 0 1 0/cl divisor=3 alpha=0.1;

*estimate "FrPoly water" TimeBin*Treat*FrPoly 0 0 0 1 0 0 0 1 0 0 0 1/cl divisor=3 alpha=0.1;

estimate "Timebin1. ";

estimate "FrPoly all" TimeBin*Treat*FrPoly 1 1 1 1 0 0 0 0 0 0 0 0/cl divisor=4 alpha=0.1;

estimate "FrPoly hyper" TimeBin*Treat*FrPoly 1 0 0 0 0 0 0 0 0 0 0 0/cl divisor=1 alpha=0.1;

estimate "FrPoly hypo" TimeBin*Treat*FrPoly 0 1 0 0 0 0 0 0 0 0 0/cl divisor=1 alpha=0.1;

estimate "FrPoly iso" TimeBin*Treat*FrPoly 0 0 1 0 0 0 0 0 0 0 0 0 0/cl divisor=1 alpha=0.1;

*estimate "FrPoly water" TimeBin*Treat*FrPoly 0 0 0 1 0 0 0 0 0 0 0 0/cl divisor=1 alpha=0.1;

estimate "Timebin2. ";

estimate "FrPoly all" TimeBin*Treat*FrPoly 0 0 0 0 1 1 1 1 0 0 0 0/cl divisor=4 alpha=0.1;

estimate "FrPoly hyper" TimeBin*Treat*FrPoly 0 0 0 0 1 0 0 0 0 0 0 0/cl divisor=1 alpha=0.1;

estimate "FrPoly hypo" TimeBin*Treat*FrPoly 0 0 0 0 0 1 0 0 0 0 0 0/cl divisor=1 alpha=0.1;

estimate "FrPoly iso" TimeBin*Treat*FrPoly 0 0 0 0 0 0 1 0 0 0 0 0/cl divisor=1 alpha=0.1;

*estimate "FrPoly water" TimeBin*Treat*FrPoly 0 0 0 0 0 0 0 1 0 0 0 0/cl divisor=1 alpha=0.1;

estimate "Timebin3. ";

estimate "FrPoly all" TimeBin*Treat*FrPoly 0 0 0 0 0 0 0 0 1 1 1 1/cl divisor=4 alpha=0.1;

estimate "FrPoly hyper" TimeBin*Treat*FrPoly 0 0 0 0 0 0 0 0 1 0 0 0/cl divisor=1 alpha=0.1;

estimate "FrPoly hypo" TimeBin*Treat*FrPoly 0 0 0 0 0 0 0 0 0 1 0 0/cl divisor=1 alpha=0.1;

estimate "FrPoly iso" TimeBin*Treat*FrPoly 0 0 0 0 0 0 0 0 0 0 1 0/cl divisor=1 alpha=0.1;

*estimate "FrPoly water" TimeBin*Treat*FrPoly 0 0 0 0 0 0 0 0 0 0 0 1/cl divisor=1 alpha=0.1;

*/

/*

estimate " ";

estimate "Effects of 2SD CHOConc_Tot__gPC";

estimate "CHOConc_Tot__gPC all" TimeBin*Treat*CHOConc_Tot__gPC 1 1 1 1 1 1 1 1 1 1 1 1/cl divisor=12 alpha=0.1;

estimate "CHOConc_Tot__gPC hyper" TimeBin*Treat*CHOConc_Tot__gPC 1 0 0 0 1 0 0 0 1 0 0 0/cl divisor=3 alpha=0.1;

estimate "CHOConc_Tot__gPC hypo" TimeBin*Treat*CHOConc_Tot__gPC 0 1 0 0 0 1 0 0 0 1 0 0/cl divisor=3 alpha=0.1;

estimate "CHOConc_Tot__gPC iso" TimeBin*Treat*CHOConc_Tot__gPC 0 0 1 0 0 0 1 0 0 0 1 0/cl divisor=3 alpha=0.1;

*estimate "CHOConc_Tot__gPC water" TimeBin*Treat*CHOConc_Tot__gPC 0 0 0 1 0 0 0 1 0 0 0 1/cl divisor=3 alpha=0.1;

estimate "Timebin1. ";

estimate "CHOConc_Tot__gPC all" TimeBin*Treat*CHOConc_Tot__gPC 1 1 1 1 0 0 0 0 0 0 0 0/cl divisor=4 alpha=0.1;

estimate "CHOConc_Tot__gPC hyper" TimeBin*Treat*CHOConc_Tot__gPC 1 0 0 0 0 0 0 0 0 0 0 0/cl divisor=1 alpha=0.1;

estimate "CHOConc_Tot__gPC hypo" TimeBin*Treat*CHOConc_Tot__gPC 0 1 0 0 0 0 0 0 0 0 0/cl divisor=1 alpha=0.1;

estimate "CHOConc_Tot__gPC iso" TimeBin*Treat*CHOConc_Tot__gPC 0 0 1 0 0 0 0 0 0 0 0 0 0/cl divisor=1 alpha=0.1;

*estimate "CHOConc_Tot__gPC water" TimeBin*Treat*CHOConc_Tot__gPC 0 0 0 1 0 0 0 0 0 0 0 0/cl divisor=1 alpha=0.1;

estimate "Timebin2. ";

estimate "CHOConc_Tot__gPC all" TimeBin*Treat*CHOConc_Tot__gPC 0 0 0 0 1 1 1 1 0 0 0 0/cl divisor=4 alpha=0.1;

estimate "CHOConc_Tot__gPC hyper" TimeBin*Treat*CHOConc_Tot__gPC 0 0 0 0 1 0 0 0 0 0 0 0/cl divisor=1 alpha=0.1;

estimate "CHOConc_Tot__gPC hypo" TimeBin*Treat*CHOConc_Tot__gPC 0 0 0 0 0 1 0 0 0 0 0 0/cl divisor=1 alpha=0.1;

estimate "CHOConc_Tot__gPC iso" TimeBin*Treat*CHOConc_Tot__gPC 0 0 0 0 0 0 1 0 0 0 0 0/cl divisor=1 alpha=0.1;

*estimate "CHOConc_Tot__gPC water" TimeBin*Treat*CHOConc_Tot__gPC 0 0 0 0 0 0 0 1 0 0 0 0/cl divisor=1 alpha=0.1;

estimate "Timebin3. ";

estimate "CHOConc_Tot__gPC all" TimeBin*Treat*CHOConc_Tot__gPC 0 0 0 0 0 0 0 0 1 1 1 1/cl divisor=4 alpha=0.1;

estimate "CHOConc_Tot__gPC hyper" TimeBin*Treat*CHOConc_Tot__gPC 0 0 0 0 0 0 0 0 1 0 0 0/cl divisor=1 alpha=0.1;

estimate "CHOConc_Tot__gPC hypo" TimeBin*Treat*CHOConc_Tot__gPC 0 0 0 0 0 0 0 0 0 1 0 0/cl divisor=1 alpha=0.1;

estimate "CHOConc_Tot__gPC iso" TimeBin*Treat*CHOConc_Tot__gPC 0 0 0 0 0 0 0 0 0 0 1 0/cl divisor=1 alpha=0.1;

*estimate "CHOConc_Tot__gPC water" TimeBin*Treat*CHOConc_Tot__gPC 0 0 0 0 0 0 0 0 0 0 0 1/cl divisor=1 alpha=0.1;

*/

/*

estimate " ";

estimate "Effects of 2SD Effective_Frucratio";

estimate "Effective_Frucratio all" TimeBin*Treat*Effective_Frucratio 1 1 1 1 1 1 1 1 1 1 1 1/cl divisor=12 alpha=0.1;

estimate "Effective_Frucratio hyper" TimeBin*Treat*Effective_Frucratio 1 0 0 0 1 0 0 0 1 0 0 0/cl divisor=3 alpha=0.1;

estimate "Effective_Frucratio hypo" TimeBin*Treat*Effective_Frucratio 0 1 0 0 0 1 0 0 0 1 0 0/cl divisor=3 alpha=0.1;

estimate "Effective_Frucratio iso" TimeBin*Treat*Effective_Frucratio 0 0 1 0 0 0 1 0 0 0 1 0/cl divisor=3 alpha=0.1;

*estimate "Effective_Frucratio water" TimeBin*Treat*Effective_Frucratio 0 0 0 1 0 0 0 1 0 0 0 1/cl divisor=3 alpha=0.1;

estimate "Timebin1. ";

estimate "Effective_Frucratio all" TimeBin*Treat*Effective_Frucratio 1 1 1 1 0 0 0 0 0 0 0 0/cl divisor=4 alpha=0.1;

estimate "Effective_Frucratio hyper" TimeBin*Treat*Effective_Frucratio 1 0 0 0 0 0 0 0 0 0 0 0/cl divisor=1 alpha=0.1;

estimate "Effective_Frucratio hypo" TimeBin*Treat*Effective_Frucratio 0 1 0 0 0 0 0 0 0 0 0/cl divisor=1 alpha=0.1;

estimate "Effective_Frucratio iso" TimeBin*Treat*Effective_Frucratio 0 0 1 0 0 0 0 0 0 0 0 0 0/cl divisor=1 alpha=0.1;

*estimate "Effective_Frucratio water" TimeBin*Treat*Effective_Frucratio 0 0 0 1 0 0 0 0 0 0 0 0/cl divisor=1 alpha=0.1;

estimate "Timebin2. ";

estimate "Effective_Frucratio all" TimeBin*Treat*Effective_Frucratio 0 0 0 0 1 1 1 1 0 0 0 0/cl divisor=4 alpha=0.1;

estimate "Effective_Frucratio hyper" TimeBin*Treat*Effective_Frucratio 0 0 0 0 1 0 0 0 0 0 0 0/cl divisor=1 alpha=0.1;

estimate "Effective_Frucratio hypo" TimeBin*Treat*Effective_Frucratio 0 0 0 0 0 1 0 0 0 0 0 0/cl divisor=1 alpha=0.1;

estimate "Effective_Frucratio iso" TimeBin*Treat*Effective_Frucratio 0 0 0 0 0 0 1 0 0 0 0 0/cl divisor=1 alpha=0.1;

*estimate "Effective_Frucratio water" TimeBin*Treat*Effective_Frucratio 0 0 0 0 0 0 0 1 0 0 0 0/cl divisor=1 alpha=0.1;

estimate "Timebin3. ";

estimate "Effective_Frucratio all" TimeBin*Treat*Effective_Frucratio 0 0 0 0 0 0 0 0 1 1 1 1/cl divisor=4 alpha=0.1;

estimate "Effective_Frucratio hyper" TimeBin*Treat*Effective_Frucratio 0 0 0 0 0 0 0 0 1 0 0 0/cl divisor=1 alpha=0.1;

estimate "Effective_Frucratio hypo" TimeBin*Treat*Effective_Frucratio 0 0 0 0 0 0 0 0 0 1 0 0/cl divisor=1 alpha=0.1;

estimate "Effective_Frucratio iso" TimeBin*Treat*Effective_Frucratio 0 0 0 0 0 0 0 0 0 0 1 0/cl divisor=1 alpha=0.1;

*estimate "Effective_Frucratio water" TimeBin*Treat*Effective_Frucratio 0 0 0 0 0 0 0 0 0 0 0 1/cl divisor=1 alpha=0.1;

*/

/*

estimate " ";

estimate "Effects of 2SD mEqtot";

estimate "mEqtot all" TimeBin*Treat*mEqtot 1 1 1 1 1 1 1 1 1 1 1 1/cl divisor=12 alpha=0.1;

estimate "mEqtot hyper" TimeBin*Treat*mEqtot 1 0 0 0 1 0 0 0 1 0 0 0/cl divisor=3 alpha=0.1;

estimate "mEqtot hypo" TimeBin*Treat*mEqtot 0 1 0 0 0 1 0 0 0 1 0 0/cl divisor=3 alpha=0.1;

estimate "mEqtot iso" TimeBin*Treat*mEqtot 0 0 1 0 0 0 1 0 0 0 1 0/cl divisor=3 alpha=0.1;

estimate "mEqtot water" TimeBin*Treat*mEqtot 0 0 0 1 0 0 0 1 0 0 0 1/cl divisor=3 alpha=0.1;

estimate "Timebin1. ";

estimate "mEqtot all" TimeBin*Treat*mEqtot 1 1 1 1 0 0 0 0 0 0 0 0/cl divisor=4 alpha=0.1;

estimate "mEqtot hyper" TimeBin*Treat*mEqtot 1 0 0 0 0 0 0 0 0 0 0 0/cl divisor=1 alpha=0.1;

estimate "mEqtot hypo" TimeBin*Treat*mEqtot 0 1 0 0 0 0 0 0 0 0 0/cl divisor=1 alpha=0.1;

estimate "mEqtot iso" TimeBin*Treat*mEqtot 0 0 1 0 0 0 0 0 0 0 0 0 0/cl divisor=1 alpha=0.1;

estimate "mEqtot water" TimeBin*Treat*mEqtot 0 0 0 1 0 0 0 0 0 0 0 0/cl divisor=1 alpha=0.1;

estimate "Timebin2. ";

estimate "mEqtot all" TimeBin*Treat*mEqtot 0 0 0 0 1 1 1 1 0 0 0 0/cl divisor=4 alpha=0.1;

estimate "mEqtot hyper" TimeBin*Treat*mEqtot 0 0 0 0 1 0 0 0 0 0 0 0/cl divisor=1 alpha=0.1;

estimate "mEqtot hypo" TimeBin*Treat*mEqtot 0 0 0 0 0 1 0 0 0 0 0 0/cl divisor=1 alpha=0.1;

estimate "mEqtot iso" TimeBin*Treat*mEqtot 0 0 0 0 0 0 1 0 0 0 0 0/cl divisor=1 alpha=0.1;

estimate "mEqtot water" TimeBin*Treat*mEqtot 0 0 0 0 0 0 0 1 0 0 0 0/cl divisor=1 alpha=0.1;

estimate "Timebin3. ";

estimate "mEqtot all" TimeBin*Treat*mEqtot 0 0 0 0 0 0 0 0 1 1 1 1/cl divisor=4 alpha=0.1;

estimate "mEqtot hyper" TimeBin*Treat*mEqtot 0 0 0 0 0 0 0 0 1 0 0 0/cl divisor=1 alpha=0.1;

estimate "mEqtot hypo" TimeBin*Treat*mEqtot 0 0 0 0 0 0 0 0 0 1 0 0/cl divisor=1 alpha=0.1;

estimate "mEqtot iso" TimeBin*Treat*mEqtot 0 0 0 0 0 0 0 0 0 0 1 0/cl divisor=1 alpha=0.1;

estimate "mEqtot water" TimeBin*Treat*mEqtot 0 0 0 0 0 0 0 0 0 0 0 1/cl divisor=1 alpha=0.1;

*/

****;

estimate " ";

estimate "DrinkOsm Initial Analysis V.likely d Analysis";

estimate "DrinkOsm V.likely d Analysis";

estimate "TimeBinMeansOverall mean setting";*automatically adjusted for standardised covariates. If not standardised then need to put in the covariate;

estimate "Hyper" TimeBin*Treat **1** **0** **0** **0** **1** **0** **0** **0** **1** **0** **0** **0** TimeBin*Treat*DrinkOsM **98.6** **0** **0** **0** **98.6** **0** **0** **0** **98.6** **0** **0** **0**/cl divisor=**3** alpha=**0.1**;

estimate "Hypo" TimeBin*Treat **0** **1** **0** **0** **0** **1** **0** **0** **0** **1** **0** **0** TimeBin*Treat*DrinkOsM **0** -**108.2** **0** **0** **0** -**108.2** **0** **0** **0** -**108.2** **0** **0**/cl divisor=**3** alpha=**0.1**;

estimate "Iso" TimeBin*Treat **0** **0** **1** **0** **0** **0** **1** **0** **0** **0** **1** **0** TimeBin*Treat*DrinkOsM **0** **0** -**3.2** **0** **0** **0** -**3.2** **0** **0** **0** -**3.2** **0**/cl divisor=**3** alpha=**0.1**;

estimate "Water" TimeBin*Treat **0** **0** **0** **1** **0** **0** **0** **1** **0** **0** **0** **1** /cl divisor=**3** alpha=**0.1**;

estimate "Hyper-iso" TimeBin*Treat **1** **0** -**1** **0** **1** **0** -**1** **0** **1** **0** -**1** **0** TimeBin*Treat*DrinkOsM **98.6** **0** **3.2** **0** **98.6** **0** **3.2** **0** **98.6** **0** **3.2** **0**/cl divisor=**3** alpha=**0.1**;

estimate "Hypo-iso" TimeBin*Treat **0** **1** -**1** **0** **0** **1** -**1** **0** **0** **1** -**1** **0** TimeBin*Treat*DrinkOsM **0** -**108.2** **3.2** **0** **0** -**108.2** **3.2** **0** **0** -**108.2** **3.2** **0**/cl divisor=**3** alpha=**0.1**;

estimate "Water-iso" TimeBin*Treat **0** **0** -**1** **1** **0** **0** -**1** **1** **0** **0** -**1** **1** TimeBin*Treat*DrinkOsM **0** **0** **3.2** **0** **0** **0** **3.2** **0** **0** **0** **3.2** **0**/cl divisor=**3** alpha=**0.1**;

estimate "Hyper-Hypo" TimeBin*Treat **1** -**1** **0** **0** **1** -**1** **0** **0** **1** -**1** **0** **0** TimeBin*Treat*DrinkOsM **98.6** **108.2** **0** **0** **98.6** **108.2** **0** **0** **98.6** **103.1** **0** **0**/cl divisor=**3** alpha=**0.1**; *check iso is a postivie then ave covar is a -103.1;

estimate "Hyper-Water" TimeBin*Treat **1** **0** **0** -**1** **1** **0** **0** -**1** **1** **0** **0** -**1** TimeBin*Treat*DrinkOsM **98.6** **0** **0** **0** **98.6** **0** **0** **0** **98.6** **0** **0** **0**/cl divisor=**3** alpha=**0.1**;

estimate "Hypo-Water" TimeBin*Treat **0** **1** **0** -**1** **0** **1** **0** -**1** **0** **1** **0** -**1** TimeBin*Treat*DrinkOsM **0** -**108.2** **0** **0** **0** -**108.2** **0** **0** **0** -**108.2** **0** **0**/cl divisor=**3** alpha=**0.1**;

/*

estimate "TimeBinMeansOverall indiv setting";*automatically adjusted for standardised covariates. If not standardised then need to put in the covariate;

estimate "Hyper" TimeBin*Treat 1 0 0 0 1 0 0 0 1 0 0 0 TimeBin*Treat*DrinkOsM 98.6 0 0 0 98.6 0 0 0 98.6 0 0 0|StudyID 1 StudyID*ExptID 1 StudyID*EstimateID 1/cl divisor=3 alpha=0.1;

estimate "Hypo" TimeBin*Treat 0 1 0 0 0 1 0 0 0 1 0 0 TimeBin*Treat*DrinkOsM 0 -108.2 0 0 0 -108.2 0 0 0 -108.2 0 0|StudyID 1 StudyID*ExptID 1 StudyID*EstimateID 1/cl divisor=3 alpha=0.1;

estimate "Iso" TimeBin*Treat 0 0 1 0 0 0 1 0 0 0 1 0 TimeBin*Treat*DrinkOsM 0 0 -3.2 0 0 0 -3.2 0 0 0 -3.2 0|StudyID 1 StudyID*ExptID 1 StudyID*EstimateID 1/cl divisor=3 alpha=0.1;

estimate "Water" TimeBin*Treat 0 0 0 1 0 0 0 1 0 0 0 1 |StudyID 1 StudyID*ExptID 1 StudyID*EstimateID 1/cl divisor=3 alpha=0.1;

estimate "Hyper-iso" TimeBin*Treat 1 0 -1 0 1 0 -1 0 1 0 -1 0 TimeBin*Treat*DrinkOsM 98.6 0 3.2 0 98.6 0 3.2 0 98.6 0 3.2 0|StudyID 1 StudyID*ExptID 1 StudyID*EstimateID 1/cl divisor=3 alpha=0.1;

estimate "Hypo-iso" TimeBin*Treat 0 1 -1 0 0 1 -1 0 0 1 -1 0 TimeBin*Treat*DrinkOsM 0 -108.2 3.2 0 0 -108.2 3.2 0 0 -108.2 3.2 0|StudyID 1 StudyID*ExptID 1 StudyID*EstimateID 1/cl divisor=3 alpha=0.1;

estimate "Water-iso" TimeBin*Treat 0 0 -1 1 0 0 -1 1 0 0 -1 1 TimeBin*Treat*DrinkOsM 0 0 3.2 0 0 0 3.2 0 0 0 3.2 0|StudyID 1 StudyID*ExptID 1 StudyID*EstimateID 1/cl divisor=3 alpha=0.1;

estimate "Hyper-Hypo" TimeBin*Treat 1 -1 0 0 1 -1 0 0 1 -1 0 0 TimeBin*Treat*DrinkOsM 98.6 108.2 0 0 98.6 108.2 0 0 98.6 103.1 0 0|StudyID 1 StudyID*ExptID 1 StudyID*EstimateID 1/cl divisor=3 alpha=0.1; *check iso is a postivie then ave covar is a -103.1;

estimate "Hyper-Water" TimeBin*Treat 1 0 0 -1 1 0 0 -1 1 0 0 -1 TimeBin*Treat*DrinkOsM 98.6 0 0 0 98.6 0 0 0 98.6 0 0 0|StudyID 1 StudyID*ExptID 1 StudyID*EstimateID 1/cl divisor=3 alpha=0.1;

estimate "Hypo-Water" TimeBin*Treat 0 1 0 -1 0 1 0 -1 0 1 0 -1 TimeBin*Treat*DrinkOsM 0 -108.2 0 0 0 -108.2 0 0 0 -108.2 0 0|StudyID 1 StudyID*ExptID 1 StudyID*EstimateID 1/cl divisor=3 alpha=0.1;

estimate " ";

estimate "DrinkOsm V.likely d Analysis";

estimate "TimeBin1.<31 mean setting";

estimate "Hyper" TimeBin*Treat 1 0 0 0 0 0 0 0 0 0 0 0 TimeBin*Treat*DrinkOsM 98.6 0 0 0 0 0 0 0 0 0 0 0/cl divisor=1 alpha=0.1;

estimate "Hypo" TimeBin*Treat 0 1 0 0 0 0 0 0 0 0 0 0 TimeBin*Treat*DrinkOsM 0 -108.2 0 0 0 0 0 0 0 0 0 0/cl divisor=1 alpha=0.1;

estimate "Iso" TimeBin*Treat 0 0 1 0 0 0 0 0 0 0 0 0 TimeBin*Treat*DrinkOsM 0 0 -3.2 0 0 0 0 0 0 0 0 0/cl divisor=1 alpha=0.1;

estimate "Water" TimeBin*Treat 0 0 0 1 0 0 0 0 0 0 0 0 /cl divisor=1 alpha=0.1;

estimate "Hyper-iso" TimeBin*Treat 1 0 -1 0 0 0 0 0 0 0 0 0 TimeBin*Treat*DrinkOsM 98.6 0 3.2 0 0 0 0 0 0 0 0 0/cl divisor=1 alpha=0.1;

estimate "Hypo-iso" TimeBin*Treat 0 1 -1 0 0 0 0 0 0 0 0 0 TimeBin*Treat*DrinkOsM 0 -108.2 3.2 0 0 0 0 0 0 0 0 0/cl divisor=1 alpha=0.1;

estimate "Water-iso" TimeBin*Treat 0 0 -1 1 0 0 0 0 0 0 0 0 TimeBin*Treat*DrinkOsM 0 0 3.2 0 0 0 0 0 0 0 0 0/cl divisor=1 alpha=0.1;

estimate "Hyper-Hypo" TimeBin*Treat 1 -1 0 0 0 0 0 0 0 0 0 0 TimeBin*Treat*DrinkOsM 98.6 108.2 0 0 0 0 0 0 0 0 0 0/cl divisor=1 alpha=0.1; *check iso is a postivie then ave covar is a -103.1;

estimate "Hyper-Water" TimeBin*Treat 1 0 0 -1 0 0 0 0 0 0 0 0 TimeBin*Treat*DrinkOsM 98.6 0 0 0 0 0 0 0 0 0 0 0/cl divisor=1 alpha=0.1;

estimate "Hypo-Water" TimeBin*Treat 0 1 0 -1 0 0 0 0 0 0 0 0 TimeBin*Treat*DrinkOsM 0 -108.2 0 0 0 0 0 0 0 0 0 0/cl divisor=1 alpha=0.1;

estimate "TimeBin2.31-60 mean setting";

estimate "Hyper" TimeBin*Treat 0 0 0 0 1 0 0 0 0 0 0 0 TimeBin*Treat*DrinkOsM 0 0 0 0 98.6 0 0 0 0 0 0 0/cl divisor=1 alpha=0.1;

estimate "Hypo" TimeBin*Treat 0 0 0 0 0 1 0 0 0 0 0 0 TimeBin*Treat*DrinkOsM 0 0 0 0 0 -108.2 0 0 0 0 0 0/cl divisor=1 alpha=0.1;

estimate "Iso" TimeBin*Treat 0 0 0 0 0 0 1 0 0 0 0 0 TimeBin*Treat*DrinkOsM 0 0 0 0 0 0 -3.2 0 0 0 0 0/cl divisor=1 alpha=0.1;

estimate "Water" TimeBin*Treat 0 0 0 0 0 0 0 1 0 0 0 0 /cl divisor=1 alpha=0.1;

estimate "Hyper-iso" TimeBin*Treat 0 0 0 0 1 0 -1 0 0 0 0 0 TimeBin*Treat*DrinkOsM 0 0 0 0 98.6 0 3.2 0 0 0 0 0/cl divisor=1 alpha=0.1;

estimate "Hypo-iso" TimeBin*Treat 0 0 0 0 0 1 -1 0 0 0 0 0 TimeBin*Treat*DrinkOsM 0 0 0 0 0 -108.2 3.2 0 0 0 0 0/cl divisor=1 alpha=0.1;

estimate "Water-iso" TimeBin*Treat 0 0 0 0 0 0 -1 1 0 0 0 0 TimeBin*Treat*DrinkOsM 0 0 0 0 0 0 3.2 0 0 0 0 0/cl divisor=1 alpha=0.1;

estimate "Hyper-Hypo" TimeBin*Treat 0 0 0 0 1 -1 0 0 0 0 0 0 TimeBin*Treat*DrinkOsM 0 0 0 0 98.6 108.2 0 0 0 0 0 0/cl divisor=1 alpha=0.1; *check iso is a postivie then ave covar is a -103.1;

estimate "Hyper-Water" TimeBin*Treat 0 0 0 0 1 0 0 -1 0 0 0 0 TimeBin*Treat*DrinkOsM 0 0 0 0 98.6 0 0 0 0 0 0 0/cl divisor=1 alpha=0.1;

estimate "Hypo-Water" TimeBin*Treat 0 0 0 0 0 1 0 -1 0 0 0 0 TimeBin*Treat*DrinkOsM 0 0 0 0 0 -108.2 0 0 0 0 0 0/cl divisor=1 alpha=0.1;

estimate "TimeBin3.60-180 mean setting";

estimate "Hyper" TimeBin*Treat 0 0 0 0 0 0 0 0 1 0 0 0 TimeBin*Treat*DrinkOsM 0 0 0 0 0 0 0 0 98.6 0 0 0/cl divisor=1 alpha=0.1;

estimate "Hypo" TimeBin*Treat 0 0 0 0 0 0 0 0 0 1 0 0 TimeBin*Treat*DrinkOsM 0 0 0 0 0 0 0 0 0 -108.2 0 0/cl divisor=1 alpha=0.1;

estimate "Iso" TimeBin*Treat 0 0 0 0 0 0 0 0 0 0 1 0 TimeBin*Treat*DrinkOsM 0 0 0 0 0 0 0 0 0 0 -3.2 0/cl divisor=1 alpha=0.1;

estimate "Water" TimeBin*Treat 0 0 0 0 0 0 0 0 0 0 0 1 /cl divisor=1 alpha=0.1;

estimate "Hyper-iso" TimeBin*Treat 0 0 0 0 0 0 0 0 1 0 -1 0 TimeBin*Treat*DrinkOsM 0 0 0 0 0 0 0 0 98.6 0 3.2 0/cl divisor=1 alpha=0.1;

estimate "Hypo-iso" TimeBin*Treat 0 0 0 0 0 0 0 0 0 1 -1 0 TimeBin*Treat*DrinkOsM 0 0 0 0 0 0 0 0 0 -108.2 3.2 0/cl divisor=1 alpha=0.1;

estimate "Water-iso" TimeBin*Treat 0 0 0 0 0 0 0 0 0 0 -1 1 TimeBin*Treat*DrinkOsM 0 0 0 0 0 0 0 0 0 0 3.2 0/cl divisor=1 alpha=0.1;

estimate "Hyper-Hypo" TimeBin*Treat 0 0 0 0 0 0 0 0 1 -1 0 0 TimeBin*Treat*DrinkOsM 0 0 0 0 0 0 0 0 98.6 103.1 0 0/cl divisor=1 alpha=0.1; *check iso is a postivie then ave covar is a -103.1;

estimate "Hyper-Water" TimeBin*Treat 0 0 0 0 0 0 0 0 1 0 0 -1 TimeBin*Treat*DrinkOsM 0 0 0 0 0 0 0 0 98.6 0 0 0/cl divisor=1 alpha=0.1;

estimate "Hypo-Water" TimeBin*Treat 0 0 0 0 0 0 0 0 0 1 0 -1 TimeBin*Treat*DrinkOsM 0 0 0 0 0 0 0 0 0 -108.2 0 0/cl divisor=1 alpha=0.1;

*/

/*

estimate " ";

estimate "DrinkOsm V.likely d Analysis";

estimate "TimeBinMeansOverall mean setting";*automatically adjusted for standardised covariates. If not standardised then need to put in the covariate;

estimate "Hyper" TimeBin*Treat 1 0 0 0 1 0 0 0 1 0 0 0 TimeBin*Treat*DrinkOsM 98.6 0 0 0 98.6 0 0 0 98.6 0 0 0 TimeBin*Treat*FrDi 1 0 0 0 1 0 0 0 1 0 0 0 TimeBin*Treat*FrPoly 1 0 0 0 1 0 0 0 1 0 0 0/cl divisor=3 alpha=0.1;

estimate "Hypo" TimeBin*Treat 0 1 0 0 0 1 0 0 0 1 0 0 TimeBin*Treat*DrinkOsM 0 -108.2 0 0 0 -108.2 0 0 0 -108.2 0 0 TimeBin*Treat*FrDi 0 1 0 0 0 1 0 0 0 1 0 0 TimeBin*Treat*FrPoly 0 1 0 0 0 1 0 0 0 1 0 0/cl divisor=3 alpha=0.1;

estimate "Iso" TimeBin*Treat 0 0 1 0 0 0 1 0 0 0 1 0 TimeBin*Treat*DrinkOsM 0 0 -3.2 0 0 0 -3.2 0 0 0 -3.2 0 TimeBin*Treat*FrDi 0 0 1 0 0 0 1 0 0 0 1 0 TimeBin*Treat*FrPoly 0 0 1 0 0 0 1 0 0 0 1 0/cl divisor=3 alpha=0.1;

estimate "Water" TimeBin*Treat 0 0 0 1 0 0 0 1 0 0 0 1 TimeBin*Treat*FrDi 0 0 0 1 0 0 0 1 0 0 0 1 TimeBin*Treat*FrPoly 0 0 0 1 0 0 0 1 0 0 0 1/cl divisor=3 alpha=0.1;

estimate "Hyper-iso" TimeBin*Treat 1 0 -1 0 1 0 -1 0 1 0 -1 0 TimeBin*Treat*DrinkOsM 98.6 0 3.2 0 98.6 0 3.2 0 98.6 0 3.2 0 TimeBin*Treat*FrDi 1 0 -1 0 1 0 -1 0 1 0 -1 0 TimeBin*Treat*FrPoly 1 0 -1 0 1 0 -1 0 1 0 -1 0/cl divisor=3 alpha=0.1;

estimate "Hypo-iso" TimeBin*Treat 0 1 -1 0 0 1 -1 0 0 1 -1 0 TimeBin*Treat*DrinkOsM 0 -108.2 3.2 0 0 -108.2 3.2 0 0 -108.2 3.2 0 TimeBin*Treat*FrDi 0 1 -1 0 0 1 -1 0 0 1 -1 0 TimeBin*Treat*FrPoly 0 1 -1 0 0 1 -1 0 0 1 -1 0/cl divisor=3 alpha=0.1;

estimate "Water-iso" TimeBin*Treat 0 0 -1 1 0 0 -1 1 0 0 -1 1 TimeBin*Treat*DrinkOsM 0 0 3.2 0 0 0 3.2 0 0 0 3.2 0 TimeBin*Treat*FrDi 0 0 -1 1 0 0 -1 1 0 0 -1 1 TimeBin*Treat*FrPoly 0 0 -1 1 0 0 -1 1 0 0 -1 1/cl divisor=3 alpha=0.1;

estimate "Hyper-Hypo" TimeBin*Treat 1 -1 0 0 1 -1 0 0 1 -1 0 0 TimeBin*Treat*DrinkOsM 98.6 108.2 0 0 98.6 108.2 0 0 98.6 103.1 0 0 TimeBin*Treat*FrDi 1 -1 0 0 1 -1 0 0 1 -1 0 0TimeBin*Treat*FrPoly 1 -1 0 0 1 -1 0 0 1 -1 0 0/cl divisor=3 alpha=0.1; *check iso is a postivie then ave covar is a -103.1;

estimate "Hyper-Water" TimeBin*Treat 1 0 0 -1 1 0 0 -1 1 0 0 -1 TimeBin*Treat*DrinkOsM 98.6 0 0 0 98.6 0 0 0 98.6 0 0 0 TimeBin*Treat*FrDi 1 0 0 -1 1 0 0 -1 1 0 0 -1 TimeBin*Treat*FrPoly 1 0 0 -1 1 0 0 -1 1 0 0 -1/cl divisor=3 alpha=0.1;

estimate "Hypo-Water" TimeBin*Treat 0 1 0 -1 0 1 0 -1 0 1 0 -1 TimeBin*Treat*DrinkOsM 0 -108.2 0 0 0 -108.2 0 0 0 -108.2 0 0 TimeBin*Treat*FrDi 0 1 0 -1 0 1 0 -1 0 1 0 -1 TimeBin*Treat*FrPoly 0 1 0 -1 0 1 0 -1 0 1 0 -1/cl divisor=3 alpha=0.1;

estimate "TimeBinMeansOverall indiv setting";*automatically adjusted for standardised covariates. If not standardised then need to put in the covariate;

estimate "Hyper" TimeBin*Treat 1 0 0 0 1 0 0 0 1 0 0 0 TimeBin*Treat*DrinkOsM 98.6 0 0 0 98.6 0 0 0 98.6 0 0 0 TimeBin*Treat*FrDi 1 0 0 0 1 0 0 0 1 0 0 0 TimeBin*Treat*FrPoly 1 0 0 0 1 0 0 0 1 0 0 0|StudyID 1 StudyID*ExptID 1 StudyID*EstimateID 1/cl divisor=3 alpha=0.1;

estimate "Hypo" TimeBin*Treat 0 1 0 0 0 1 0 0 0 1 0 0 TimeBin*Treat*DrinkOsM 0 -108.2 0 0 0 -108.2 0 0 0 -108.2 0 0 TimeBin*Treat*FrDi 0 1 0 0 0 1 0 0 0 1 0 0 TimeBin*Treat*FrPoly 0 1 0 0 0 1 0 0 0 1 0 0|StudyID 1 StudyID*ExptID 1 StudyID*EstimateID 1/cl divisor=3 alpha=0.1;

estimate "Iso" TimeBin*Treat 0 0 1 0 0 0 1 0 0 0 1 0 TimeBin*Treat*DrinkOsM 0 0 -3.2 0 0 0 -3.2 0 0 0 -3.2 0 TimeBin*Treat*FrDi 0 0 1 0 0 0 1 0 0 0 1 0 TimeBin*Treat*FrPoly 0 0 1 0 0 0 1 0 0 0 1 0|StudyID 1 StudyID*ExptID 1 StudyID*EstimateID 1/cl divisor=3 alpha=0.1;

estimate "Water" TimeBin*Treat 0 0 0 1 0 0 0 1 0 0 0 1 TimeBin*Treat*FrDi 0 0 0 1 0 0 0 1 0 0 0 1 TimeBin*Treat*FrPoly 0 0 0 1 0 0 0 1 0 0 0 1|StudyID 1 StudyID*ExptID 1 StudyID*EstimateID 1/cl divisor=3 alpha=0.1;

estimate "Hyper-iso" TimeBin*Treat 1 0 -1 0 1 0 -1 0 1 0 -1 0 TimeBin*Treat*DrinkOsM 98.6 0 3.2 0 98.6 0 3.2 0 98.6 0 3.2 0 TimeBin*Treat*FrDi 1 0 -1 0 1 0 -1 0 1 0 -1 0 TimeBin*Treat*FrPoly 1 0 -1 0 1 0 -1 0 1 0 -1 0|StudyID 1 StudyID*ExptID 1 StudyID*EstimateID 1/cl divisor=3 alpha=0.1;

estimate "Hypo-iso" TimeBin*Treat 0 1 -1 0 0 1 -1 0 0 1 -1 0 TimeBin*Treat*DrinkOsM 0 -108.2 3.2 0 0 -108.2 3.2 0 0 -108.2 3.2 0 TimeBin*Treat*FrDi 0 1 -1 0 0 1 -1 0 0 1 -1 0 TimeBin*Treat*FrPoly 0 1 -1 0 0 1 -1 0 0 1 -1 0|StudyID 1 StudyID*ExptID 1 StudyID*EstimateID 1/cl divisor=3 alpha=0.1;

estimate "Water-iso" TimeBin*Treat 0 0 -1 1 0 0 -1 1 0 0 -1 1 TimeBin*Treat*DrinkOsM 0 0 3.2 0 0 0 3.2 0 0 0 3.2 0 TimeBin*Treat*FrDi 0 0 -1 1 0 0 -1 1 0 0 -1 1 TimeBin*Treat*FrPoly 0 0 -1 1 0 0 -1 1 0 0 -1 1|StudyID 1 StudyID*ExptID 1 StudyID*EstimateID 1/cl divisor=3 alpha=0.1;

estimate "Hyper-Hypo" TimeBin*Treat 1 -1 0 0 1 -1 0 0 1 -1 0 0 TimeBin*Treat*DrinkOsM 98.6 108.2 0 0 98.6 108.2 0 0 98.6 103.1 0 0 TimeBin*Treat*FrDi 1 -1 0 0 1 -1 0 0 1 -1 0 0TimeBin*Treat*FrPoly 1 -1 0 0 1 -1 0 0 1 -1 0 0|StudyID 1 StudyID*ExptID 1 StudyID*EstimateID 1/cl divisor=3 alpha=0.1; *check iso is a postivie then ave covar is a -103.1;

estimate "Hyper-Water" TimeBin*Treat 1 0 0 -1 1 0 0 -1 1 0 0 -1 TimeBin*Treat*DrinkOsM 98.6 0 0 0 98.6 0 0 0 98.6 0 0 0 TimeBin*Treat*FrDi 1 0 0 -1 1 0 0 -1 1 0 0 -1 TimeBin*Treat*FrPoly 1 0 0 -1 1 0 0 -1 1 0 0 -1|StudyID 1 StudyID*ExptID 1 StudyID*EstimateID 1/cl divisor=3 alpha=0.1;

estimate "Hypo-Water" TimeBin*Treat 0 1 0 -1 0 1 0 -1 0 1 0 -1 TimeBin*Treat*DrinkOsM 0 -108.2 0 0 0 -108.2 0 0 0 -108.2 0 0 TimeBin*Treat*FrDi 0 1 0 -1 0 1 0 -1 0 1 0 -1 TimeBin*Treat*FrPoly 0 1 0 -1 0 1 0 -1 0 1 0 -1|StudyID 1 StudyID*ExptID 1 StudyID*EstimateID 1/cl divisor=3 alpha=0.1;

*/

*** 16/4/20 to fix all data. then updat figures and data in figures... Fuck.;

/*

estimate "";

estimate "DrinkOsm Hyper LOW 350 mOSM";

estimate "TimeBinMeansOverall mean setting";*automatically adjusted for standardised covariates. If not standardised then need to put in the covariate;

estimate "Hyper" TimeBin*Treat 1 0 0 0 1 0 0 0 1 0 0 0 TimeBin*Treat*DrinkOsM 62.5 0 0 0 62.5 0 0 0 62.5 0 0 0/cl divisor=3 alpha=0.1;

estimate "Hypo" TimeBin*Treat 0 1 0 0 0 1 0 0 0 1 0 0 TimeBin*Treat*DrinkOsM 0 -108.2 0 0 0 -108.2 0 0 0 -108.2 0 0/cl divisor=3 alpha=0.1;

estimate "Iso" TimeBin*Treat 0 0 1 0 0 0 1 0 0 0 1 0 TimeBin*Treat*DrinkOsM 0 0 -3.2 0 0 0 -3.2 0 0 0 -3.2 0/cl divisor=3 alpha=0.1;

estimate "Water" TimeBin*Treat 0 0 0 1 0 0 0 1 0 0 0 1 TimeBin*Treat*DrinkOsM 0 0 0 0 0 0 0 0 0 0 0 0/cl divisor=3 alpha=0.1;

estimate "Hyper-iso" TimeBin*Treat 1 0 -1 0 1 0 -1 0 1 0 -1 0 TimeBin*Treat*DrinkOsM 62.5 0 3.2 0 62.5 0 3.2 0 62.5 0 3.2 0/cl divisor=3 alpha=0.1;

estimate "Hypo-iso" TimeBin*Treat 0 1 -1 0 0 1 -1 0 0 1 -1 0 TimeBin*Treat*DrinkOsM 0 -108.2 3.2 0 0 -108.2 3.2 0 0 -108.2 3.2 0/cl divisor=3 alpha=0.1;

estimate "Water-iso" TimeBin*Treat 0 0 -1 1 0 0 -1 1 0 0 -1 1 TimeBin*Treat*DrinkOsM 0 0 3.2 0 0 0 3.2 0 0 0 3.2 0/cl divisor=3 alpha=0.1;

estimate "Hyper-Hypo" TimeBin*Treat 1 -1 0 0 1 -1 0 0 1 -1 0 0 TimeBin*Treat*DrinkOsM 62.5 108.2 0 0 62.5 108.2 0 0 62.5 108.2 0 0/cl divisor=3 alpha=0.1; *check iso is a postivie then ave covar is a -103.1;

estimate "Hyper-Water" TimeBin*Treat 1 0 0 -1 1 0 0 -1 1 0 0 -1 TimeBin*Treat*DrinkOsM 62.5 0 0 0 62.5 0 0 0 62.5 0 0 0/cl divisor=3 alpha=0.1;

estimate "Hypo-Water" TimeBin*Treat 0 1 0 -1 0 1 0 -1 0 1 0 -1 TimeBin*Treat*DrinkOsM 0 -108.2 0 0 0 -108.2 0 0 0 -108.2 0 0/cl divisor=3 alpha=0.1;

***editing up to here. 400 600 1200 - V high;

estimate "";

estimate "DrinkOsm Hyper HIGH 500 mOSM";

estimate "TimeBinMeansOverall mean setting";*automatically adjusted for standardised covariates. If not standardised then need to put in the covariate;

estimate "Hyper" TimeBin*Treat 1 0 0 0 1 0 0 0 1 0 0 0 TimeBin*Treat*DrinkOsM 212.5 0 0 0 212.5 0 0 0 212.5 0 0 0/cl divisor=3 alpha=0.1;

estimate "Hypo" TimeBin*Treat 0 1 0 0 0 1 0 0 0 1 0 0 TimeBin*Treat*DrinkOsM 0 -108.2 0 0 0 -108.2 0 0 0 -108.2 0 0/cl divisor=3 alpha=0.1;

estimate "Iso" TimeBin*Treat 0 0 1 0 0 0 1 0 0 0 1 0 TimeBin*Treat*DrinkOsM 0 0 -3.2 0 0 0 -3.2 0 0 0 -3.2 0/cl divisor=3 alpha=0.1;

estimate "Water" TimeBin*Treat 0 0 0 1 0 0 0 1 0 0 0 1 TimeBin*Treat*DrinkOsM 0 0 0 0 0 0 0 0 0 0 0 0/cl divisor=3 alpha=0.1;

estimate "Hyper-iso" TimeBin*Treat 1 0 -1 0 1 0 -1 0 1 0 -1 0 TimeBin*Treat*DrinkOsM 212.5 0 3.2 0 212.5 0 3.2 0 212.5 0 3.2 0/cl divisor=3 alpha=0.1;

estimate "Hypo-iso" TimeBin*Treat 0 1 -1 0 0 1 -1 0 0 1 -1 0 TimeBin*Treat*DrinkOsM 0 -108.2 3.2 0 0 -108.2 3.2 0 0 -108.2 3.2 0/cl divisor=3 alpha=0.1;

estimate "Water-iso" TimeBin*Treat 0 0 -1 1 0 0 -1 1 0 0 -1 1 TimeBin*Treat*DrinkOsM 0 0 3.2 0 0 0 3.2 0 0 0 3.2 0/cl divisor=3 alpha=0.1;

estimate "Hyper-Hypo" TimeBin*Treat 1 -1 0 0 1 -1 0 0 1 -1 0 0 TimeBin*Treat*DrinkOsM 212.5 108.2 0 0 212.5 108.2 0 0 212.5 108.2 0 0/cl divisor=3 alpha=0.1; *check iso is a postivie then ave covar is a -103.1;

estimate "Hyper-Water" TimeBin*Treat 1 0 0 -1 1 0 0 -1 1 0 0 -1 TimeBin*Treat*DrinkOsM 212.5 0 0 0 212.5 0 0 0 212.5 0 0 0/cl divisor=3 alpha=0.1;

estimate "Hypo-Water" TimeBin*Treat 0 1 0 -1 0 1 0 -1 0 1 0 -1 TimeBin*Treat*DrinkOsM 0 -108.2 0 0 0 -108.2 0 0 0 -108.2 0 0/cl divisor=3 alpha=0.1;

estimate "";

estimate "DrinkOsm Hyper HIGH 800 mOSM";

estimate "TimeBinMeansOverall mean setting";*automatically adjusted for standardised covariates. If not standardised then need to put in the covariate;

estimate "Hyper" TimeBin*Treat 1 0 0 0 1 0 0 0 1 0 0 0 TimeBin*Treat*DrinkOsM 512.5 0 0 0 512.5 0 0 0 512.5 0 0 0/cl divisor=3 alpha=0.1;

estimate "Hypo" TimeBin*Treat 0 1 0 0 0 1 0 0 0 1 0 0 TimeBin*Treat*DrinkOsM 0 -108.2 0 0 0 -108.2 0 0 0 -108.2 0 0/cl divisor=3 alpha=0.1;

estimate "Iso" TimeBin*Treat 0 0 1 0 0 0 1 0 0 0 1 0 TimeBin*Treat*DrinkOsM 0 0 -3.2 0 0 0 -3.2 0 0 0 -3.2 0/cl divisor=3 alpha=0.1;

estimate "Water" TimeBin*Treat 0 0 0 1 0 0 0 1 0 0 0 1 TimeBin*Treat*DrinkOsM 0 0 0 0 0 0 0 0 0 0 0 0/cl divisor=3 alpha=0.1;

estimate "Hyper-iso" TimeBin*Treat 1 0 -1 0 1 0 -1 0 1 0 -1 0 TimeBin*Treat*DrinkOsM 512.5 0 3.2 0 512.5 0 3.2 0 512.5 0 3.2 0/cl divisor=3 alpha=0.1;

estimate "Hypo-iso" TimeBin*Treat 0 1 -1 0 0 1 -1 0 0 1 -1 0 TimeBin*Treat*DrinkOsM 0 -108.2 3.2 0 0 -108.2 3.2 0 0 -108.2 3.2 0/cl divisor=3 alpha=0.1;

estimate "Water-iso" TimeBin*Treat 0 0 -1 1 0 0 -1 1 0 0 -1 1 TimeBin*Treat*DrinkOsM 0 0 3.2 0 0 0 3.2 0 0 0 3.2 0/cl divisor=3 alpha=0.1;

estimate "Hyper-Hypo" TimeBin*Treat 1 -1 0 0 1 -1 0 0 1 -1 0 0 TimeBin*Treat*DrinkOsM 512.5 108.2 0 0 512.5 108.2 0 0 512.5 108.2 0 0/cl divisor=3 alpha=0.1; *check iso is a postivie then ave covar is a -103.1;

estimate "Hyper-Water" TimeBin*Treat 1 0 0 -1 1 0 0 -1 1 0 0 -1 TimeBin*Treat*DrinkOsM 512.5 0 0 0 512.5 0 0 0 512.5 0 0 0/cl divisor=3 alpha=0.1;

estimate "Hypo-Water" TimeBin*Treat 0 1 0 -1 0 1 0 -1 0 1 0 -1 TimeBin*Treat*DrinkOsM 0 -108.2 0 0 0 -108.2 0 0 0 -108.2 0 0/cl divisor=3 alpha=0.1;

estimate "";

estimate "DrinkOsm Hypo LOW 108.2 mOSM";

estimate "TimeBinMeansOverall mean setting";*automatically adjusted for standardised covariates. If not standardised then need to put in the covariate;

estimate "Hyper" TimeBin*Treat 1 0 0 0 1 0 0 0 1 0 0 0 TimeBin*Treat*DrinkOsM 98.6 0 0 0 98.6 0 0 0 98.6 0 0 0/cl divisor=3 alpha=0.1;

estimate "Hypo" TimeBin*Treat 0 1 0 0 0 1 0 0 0 1 0 0 TimeBin*Treat*DrinkOsM 0 -177.5 0 0 0 -177.5 0 0 0 -177.5 0 0/cl divisor=3 alpha=0.1;

estimate "Iso" TimeBin*Treat 0 0 1 0 0 0 1 0 0 0 1 0 TimeBin*Treat*DrinkOsM 0 0 -3.2 0 0 0 -3.2 0 0 0 -3.2 0/cl divisor=3 alpha=0.1;

estimate "Water" TimeBin*Treat 0 0 0 1 0 0 0 1 0 0 0 1 TimeBin*Treat*DrinkOsM 0 0 0 0 0 0 0 0 0 0 0 0/cl divisor=3 alpha=0.1;

estimate "Hyper-iso" TimeBin*Treat 1 0 -1 0 1 0 -1 0 1 0 -1 0 TimeBin*Treat*DrinkOsM 98.6 0 3.2 0 98.6 0 3.2 0 98.6 0 3.2 0/cl divisor=3 alpha=0.1;

estimate "Hypo-iso" TimeBin*Treat 0 1 -1 0 0 1 -1 0 0 1 -1 0 TimeBin*Treat*DrinkOsM 0 -177.5 3.2 0 0 -177.5 3.2 0 0 -177.5 3.2 0/cl divisor=3 alpha=0.1;

estimate "Water-iso" TimeBin*Treat 0 0 -1 1 0 0 -1 1 0 0 -1 1 TimeBin*Treat*DrinkOsM 0 0 3.2 0 0 0 3.2 0 0 0 3.2 0/cl divisor=3 alpha=0.1;

estimate "Hyper-Hypo" TimeBin*Treat 1 -1 0 0 1 -1 0 0 1 -1 0 0 TimeBin*Treat*DrinkOsM 98.6 177.5 0 0 98.6 177.5 0 0 98.6 177.5 0 0/cl divisor=3 alpha=0.1; *check iso is a postivie then ave covar is a -103.1;

estimate "Hyper-Water" TimeBin*Treat 1 0 0 -1 1 0 0 -1 1 0 0 -1 TimeBin*Treat*DrinkOsM 98.6 0 0 0 98.6 0 0 0 98.6 0 0 0/cl divisor=3 alpha=0.1;

estimate "Hypo-Water" TimeBin*Treat 0 1 0 -1 0 1 0 -1 0 1 0 -1 TimeBin*Treat*DrinkOsM 0 -177.5 0 0 0 -177.5 0 0 0 -177.5 0 0/cl divisor=3 alpha=0.1;

***editing up to here. 400 600 1200 - V high;

estimate "";

estimate "DrinkOsm Hypo HIGH 220 mOSM";

estimate "TimeBinMeansOverall mean setting";*automatically adjusted for standardised covariates. If not standardised then need to put in the covariate;

estimate "Hyper" TimeBin*Treat 1 0 0 0 1 0 0 0 1 0 0 0 TimeBin*Treat*DrinkOsM 97.7 0 0 0 97.7 0 0 0 97.7 0 0 0/cl divisor=3 alpha=0.1;

estimate "Hypo" TimeBin*Treat 0 1 0 0 0 1 0 0 0 1 0 0 TimeBin*Treat*DrinkOsM 0 -67.5 0 0 0 -67.5 0 0 0 -67.5 0 0/cl divisor=3 alpha=0.1;

estimate "Iso" TimeBin*Treat 0 0 1 0 0 0 1 0 0 0 1 0 TimeBin*Treat*DrinkOsM 0 0 -3.2 0 0 0 -3.2 0 0 0 -3.2 0/cl divisor=3 alpha=0.1;

estimate "Water" TimeBin*Treat 0 0 0 1 0 0 0 1 0 0 0 1 TimeBin*Treat*DrinkOsM 0 0 0 0 0 0 0 0 0 0 0 0/cl divisor=3 alpha=0.1;

estimate "Hyper-iso" TimeBin*Treat 1 0 -1 0 1 0 -1 0 1 0 -1 0 TimeBin*Treat*DrinkOsM 97.7 0 3.2 0 97.7 0 3.2 0 97.7 0 3.2 0/cl divisor=3 alpha=0.1;

estimate "Hypo-iso" TimeBin*Treat 0 1 -1 0 0 1 -1 0 0 1 -1 0 TimeBin*Treat*DrinkOsM 0 -67.5 3.2 0 0 -67.5 3.2 0 0 -67.5 3.2 0/cl divisor=3 alpha=0.1;

estimate "Water-iso" TimeBin*Treat 0 0 -1 1 0 0 -1 1 0 0 -1 1 TimeBin*Treat*DrinkOsM 0 0 3.2 0 0 0 3.2 0 0 0 3.2 0/cl divisor=3 alpha=0.1;

estimate "Hyper-Hypo" TimeBin*Treat 1 -1 0 0 1 -1 0 0 1 -1 0 0 TimeBin*Treat*DrinkOsM 97.7 67.5 0 0 97.7 67.5 0 0 97.7 67.5 0 0/cl divisor=3 alpha=0.1; *check iso is a postivie then ave covar is a -103.1;

estimate "Hyper-Water" TimeBin*Treat 1 0 0 -1 1 0 0 -1 1 0 0 -1 TimeBin*Treat*DrinkOsM 97.7 0 0 0 97.7 0 0 0 97.7 0 0 0/cl divisor=3 alpha=0.1;

estimate "Hypo-Water" TimeBin*Treat 0 1 0 -1 0 1 0 -1 0 1 0 -1 TimeBin*Treat*DrinkOsM 0 -67.5 0 0 0 -67.5 0 0 0 -67.5 0 0/cl divisor=3 alpha=0.1;

estimate "";

estimate "DrinkOsm Hypo HIGH 260 mOSM";

estimate "TimeBinMeansOverall mean setting";*automatically adjusted for standardised covariates. If not standardised then need to put in the covariate;

estimate "Hyper" TimeBin*Treat 1 0 0 0 1 0 0 0 1 0 0 0 TimeBin*Treat*DrinkOsM 97.7 0 0 0 97.7 0 0 0 97.7 0 0 0/cl divisor=3 alpha=0.1;

estimate "Hypo" TimeBin*Treat 0 1 0 0 0 1 0 0 0 1 0 0 TimeBin*Treat*DrinkOsM 0 -27.5 0 0 0 -27.5 0 0 0 -27.5 0 0/cl divisor=3 alpha=0.1;

estimate "Iso" TimeBin*Treat 0 0 1 0 0 0 1 0 0 0 1 0 TimeBin*Treat*DrinkOsM 0 0 -3.2 0 0 0 -3.2 0 0 0 -3.2 0/cl divisor=3 alpha=0.1;

estimate "Water" TimeBin*Treat 0 0 0 1 0 0 0 1 0 0 0 1 TimeBin*Treat*DrinkOsM 0 0 0 0 0 0 0 0 0 0 0 0/cl divisor=3 alpha=0.1;

estimate "Hyper-iso" TimeBin*Treat 1 0 -1 0 1 0 -1 0 1 0 -1 0 TimeBin*Treat*DrinkOsM 97.7 0 3.2 0 97.7 0 3.2 0 97.7 0 3.2 0/cl divisor=3 alpha=0.1;

estimate "Hypo-iso" TimeBin*Treat 0 1 -1 0 0 1 -1 0 0 1 -1 0 TimeBin*Treat*DrinkOsM 0 -27.5 3.2 0 0 -27.5 3.2 0 0 -27.5 3.2 0/cl divisor=3 alpha=0.1;

estimate "Water-iso" TimeBin*Treat 0 0 -1 1 0 0 -1 1 0 0 -1 1 TimeBin*Treat*DrinkOsM 0 0 3.2 0 0 0 3.2 0 0 0 3.2 0/cl divisor=3 alpha=0.1;

estimate "Hyper-Hypo" TimeBin*Treat 1 -1 0 0 1 -1 0 0 1 -1 0 0 TimeBin*Treat*DrinkOsM 97.7 27.5 0 0 97.7 27.5 0 0 97.7 27.5 0 0/cl divisor=3 alpha=0.1; *check iso is a postivie then ave covar is a -103.1;

estimate "Hyper-Water" TimeBin*Treat 1 0 0 -1 1 0 0 -1 1 0 0 -1 TimeBin*Treat*DrinkOsM 97.7 0 0 0 97.7 0 0 0 97.7 0 0 0/cl divisor=3 alpha=0.1;

estimate "Hypo-Water" TimeBin*Treat 0 1 0 -1 0 1 0 -1 0 1 0 -1 TimeBin*Treat*DrinkOsM 0 -27.5 0 0 0 -27.5 0 0 0 -27.5 0 0/cl divisor=3 alpha=0.1;

*/

ods output covparms=cov;

ods output residualsdPV=residualsdPV;

ods output lsmeans=lsm;

*ods output diffs=lsmdif;

ods output estimates=est;

ods output solutionf=solf;

*title1 "META effect all contrasts fold hypo-isot at Mizone osmol 230 mOSm vs isotonic 287.5 mOsm time 57.2 min";

**run**;

ods listing;

**data** covbtwn;

set cov;

SE2=stderr****2**;

if covparm ne "Residual";

**proc** **means** noprint;

var estimate se2;

output out=covbtwn1 sum=;

**data** covbtwn2;

covparm="Btwn settings";

set covbtwn1;

StdErr=sqrt(se2);

*alpha=&alpha;

lower=estimate+probit(alpha/**2**)*StdErr;*probit not working;

upper=estimate-probit(alpha/**2**)*StdErr;

Zvalue=estimate/StdErr;

**run**;

**data** cov1;

set cov covbtwn2;

if covparm ne "Residual";

DegFree=**2***Zvalue****2**;

a=**1**;b=**1**;c=**1**;

if estimate<**0** then a=-**1**;

if lower<**0** then b=-**1**;

if upper<**0** then c=-**1**;

SD=a*sqrt(a*estimate);

lower=b*sqrt(b*lower);

upper=c*sqrt(c*upper);

array r SD lower upper;

*if &logflag=1 then do over r;

*r=100*exp(r/100)-100;

*end;

*CLtd=sqrt(upper/lower);

CLpm=(upper-lower)/**2**;

Units="Raw";

*if &logflag then Units="%";

title2 "Random effects as SD";

options ls=**100**;

**proc** **print** data=cov1 noobs;

var covparm Units SD CLpm lower upper alpha DegFree;

format SD lower upper CLpm **5.2** DegFree **5.**;

**run**;

title2 "Decisions for random effects";

**data** cov1;

set cov covbtwn2;

if covparm ne "Residual";

DF=**999**;

*MagniThresh=abs(&MagniThresh)/2;

MagniThresh=abs(**0.2*****2.62**)/**2**;*0.2 times baseline SD for dPV as a percent of mean;

*if &logflag then Magnithresh=100*exp(log(1+abs(&magnithresh)/100)/2)-100;

ProbPos=(**1**-ProbT(-(estimate-abs(MagniThresh))/StdErr,DF));

ProbNeg=ProbT(-(estimate+abs(MagniThresh))/StdErr,DF);

*if &LogFlag=1 then do;

if Magnithresh>**0** then do;

ProbPos=(**1**-ProbT(-(estimate-(MagniThresh))/StdErr,DF));

ProbNeg=ProbT(-(estimate+(MagniThresh))/StdErr,DF);

end;

else do;

ProbPos=(**1**-ProbT(-(estimate+(MagniThresh))/StdErr,DF));

ProbNeg=ProbT(-(estimate-(MagniThresh))/StdErr,DF);

end;

*end;

ProbTriv=**1**-ProbPos-ProbNeg;

ORPosNeg=ProfPos/(**1**-ProbPos)/(ProbNeg/(**1**-ProbNeg));

ORNegPos=**1**/ORPosNeg;

ClinFlag=**1**; *want all inferences to be clinical initially;

*if index(label,"2SD") then ClinFlag=0; *covariates definitely need to be non-clinical;

*mechanistic inferences as frequentist;

*ClinFlag=0;

ClearOrNot="unclear";

Strgth="";

Magni="";

ORPosNeg=**.**; ORNegPos=**.**;

if ProbNeg<**0.05** or ProbPos<**0.05** then ClearOrNot="@0.1% ";

if ProbNeg<**0.005** or ProbPos<**0.005** then ClearOrNot="@0.01% ";

if ClearOrNot ne "unclear" then do;

Magni="+ive ";

if estimate<**0** then Magni="-ive ";

if ProbPos>**0.05** or ProbNeg>**0.05** then Strgth="improbable";

if ProbPos>**0.25** or ProbNeg>**0.25** then Strgth="about as likely as not";

if ProbPos>**0.75** or ProbNeg>**0.75** then Strgth="likely ";

if ProbPos>**0.95** or ProbNeg>**0.95** then Strgth="v.likely ";

if ProbPos>**0.995** or ProbNeg>**0.995** then Strgth="virtually_certain";

end;

if ClearOrNot ne "unclear" and ProbTriv>**75** then do;

Magni="triv.";

Strgth="weak ";

if ProbTriv>**0.95** then Prob="v.likely ";

if ProbTriv>**0.995** then Prob="virtually_certain";

end;

*end;

output;

**data** cov2;

set cov1;

a=**1**;b=**1**;c=**1**;

if estimate<**0** then a=-**1**;

if lower<**0** then b=-**1**;

if upper<**0** then c=-**1**;

SD=a*sqrt(a*estimate);

lower=b*sqrt(b*lower);

upper=c*sqrt(c*upper);

DegFree=**2***Zvalue****2**;

array r SD lower upper;

/*

if &logflag=1 then do over r;

r=100*exp(r/100)-100;

end;

*/

*CLtd=sqrt(upper/lower);

CLpm=(upper-lower)/**2**;

Units="Raw";

*if &logflag then Units="%";

**run**;

options ls=**120** ps=**80**;

**proc** **print** data=cov2 noobs;

*where clinflag=0;

var covparm Units SD CLpm lower upper alpha

MagniThresh ProbNeg ProbTriv ProbPos Strgth Magni ClearOrNot;

format SD CLpm lower upper MagniThresh **5.2** Probt best5. ProbNeg ProbTriv ProbPos **5.1** DegFree **5.0**;

title3 "Non-clinical inferences for SD representing random effects";

title4 "Magnithresh is half the smallest difference for means";

**run**;

********************;

**data** est1;

set est;

*Units="Raw dPV";

CLpm=(Upper+Lower)/**2**;/*

*if &logflag then Units="factor";

array a estimate lower upper;

if &logflag then do over a;

a=exp(a/100); *all are factors;

end;

CLtd=sqrt(Upper/Lower);

if estimate=1 then do; estimate=.; Units=""; end;*/

**run**;

/*

title2 "Fixed-effect estimates";

proc print noobs data=est1;

var Label Estimate CLpm Lower Upper alpha;

format Estimate Lower Upper CLpm 5.2;

run;

*/

*magnitude-based inferences for fixed effects;

*clin and non-clin MBIs are output and listed separately;

title3 " Decisions for fixed effects";

**data** est2;

set est1;

length Strgth $ **10** Magni $ **4**;

MagniThresh=**0.2*****2.62**; *%SD baseline PV from young mean 2.03% Davy, K. P., & Seals, D. R. (1994). Total blood volume in healthy young and older men. J Appl Physiol (1985), 76(5), 2059-2062. doi: 10.1152/jappl.1994.76.5.2059

age 40-49 yr sample 3.2% from Yiengst, M. J., & Shock, N. W. (1962). Blood and plasma volume in adult males. J Appl Physiol, 17, 195-198. doi: 10.1152/jappl.1962.17.2.195;

/*

MagniThresh=0.2*2.6135; *2.6135

Hyper 2.68787

Hypo 3.07517

Iso 3.04675

Water 1.16584;

*if _n_=1 then set covarSD(keep=stddev);

*/

*SD=stddev;

Cn=**1**-**3**/(**4***df-**1**); *bias is due to uncertainty in SD;

ES=Cn*estimate/(MagniThresh/**0.2**);*SD;

*NCparm=tValue*sqrt((DFdenom+1)/(DFnum+1));

*alpha=0.1;

*CL_pc=alpha*100;

ESlower=ES/tValue*TINV(alpha/**2**,df,tvalue);

ESupper=ES/tValue*TINV(**1**-alpha/**2**,df,tvalue);

*Changed from Changes to Frequentist Probabilities by deleting 100*;

ProbPos=(**1**-ProbT(-(estimate-abs(MagniThresh))/StdErr,DF));

ProbNeg=ProbT(-(estimate+abs(MagniThresh))/StdErr,DF);

*if &LogFlag=1 then do;

if Magnithresh>**0** then do;

ProbPos=(**1**-ProbT(-(estimate-(MagniThresh))/StdErr,DF));

ProbNeg=ProbT(-(estimate+(MagniThresh))/StdErr,DF);

end;

else do;

ProbPos=(**1**-ProbT(-(estimate+(MagniThresh))/StdErr,DF));

ProbNeg=ProbT(-(estimate-(MagniThresh))/StdErr,DF);

end;

*end;

ProbTriv=**1**-ProbPos-ProbNeg;

ORPosNeg=ProfPos/(**1**-ProbPos)/(ProbNeg/(**1**-ProbNeg));

ORNegPos=**1**/ORPosNeg;

ClinFlag=**1**; *want all inferences to be clinical initially;

*if index(label,"2SD") then ClinFlag=0; *covariates definitely need to be non-clinical;

/*

*clinical inferences;

if clinflag then do;

ChPos=ProbPos; ChNeg=ProbNeg;

if MagniThresh<0 then do;

ChPos=ProbNeg; ChNeg=ProbPos;

end;

Prob=""; Magni=""; ClearOrNot="unclear";

if ChNeg<0.5 then do;

ClearOrNot="@25/.5%";

if ChNeg<0.1 then ClearOrNot="@5/.1% ";

if ProbTriv>25 then Magni="triv";

if ChPos>25 then Magni="bene";

Prob=“about as likely as not”;

if ChPos>75 or ProbTriv>75 then Prob="likely ";

if ChPos>95 or ProbTriv>95 then Prob="v.likely";

if ChPos>99.5 or ProbTriv>99.5 then Prob=“virtually certain”;

end;

else do; *i.e., ChNeg>0.5;

if ChPos<25 then do;

ClearOrNot="@25/.5%";

if ChPos<5 then ClearOrNot="@5/.1% ";

if ProbTriv>25 then Magni="triv";

if ChNeg>25 then Magni="harm";

Prob=“about as likely as not”;

if ChNeg>75 or ProbTriv>75 then Prob="likely ";

if ChNeg>95 or ProbTriv>95 then Prob="v.likely";

if ChNeg>99.5 or ProbTriv>99.5 then Prob=“virtually certain”;

end;

end;

if ClearOrNot="unclear" and

(MagniThresh>0 and ORPosNeg>25/75/(0.5/99.5) or MagniThresh<0 and ORNegPos>25/75/(0.5/99.5))

then do;

ClearOrNot="OR>66.3";

Magni="bene";

if ChPos>25 then Prob=“about as likely as not”;

if ChPos>75 then Prob="likely ";

if ChPos>95 then Prob="v.likely";

if ChPos>99.5 then Prob=“virtually certain”;

end;

output;

end;

*/

*mechanistic inferences as frequentist;

*ClinFlag=0;

ClearOrNot="unclear";

Strgth="";

Magni="";

ORPosNeg=**.**; ORNegPos=**.**;

if ProbNeg<**0.05** or ProbPos<**0.05** then ClearOrNot="@0.1% ";

if ProbNeg<**0.005** or ProbPos<**0.005** then ClearOrNot="@0.01% ";

if ClearOrNot ne "unclear" then do;

Magni="+ive ";

if estimate<**0** then Magni="-ive ";

if ProbPos>**0.05** or ProbNeg>**0.05** then Strgth="improbable";

if ProbPos>**0.25** or ProbNeg>**0.25** then Strgth="about as likely as not";

if ProbPos>**0.75** or ProbNeg>**0.75** then Strgth="likely ";

if ProbPos>**0.95** or ProbNeg>**0.95** then Strgth="v.likely ";

if ProbPos>**0.995** or ProbNeg>**0.995** then Strgth="virtually_certain";

end;

if ClearOrNot ne "unclear" and ProbTriv>**75** then do;

Magni="triv.";

Strgth="weak ";

if ProbTriv>**0.95** then Prob="v.likely ";

if ProbTriv>**0.995** then Prob="virtually_certain";

end;

*end;

output;

/*

*mechanistic inferences;

*ClinFlag=0;

ClearOrNot="unclear";

Prob="";

Magni="";

ORPosNeg=.; ORNegPos=.;

if ProbNeg<0.05 or ProbPos<0.05 then ClearOrNot="@90% ";

if ProbNeg<0.005 or ProbPos<0.005 then ClearOrNot="@99% ";

if ClearOrNot ne "unclear" then do;

Magni="+ive ";

if estimate<0 then Magni="-ive ";

if ProbPos>0.05 or ProbNeg>0.05 then Prob="unlikely";

if ProbPos>0.25 or ProbNeg>0.25 then Prob=“about as likely as not”;

if ProbPos>0.75 or ProbNeg>0.75 then Prob="likely ";

if ProbPos>0.95 or ProbNeg>0.95 then Prob="v.likely";

if ProbPos>0.995 or ProbNeg>0.995 then Prob=“virtually certain”;

end;

if ClearOrNot ne "unclear" and ProbTriv>75 then do;

Magni="triv.";

Prob="likely ";

if ProbTriv>0.95 then Prob="v.likely";

if ProbTriv>0.995 then Prob=“virtually certain”;

end;

*end;

output;

*/

/*

data est2;

Units="Raw ";

if &logflag then Units="factor";

set est1;

if estimate=0 then do;

estimate=.; magnithresh=.; magni=""; clearornot=""; Prob=""; Units="";

end;

array a estimate lower upper;

if &logflag then do over a;

a=exp(a/100); *all are factors;

end;

CLtd=sqrt(Upper/lower);

rename df=DegFree;

run;

*/

**data** est3;

set est2;

*if substr(Label,1,1)="." then delete;

*if substr(Label,1,1)="*" then Label=substr(Label,2);

*options ls=135 ps=80;

title5 "Non-clinical inferences";

title6 "Magnithresh is smallest change";

**proc** **print** data=est3 noobs;

*where clinflag=0;

var label estimate lower upper ES ESlower ESupper alpha DF

MagniThresh ProbNeg ProbTriv ProbPos Probt Strgth Magni ClearOrNot;

format estimate CLpm lower upper MagniThresh **5.1** ES ESlower ESupper **5.2** Probt best5. ProbNeg ProbTriv

ProbPos **5.4** DF **5.0**;

**run**;

**C.** **SAS code for effective osmolality analysis mixed model**

filename in "C:\Users\dsrowlan\OneDrive - Massey University\

Documents\Massey\Massey_2020\Research\Frucor_Hydration Review\

ECSS_2018_Manuscript meta-analysis\New_Dec 2019\

Working SAS_Feb 2020\Dat12wCHO_2.txt";

options linesize=**150** pagesize=**500**;

**data** dat12;

infile in missover firstobs=**2** delimiter='09'x;

*RowNo=_n_;

length Study $ **30** ; *length Strgth $ 10 Magni $ 4;

input StudyID ExptID EstimateID Study $ Source $ contrast

constrastn studytype $ Treat $ subjectn ex_mode $ ex_intensity_PCVOtwomax

Votwomax_ml_kg_min Bodyweight Votwomax_l_min metabolicrate

thermal_conditions $ TempC Humidity time drink_vol

Total_fluid avg_ingestion_rate drinkOsM_measured units $

drinkOsM_calc binOsm SD dPV SEM PV_Method $ logSD SElogSD InvSE2

folddPV logdPV DrinkOsM Pred StdErrPred DF Alpha Lower Upper

Resid Var SE WiRE meanhumidity meanTempC tempF heatindex

TimeBin $ CHOConc_Tot__gPC Frucconc_gPC glucconc_gPC sucrconc_gPC

maltoseconc_gPC Polyconc_gPC FrDi FrPoly Effective_Frucratio NamEq

KmEq MgmEq CamEq;

**run**;

**Data** dat13;

set dat12;

mEqtot=(**2***NamEq)+(**2***KmEq)+(MgmEq***3**)+(CamEq***3**);

DrinkOsMAdj=((CHOConc_Tot__gPC/**180*****1000**/**0.1**)+mEqtot+**10**)-**287.5**;*10 to allow for flavourings in drinks and background salts in water;

if Treat="Water" then DrinkOsMAdj=**0**;

**run**;

title8 "Raw Means ALL for covariates";

**proc** **means** data=dat13 maxdec=**1**;

var heatindex TempC Humidity avg_ingestion_rate metabolicrate drinkOsM CHOConc_Tot__gPC Frucconc_gPC

glucconc_gPC sucrconc_gPC maltoseconc_gPC Polyconc_gPC FrDi FrPoly Effective_Frucratio mEqtot DrinkOsMAdj;*drinkOsM;

*by Treat;

output out=meansallcovariates mean=mean stddev=SD min=min max=max;

**run**;

**proc** **sort** data=dat13;

by Treat;

**run**;

title9 "Raw Means BY TREAT for covariates";

**proc** **means** data=dat13 maxdec=**1**;

var heatindex TempC Humidity avg_ingestion_rate metabolicrate drinkOsM CHOConc_Tot__gPC Frucconc_gPC

glucconc_gPC sucrconc_gPC maltoseconc_gPC Polyconc_gPC FrDi FrPoly Effective_Frucratio mEqtot DrinkOsMAdj;*drinkOsM;

by Treat;

output out=meanstreatcovariates mean=mean stddev=SD min=min max=max;

**run**;

**proc** **standard** data=dat13 out=dat11 mean=**0** std=**0.5**;

var heatindex TempC Humidity avg_ingestion_rate metabolicrate drinkOsM CHOConc_Tot__gPC

Frucconc_gPC glucconc_gPC sucrconc_gPC maltoseconc_gPC Polyconc_gPC FrDi FrPoly Effective_Frucratio mEqtot;*for the coefficients, 1 gives 2SD; *drinkOsM not standarised;

*by ex_type;

**run**;

**Treat: hyper hypo iso water;;

ods listing close;

**proc** **mixed** covtest data=dat13 cl alpha=**0.1** nobound CONVH=**1E-6** convf=**1E-6**;*nobound;*forcing it to converge at 6 dp;

class StudyID TimeBin ExptID Treat EstimateID ex_mode;

weight WiRE;

*model dPV=TimeBin*treat/noint cl s DDFM=satalpha=0.1;

*standarised covariates for later avg_ingestion_rate*Treat heatindex*Treat metabolicrate*Treat;

*modifying and mediating covariates; *heatindex was found not to be an important modifer, so not continued with;

*model dPV=TimeBin*Treat TimeBin*Treat*DrinkOsM TimeBin*Treat*avg_ingestion_rate TimeBin*Treat*avg_ingestion_rate TimeBin*Treat*heatindex/noint cl DDFM=sat outp=pred outpm=predm residual alpha=0.1; *model to generate residuals for t vs SE plot for assymetry. When running turn off random effects;

*model dPV=TimeBin*Treat TimeBin*Treat*DrinkOsM TimeBin*Treat*metabolicrate/noint cl DDFM=sat outp=pred outpm=predm residual alpha=0.1;

*model dPV=TimeBin*Treat TimeBin*Treat*DrinkOsM TimeBin*Treat*avg_ingestion_rate/noint cl DDFM=sat outp=pred outpm=predm residual alpha=0.1;

*model dPV=TimeBin*Treat TimeBin*Treat*DrinkOsM TimeBin*Treat*heatindex/noint cl DDFM=sat outp=pred outpm=predm residual alpha=0.1;

*model dPV=TimeBin*Treat TimeBin*Treat*DrinkOsM/noint cl s DDFM=sat outp=pred outpm=predm residual alpha=0.1;

*model dPV=TimeBin*Treat TimeBin*Treat*heatindex TimeBin*Treat*metabolicrate TimeBin*Treat*avg_ingestion_rate/noint cl s DDFM=sat outp=pred outpm=predm alpha=0.1;

*model dPV=TimeBin*Treat TimeBin*Treat*DrinkOsM TimeBin*Treat*metabolicrate TimeBin*Treat*avg_ingestion_rate/noint cl DDFM=sat outp=pred outpm=predm alpha=0.1;

*2. to estimate the raw, at gut wall, CHO effects, run model but with Effective Functional OsM, adjused for [CHO] and F:G;

* TimeBin*Treat*CHOConc_Tot__gPC TimeBin*Treat*Effective_Frucratio;

model dPV=TimeBin*Treat TimeBin*Treat*DrinkOsMAdj

TimeBin*Treat*metabolicrate TimeBin*Treat*avg_ingestion_rate

/noint cl DDFM=sat outp=pred outpm=predm alpha=**0.1**;

*random int ExptID Timebin*Treat/subject=StudyID ;*Treat*StudyID, Int is StudyID;

random StudyID StudyID*ExptID StudyID*EstimateID/;

Parms **5** **1** **1** **1**/hold=**4**;

*clamping residual covariance to 1 means the weighting factor is the random effect for each dependent variable. Residual can be negative variance in this instance;

*references: Cassar et al 2016 Human Reproduction, pp. 1–13, 2016 doi:10.1093/humrep/dew243;

*Weston 2014 Sports Med (2014) 44:1005–1017, DOI 10.1007/s40279-014-0180-z;

*Yang 2003 A review of random effects modelling in SAS (release 8.2);

****do for Osm e.g. 350 600 1200... 220 150. range of OsM generally2SD accros studies, but something sensible;

*****finsih updateing 2SD coeffecients:;

estimate " ";

estimate "modifying effect of covars on dPV";

estimate "after ajusting for CHO properties";

estimate "Effects of 2SD on dPV";

estimate "DrinkOsMAdj all" TimeBin*Treat*DrinkOsMAdj **462.6** **277.6** **97.6** **0** **462.6** **277.6** **97.6** **0** **462.6** **277.6** **97.6** **0**/cl divisor=**12** alpha=**0.1**;

estimate "DrinkOsMAdj hyper" TimeBin*Treat*DrinkOsMAdj **462.6** **0** **0** **0** **462.6** **0** **0** **0** **462.6** **0** **0** **0**/cl divisor=**3** alpha=**0.1**;

estimate "DrinkOsMAdj hypo" TimeBin*Treat*DrinkOsMAdj **0** **277.6** **0** **0** **0** **277.6** **0** **0** **0** **277.6** **0** **0**/cl divisor=**3** alpha=**0.1**;

estimate "DrinkOsMAdj iso" TimeBin*Treat*DrinkOsMAdj **0** **0** **97.6** **0** **0** **0** **97.6** **0** **0** **0** **97.6** **0**/cl divisor=**3** alpha=**0.1**;

estimate "DrinkOsMAdj water" TimeBin*Treat*DrinkOsMAdj **0** **0** **0** **0** **0** **0** **0** **0** **0** **0** **0** **0**/cl divisor=**3** alpha=**0.1**;

estimate "Timebin1. ";

estimate "DrinkOsMAdj all" TimeBin*Treat*DrinkOsMAdj **462.6** **277.6** **97.6** **0** **0** **0** **0** **0** **0** **0** **0** **0**/cl divisor=**4** alpha=**0.1**;

estimate "DrinkOsMAdj hyper" TimeBin*Treat*DrinkOsMAdj **462.6** **0** **0** **0** **0** **0** **0** **0** **0** **0** **0** **0**/cl divisor=**1** alpha=**0.1**;

estimate "DrinkOsMAdj hypo" TimeBin*Treat*DrinkOsMAdj **0** **277.6** **0** **0** **0** **0** **0** **0** **0** **0** **0**/cl divisor=**1** alpha=**0.1**;

estimate "DrinkOsMAdj iso" TimeBin*Treat*DrinkOsMAdj **0** **0** **97.6** **0** **0** **0** **0** **0** **0** **0** **0** **0** **0**/cl divisor=**1** alpha=**0.1**;

estimate "DrinkOsMAdj water" TimeBin*Treat*DrinkOsMAdj **0** **0** **0** **0** **0** **0** **0** **0** **0** **0** **0** **0**/cl divisor=**1** alpha=**0.1**;

estimate "Timebin2. ";

estimate "DrinkOsMAdj all" TimeBin*Treat*DrinkOsMAdj **0** **0** **0** **0** **462.6** **277.6** **97.6** **0** **0** **0** **0** **0**/cl divisor=**4** alpha=**0.1**;

estimate "DrinkOsMAdj hyper" TimeBin*Treat*DrinkOsMAdj **0** **0** **0** **0** **462.6** **0** **0** **0** **0** **0** **0** **0**/cl divisor=**1** alpha=**0.1**;

estimate "DrinkOsMAdj hypo" TimeBin*Treat*DrinkOsMAdj **0** **0** **0** **0** **0** **277.6** **0** **0** **0** **0** **0** **0**/cl divisor=**1** alpha=**0.1**;

estimate "DrinkOsMAdj iso" TimeBin*Treat*DrinkOsMAdj **0** **0** **0** **0** **0** **0** **97.6** **0** **0** **0** **0** **0**/cl divisor=**1** alpha=**0.1**;

estimate "DrinkOsMAdj water" TimeBin*Treat*DrinkOsMAdj **0** **0** **0** **0** **0** **0** **0** **0** **0** **0** **0** **0**/cl divisor=**1** alpha=**0.1**;

estimate "Timebin3. ";

estimate "DrinkOsMAdj all" TimeBin*Treat*DrinkOsMAdj **0** **0** **0** **0** **0** **0** **0** **0** **462.6** **277.6** **97.6** **0**/cl divisor=**4** alpha=**0.1**;

estimate "DrinkOsMAdj hyper" TimeBin*Treat*DrinkOsMAdj **0** **0** **0** **0** **0** **0** **0** **0** **462.6** **0** **0** **0**/cl divisor=**1** alpha=**0.1**;

estimate "DrinkOsMv hypo" TimeBin*Treat*DrinkOsMAdj **0** **0** **0** **0** **0** **0** **0** **0** **0** **277.6** **0** **0**/cl divisor=**1** alpha=**0.1**;

estimate "DrinkOsMAdj iso" TimeBin*Treat*DrinkOsMAdj **0** **0** **0** **0** **0** **0** **0** **0** **0** **0** **97.6** **0**/cl divisor=**1** alpha=**0.1**;

estimate "DrinkOsMAdj water" TimeBin*Treat*DrinkOsMAdj **0** **0** **0** **0** **0** **0** **0** **0** **0** **0** **0** **0**/cl divisor=**1** alpha=**0.1**;

estimate " ";

estimate "Effects of 2SD Metabolicrate";

estimate "Metabolicrate all" TimeBin*Treat*metabolicrate **1** **1** **1** **1** **1** **1** **1** **1** **1** **1** **1** **1**/cl divisor=**12** alpha=**0.1**;

estimate "Metabolicrate hyper" TimeBin*Treat*metabolicrate **1** **0** **0** **0** **1** **0** **0** **0** **1** **0** **0** **0**/cl divisor=**3** alpha=**0.1**;

estimate "Metabolicrate hypo" TimeBin*Treat*metabolicrate **0** **1** **0** **0** **0** **1** **0** **0** **0** **1** **0** **0**/cl divisor=**3** alpha=**0.1**;

estimate "Metabolicrate iso" TimeBin*Treat*metabolicrate **0** **0** **1** **0** **0** **0** **1** **0** **0** **0** **1** **0**/cl divisor=**3** alpha=**0.1**;

estimate "Metabolicrate water" TimeBin*Treat*metabolicrate **0** **0** **0** **1** **0** **0** **0** **1** **0** **0** **0** **1**/cl divisor=**3** alpha=**0.1**;

estimate "Timebin1. ";

estimate "Metabolicrate all" TimeBin*Treat*metabolicrate **1** **1** **1** **1** **0** **0** **0** **0** **0** **0** **0** **0**/cl divisor=**4** alpha=**0.1**;

estimate "Metabolicrate hyper" TimeBin*Treat*metabolicrate **1** **0** **0** **0** **0** **0** **0** **0** **0** **0** **0** **0**/cl divisor=**1** alpha=**0.1**;

estimate "Metabolicrate hypo" TimeBin*Treat*metabolicrate **0** **1** **0** **0** **0** **0** **0** **0** **0** **0** **0**/cl divisor=**1** alpha=**0.1**;

estimate "Metabolicrate iso" TimeBin*Treat*metabolicrate **0** **0** **1** **0** **0** **0** **0** **0** **0** **0** **0** **0** **0**/cl divisor=**1** alpha=**0.1**;

estimate "Metabolicrate water" TimeBin*Treat*metabolicrate **0** **0** **0** **1** **0** **0** **0** **0** **0** **0** **0** **0**/cl divisor=**1** alpha=**0.1**;

estimate "Timebin2. ";

estimate "Metabolicrate all" TimeBin*Treat*metabolicrate **0** **0** **0** **0** **1** **1** **1** **1** **0** **0** **0** **0**/cl divisor=**4** alpha=**0.1**;

estimate "Metabolicrate hyper" TimeBin*Treat*metabolicrate **0** **0** **0** **0** **1** **0** **0** **0** **0** **0** **0** **0**/cl divisor=**1** alpha=**0.1**;

estimate "Metabolicrate hypo" TimeBin*Treat*metabolicrate **0** **0** **0** **0** **0** **1** **0** **0** **0** **0** **0** **0**/cl divisor=**1** alpha=**0.1**;

estimate "Metabolicrate iso" TimeBin*Treat*metabolicrate **0** **0** **0** **0** **0** **0** **1** **0** **0** **0** **0** **0**/cl divisor=**1** alpha=**0.1**;

estimate "Metabolicrate water" TimeBin*Treat*metabolicrate **0** **0** **0** **0** **0** **0** **0** **1** **0** **0** **0** **0**/cl divisor=**1** alpha=**0.1**;

estimate "Timebin3. ";

estimate "Metabolicrate all" TimeBin*Treat*metabolicrate **0** **0** **0** **0** **0** **0** **0** **0** **1** **1** **1** **1**/cl divisor=**4** alpha=**0.1**;

estimate "Metabolicrate hyper" TimeBin*Treat*metabolicrate **0** **0** **0** **0** **0** **0** **0** **0** **1** **0** **0** **0**/cl divisor=**1** alpha=**0.1**;

estimate "Metabolicrate hypo" TimeBin*Treat*metabolicrate **0** **0** **0** **0** **0** **0** **0** **0** **0** **1** **0** **0**/cl divisor=**1** alpha=**0.1**;

estimate "Metabolicrate iso" TimeBin*Treat*metabolicrate **0** **0** **0** **0** **0** **0** **0** **0** **0** **0** **1** **0**/cl divisor=**1** alpha=**0.1**;

estimate "Metabolicrate water" TimeBin*Treat*metabolicrate **0** **0** **0** **0** **0** **0** **0** **0** **0** **0** **0** **1**/cl divisor=**1** alpha=**0.1**;

/*

estimate "heatindex all" TimeBin*Treat*heatindex 1 1 1 1 1 1 1 1 1 1 1 1/cl divisor=12 alpha=0.1;

estimate "heatindex hyper" TimeBin*Treat*heatindex 1 0 0 0 1 0 0 0 1 0 0 0/cl divisor=3 alpha=0.1;

estimate "heatindex hypo" TimeBin*Treat*heatindex 0 1 0 0 0 1 0 0 0 1 0 0/cl divisor=3 alpha=0.1;

estimate "heatindex iso" TimeBin*Treat*heatindex 0 0 1 0 0 0 1 0 0 0 1 0/cl divisor=3 alpha=0.1;

estimate "heatindex water" TimeBin*Treat*heatindex 0 0 0 1 0 0 0 1 0 0 0 1/cl divisor=3 alpha=0.1;

*/

estimate " ";

estimate "Effects of 2SD AvgIngestionRate";

estimate "AvgIngestionRate all" TimeBin*Treat*avg_ingestion_rate **1** **1** **1** **1** **1** **1** **1** **1** **1** **1** **1** **1**/cl divisor=**12** alpha=**0.1**;

estimate "AvgIngestionRate hyper" TimeBin*Treat*avg_ingestion_rate **1** **0** **0** **0** **1** **0** **0** **0** **1** **0** **0** **0**/cl divisor=**3** alpha=**0.1**;

estimate "AvgIngestionRate hypo" TimeBin*Treat*avg_ingestion_rate **0** **1** **0** **0** **0** **1** **0** **0** **0** **1** **0** **0**/cl divisor=**3** alpha=**0.1**;

estimate "AvgIngestionRate iso" TimeBin*Treat*avg_ingestion_rate **0** **0** **1** **0** **0** **0** **1** **0** **0** **0** **1** **0**/cl divisor=**3** alpha=**0.1**;

estimate "AvgIngestionRate water" TimeBin*Treat*avg_ingestion_rate **0** **0** **0** **1** **0** **0** **0** **1** **0** **0** **0** **1**/cl divisor=**3** alpha=**0.1**;

estimate "Timebin1. ";

estimate "AvgIngestionRate all" TimeBin*Treat*avg_ingestion_rate **1** **1** **1** **1** **0** **0** **0** **0** **0** **0** **0** **0**/cl divisor=**4** alpha=**0.1**;

estimate "AvgIngestionRate hyper" TimeBin*Treat*avg_ingestion_rate **1** **0** **0** **0** **0** **0** **0** **0** **0** **0** **0** **0**/cl divisor=**1** alpha=**0.1**;

estimate "AvgIngestionRate hypo" TimeBin*Treat*avg_ingestion_rate **0** **1** **0** **0** **0** **0** **0** **0** **0** **0** **0**/cl divisor=**1** alpha=**0.1**;

estimate "AvgIngestionRate iso" TimeBin*Treat*avg_ingestion_rate **0** **0** **1** **0** **0** **0** **0** **0** **0** **0** **0** **0** **0**/cl divisor=**1** alpha=**0.1**;

estimate "AvgIngestionRate water" TimeBin*Treat*avg_ingestion_rate **0** **0** **0** **1** **0** **0** **0** **0** **0** **0** **0** **0**/cl divisor=**1** alpha=**0.1**;

estimate "Timebin2. ";

estimate "AvgIngestionRate all" TimeBin*Treat*avg_ingestion_rate **0** **0** **0** **0** **1** **1** **1** **1** **0** **0** **0** **0**/cl divisor=**4** alpha=**0.1**;

estimate "AvgIngestionRate hyper" TimeBin*Treat*avg_ingestion_rate **0** **0** **0** **0** **1** **0** **0** **0** **0** **0** **0** **0**/cl divisor=**1** alpha=**0.1**;

estimate "AvgIngestionRate hypo" TimeBin*Treat*avg_ingestion_rate **0** **0** **0** **0** **0** **1** **0** **0** **0** **0** **0** **0**/cl divisor=**1** alpha=**0.1**;

estimate "AvgIngestionRate iso" TimeBin*Treat*avg_ingestion_rate **0** **0** **0** **0** **0** **0** **1** **0** **0** **0** **0** **0**/cl divisor=**1** alpha=**0.1**;

estimate "AvgIngestionRate water" TimeBin*Treat*avg_ingestion_rate **0** **0** **0** **0** **0** **0** **0** **1** **0** **0** **0** **0**/cl divisor=**1** alpha=**0.1**;

estimate "Timebin3. ";

estimate "AvgIngestionRate all" TimeBin*Treat*avg_ingestion_rate **0** **0** **0** **0** **0** **0** **0** **0** **1** **1** **1** **1**/cl divisor=**4** alpha=**0.1**;

estimate "AvgIngestionRate hyper" TimeBin*Treat*avg_ingestion_rate **0** **0** **0** **0** **0** **0** **0** **0** **1** **0** **0** **0**/cl divisor=**1** alpha=**0.1**;

estimate "AvgIngestionRate hypo" TimeBin*Treat*avg_ingestion_rate **0** **0** **0** **0** **0** **0** **0** **0** **0** **1** **0** **0**/cl divisor=**1** alpha=**0.1**;

estimate "AvgIngestionRate iso" TimeBin*Treat*avg_ingestion_rate **0** **0** **0** **0** **0** **0** **0** **0** **0** **0** **1** **0**/cl divisor=**1** alpha=**0.1**;

estimate "AvgIngestionRate water" TimeBin*Treat*avg_ingestion_rate **0** **0** **0** **0** **0** **0** **0** **0** **0** **0** **0** **1**/cl divisor=**1** alpha=**0.1**;

/*

estimate " ";

estimate "Effects of 2SD FrDi";

estimate "FrDi all" TimeBin*Treat*FrDi 1 1 1 1 1 1 1 1 1 1 1 1/cl divisor=12 alpha=0.1;

estimate "FrDi hyper" TimeBin*Treat*FrDi 1 0 0 0 1 0 0 0 1 0 0 0/cl divisor=3 alpha=0.1;

estimate "FrDi hypo" TimeBin*Treat*FrDi 0 1 0 0 0 1 0 0 0 1 0 0/cl divisor=3 alpha=0.1;

estimate "FrDi iso" TimeBin*Treat*FrDi 0 0 1 0 0 0 1 0 0 0 1 0/cl divisor=3 alpha=0.1;

estimate "FrDi water" TimeBin*Treat*FrDi 0 0 0 1 0 0 0 1 0 0 0 1/cl divisor=3 alpha=0.1;

estimate "Timebin1. ";

estimate "FrDi all" TimeBin*Treat*FrDi 1 1 1 1 0 0 0 0 0 0 0 0/cl divisor=4 alpha=0.1;

estimate "FrDi hyper" TimeBin*Treat*FrDi 1 0 0 0 0 0 0 0 0 0 0 0/cl divisor=1 alpha=0.1;

estimate "FrDi hypo" TimeBin*Treat*FrDi 0 1 0 0 0 0 0 0 0 0 0/cl divisor=1 alpha=0.1;

estimate "FrDi iso" TimeBin*Treat*FrDi 0 0 1 0 0 0 0 0 0 0 0 0 0/cl divisor=1 alpha=0.1;

estimate "FrDi water" TimeBin*Treat*FrDi 0 0 0 1 0 0 0 0 0 0 0 0/cl divisor=1 alpha=0.1;

estimate "Timebin2. ";

estimate "FrDi all" TimeBin*Treat*FrDi 0 0 0 0 1 1 1 1 0 0 0 0/cl divisor=4 alpha=0.1;

estimate "FrDi hyper" TimeBin*Treat*FrDi 0 0 0 0 1 0 0 0 0 0 0 0/cl divisor=1 alpha=0.1;

estimate "FrDi hypo" TimeBin*Treat*FrDi 0 0 0 0 0 1 0 0 0 0 0 0/cl divisor=1 alpha=0.1;

estimate "FrDi iso" TimeBin*Treat*FrDi 0 0 0 0 0 0 1 0 0 0 0 0/cl divisor=1 alpha=0.1;

estimate "FrDi water" TimeBin*Treat*FrDi 0 0 0 0 0 0 0 1 0 0 0 0/cl divisor=1 alpha=0.1;

estimate "Timebin3. ";

estimate "FrDi all" TimeBin*Treat*FrDi 0 0 0 0 0 0 0 0 1 1 1 1/cl divisor=4 alpha=0.1;

estimate "FrDi hyper" TimeBin*Treat*FrDi 0 0 0 0 0 0 0 0 1 0 0 0/cl divisor=1 alpha=0.1;

estimate "FrDi hypo" TimeBin*Treat*FrDi 0 0 0 0 0 0 0 0 0 1 0 0/cl divisor=1 alpha=0.1;

estimate "FrDi iso" TimeBin*Treat*FrDi 0 0 0 0 0 0 0 0 0 0 1 0/cl divisor=1 alpha=0.1;

estimate "FrDi water" TimeBin*Treat*FrDi 0 0 0 0 0 0 0 0 0 0 0 1/cl divisor=1 alpha=0.1;

estimate " ";

estimate "Effects of 2SD FrPoly";

estimate "FrPoly all" TimeBin*Treat*FrPoly 1 1 1 1 1 1 1 1 1 1 1 1/cl divisor=12 alpha=0.1;

estimate "FrPoly hyper" TimeBin*Treat*FrPoly 1 0 0 0 1 0 0 0 1 0 0 0/cl divisor=3 alpha=0.1;

estimate "FrPoly hypo" TimeBin*Treat*FrPoly 0 1 0 0 0 1 0 0 0 1 0 0/cl divisor=3 alpha=0.1;

estimate "FrPoly iso" TimeBin*Treat*FrPoly 0 0 1 0 0 0 1 0 0 0 1 0/cl divisor=3 alpha=0.1;

estimate "FrPoly water" TimeBin*Treat*FrPoly 0 0 0 1 0 0 0 1 0 0 0 1/cl divisor=3 alpha=0.1;

estimate "Timebin1. ";

estimate "FrPoly all" TimeBin*Treat*FrPoly 1 1 1 1 0 0 0 0 0 0 0 0/cl divisor=4 alpha=0.1;

estimate "FrPoly hyper" TimeBin*Treat*FrPoly 1 0 0 0 0 0 0 0 0 0 0 0/cl divisor=1 alpha=0.1;

estimate "FrPoly hypo" TimeBin*Treat*FrPoly 0 1 0 0 0 0 0 0 0 0 0/cl divisor=1 alpha=0.1;

estimate "FrPoly iso" TimeBin*Treat*FrPoly 0 0 1 0 0 0 0 0 0 0 0 0 0/cl divisor=1 alpha=0.1;

estimate "FrPoly water" TimeBin*Treat*FrPoly 0 0 0 1 0 0 0 0 0 0 0 0/cl divisor=1 alpha=0.1;

estimate "Timebin2. ";

estimate "FrPoly all" TimeBin*Treat*FrPoly 0 0 0 0 1 1 1 1 0 0 0 0/cl divisor=4 alpha=0.1;

estimate "FrPoly hyper" TimeBin*Treat*FrPoly 0 0 0 0 1 0 0 0 0 0 0 0/cl divisor=1 alpha=0.1;

estimate "FrPoly hypo" TimeBin*Treat*FrPoly 0 0 0 0 0 1 0 0 0 0 0 0/cl divisor=1 alpha=0.1;

estimate "FrPoly iso" TimeBin*Treat*FrPoly 0 0 0 0 0 0 1 0 0 0 0 0/cl divisor=1 alpha=0.1;

estimate "FrPoly water" TimeBin*Treat*FrPoly 0 0 0 0 0 0 0 1 0 0 0 0/cl divisor=1 alpha=0.1;

estimate "Timebin3. ";

estimate "FrPoly all" TimeBin*Treat*FrPoly 0 0 0 0 0 0 0 0 1 1 1 1/cl divisor=4 alpha=0.1;

estimate "FrPoly hyper" TimeBin*Treat*FrPoly 0 0 0 0 0 0 0 0 1 0 0 0/cl divisor=1 alpha=0.1;

estimate "FrPoly hypo" TimeBin*Treat*FrPoly 0 0 0 0 0 0 0 0 0 1 0 0/cl divisor=1 alpha=0.1;

estimate "FrPoly iso" TimeBin*Treat*FrPoly 0 0 0 0 0 0 0 0 0 0 1 0/cl divisor=1 alpha=0.1;

estimate "FrPoly water" TimeBin*Treat*FrPoly 0 0 0 0 0 0 0 0 0 0 0 1/cl divisor=1 alpha=0.1;

*/

/*

estimate " ";

estimate "Effects of 2SD CHOConc_Tot__gPC";

estimate "CHOConc_Tot__gPC all" TimeBin*Treat*CHOConc_Tot__gPC 1 1 1 1 1 1 1 1 1 1 1 1/cl divisor=12 alpha=0.1;

estimate "CHOConc_Tot__gPC hyper" TimeBin*Treat*CHOConc_Tot__gPC 1 0 0 0 1 0 0 0 1 0 0 0/cl divisor=3 alpha=0.1;

estimate "CHOConc_Tot__gPC hypo" TimeBin*Treat*CHOConc_Tot__gPC 0 1 0 0 0 1 0 0 0 1 0 0/cl divisor=3 alpha=0.1;

estimate "CHOConc_Tot__gPC iso" TimeBin*Treat*CHOConc_Tot__gPC 0 0 1 0 0 0 1 0 0 0 1 0/cl divisor=3 alpha=0.1;

estimate "CHOConc_Tot__gPC water" TimeBin*Treat*CHOConc_Tot__gPC 0 0 0 1 0 0 0 1 0 0 0 1/cl divisor=3 alpha=0.1;

estimate "Timebin1. ";

estimate "CHOConc_Tot__gPC all" TimeBin*Treat*CHOConc_Tot__gPC 1 1 1 1 0 0 0 0 0 0 0 0/cl divisor=4 alpha=0.1;

estimate "CHOConc_Tot__gPC hyper" TimeBin*Treat*CHOConc_Tot__gPC 1 0 0 0 0 0 0 0 0 0 0 0/cl divisor=1 alpha=0.1;

estimate "CHOConc_Tot__gPC hypo" TimeBin*Treat*CHOConc_Tot__gPC 0 1 0 0 0 0 0 0 0 0 0/cl divisor=1 alpha=0.1;

estimate "CHOConc_Tot__gPC iso" TimeBin*Treat*CHOConc_Tot__gPC 0 0 1 0 0 0 0 0 0 0 0 0 0/cl divisor=1 alpha=0.1;

estimate "CHOConc_Tot__gPC water" TimeBin*Treat*CHOConc_Tot__gPC 0 0 0 1 0 0 0 0 0 0 0 0/cl divisor=1 alpha=0.1;

estimate "Timebin2. ";

estimate "CHOConc_Tot__gPC all" TimeBin*Treat*CHOConc_Tot__gPC 0 0 0 0 1 1 1 1 0 0 0 0/cl divisor=4 alpha=0.1;

estimate "CHOConc_Tot__gPC hyper" TimeBin*Treat*CHOConc_Tot__gPC 0 0 0 0 1 0 0 0 0 0 0 0/cl divisor=1 alpha=0.1;

estimate "CHOConc_Tot__gPC hypo" TimeBin*Treat*CHOConc_Tot__gPC 0 0 0 0 0 1 0 0 0 0 0 0/cl divisor=1 alpha=0.1;

estimate "CHOConc_Tot__gPC iso" TimeBin*Treat*CHOConc_Tot__gPC 0 0 0 0 0 0 1 0 0 0 0 0/cl divisor=1 alpha=0.1;

estimate "CHOConc_Tot__gPC water" TimeBin*Treat*CHOConc_Tot__gPC 0 0 0 0 0 0 0 1 0 0 0 0/cl divisor=1 alpha=0.1;

estimate "Timebin3. ";

estimate "CHOConc_Tot__gPC all" TimeBin*Treat*CHOConc_Tot__gPC 0 0 0 0 0 0 0 0 1 1 1 1/cl divisor=4 alpha=0.1;

estimate "CHOConc_Tot__gPC hyper" TimeBin*Treat*CHOConc_Tot__gPC 0 0 0 0 0 0 0 0 1 0 0 0/cl divisor=1 alpha=0.1;

estimate "CHOConc_Tot__gPC hypo" TimeBin*Treat*CHOConc_Tot__gPC 0 0 0 0 0 0 0 0 0 1 0 0/cl divisor=1 alpha=0.1;

estimate "CHOConc_Tot__gPC iso" TimeBin*Treat*CHOConc_Tot__gPC 0 0 0 0 0 0 0 0 0 0 1 0/cl divisor=1 alpha=0.1;

estimate "CHOConc_Tot__gPC water" TimeBin*Treat*CHOConc_Tot__gPC 0 0 0 0 0 0 0 0 0 0 0 1/cl divisor=1 alpha=0.1;

estimate " ";

estimate "Effects of 2SD Effective_Frucratio";

estimate "Effective_Frucratio all" TimeBin*Treat*Effective_Frucratio 1 1 1 1 1 1 1 1 1 1 1 1/cl divisor=12 alpha=0.1;

estimate "Effective_Frucratio hyper" TimeBin*Treat*Effective_Frucratio 1 0 0 0 1 0 0 0 1 0 0 0/cl divisor=3 alpha=0.1;

estimate "Effective_Frucratio hypo" TimeBin*Treat*Effective_Frucratio 0 1 0 0 0 1 0 0 0 1 0 0/cl divisor=3 alpha=0.1;

estimate "Effective_Frucratio iso" TimeBin*Treat*Effective_Frucratio 0 0 1 0 0 0 1 0 0 0 1 0/cl divisor=3 alpha=0.1;

estimate "Effective_Frucratio water" TimeBin*Treat*Effective_Frucratio 0 0 0 1 0 0 0 1 0 0 0 1/cl divisor=3 alpha=0.1;

estimate "Timebin1. ";

estimate "Effective_Frucratio all" TimeBin*Treat*Effective_Frucratio 1 1 1 1 0 0 0 0 0 0 0 0/cl divisor=4 alpha=0.1;

estimate "Effective_Frucratio hyper" TimeBin*Treat*Effective_Frucratio 1 0 0 0 0 0 0 0 0 0 0 0/cl divisor=1 alpha=0.1;

estimate "Effective_Frucratio hypo" TimeBin*Treat*Effective_Frucratio 0 1 0 0 0 0 0 0 0 0 0/cl divisor=1 alpha=0.1;

estimate "Effective_Frucratio iso" TimeBin*Treat*Effective_Frucratio 0 0 1 0 0 0 0 0 0 0 0 0 0/cl divisor=1 alpha=0.1;

estimate "Effective_Frucratio water" TimeBin*Treat*Effective_Frucratio 0 0 0 1 0 0 0 0 0 0 0 0/cl divisor=1 alpha=0.1;

estimate "Timebin2. ";

estimate "Effective_Frucratio all" TimeBin*Treat*Effective_Frucratio 0 0 0 0 1 1 1 1 0 0 0 0/cl divisor=4 alpha=0.1;

estimate "Effective_Frucratio hyper" TimeBin*Treat*Effective_Frucratio 0 0 0 0 1 0 0 0 0 0 0 0/cl divisor=1 alpha=0.1;

estimate "Effective_Frucratio hypo" TimeBin*Treat*Effective_Frucratio 0 0 0 0 0 1 0 0 0 0 0 0/cl divisor=1 alpha=0.1;

estimate "Effective_Frucratio iso" TimeBin*Treat*Effective_Frucratio 0 0 0 0 0 0 1 0 0 0 0 0/cl divisor=1 alpha=0.1;

estimate "Effective_Frucratio water" TimeBin*Treat*Effective_Frucratio 0 0 0 0 0 0 0 1 0 0 0 0/cl divisor=1 alpha=0.1;

estimate "Timebin3. ";

estimate "Effective_Frucratio all" TimeBin*Treat*Effective_Frucratio 0 0 0 0 0 0 0 0 1 1 1 1/cl divisor=4 alpha=0.1;

estimate "Effective_Frucratio hyper" TimeBin*Treat*Effective_Frucratio 0 0 0 0 0 0 0 0 1 0 0 0/cl divisor=1 alpha=0.1;

estimate "Effective_Frucratio hypo" TimeBin*Treat*Effective_Frucratio 0 0 0 0 0 0 0 0 0 1 0 0/cl divisor=1 alpha=0.1;

estimate "Effective_Frucratio iso" TimeBin*Treat*Effective_Frucratio 0 0 0 0 0 0 0 0 0 0 1 0/cl divisor=1 alpha=0.1;

estimate "Effective_Frucratio water" TimeBin*Treat*Effective_Frucratio 0 0 0 0 0 0 0 0 0 0 0 1/cl divisor=1 alpha=0.1;

*/

****;

estimate " ";

estimate "DrinkOsm Initial Analysis V.likely d Analysis";

estimate "DrinkOsm V.likely d Analysis";

estimate "TimeBinMeansOverall mean setting";*automatically adjusted for standardised covariates. If not standardised then need to put in the covariate;

estimate "Hyper" TimeBin*Treat **1** **0** **0** **0** **1** **0** **0** **0** **1** **0** **0** **0** TimeBin*Treat*DrinkOsMAdj **98.6** **0** **0** **0** **98.6** **0** **0** **0** **98.6** **0** **0** **0**/cl divisor=**3** alpha=**0.1**;

estimate "Hypo" TimeBin*Treat **0** **1** **0** **0** **0** **1** **0** **0** **0** **1** **0** **0** TimeBin*Treat*DrinkOsMAdj **0** -**108.2** **0** **0** **0** -**108.2** **0** **0** **0** -**108.2** **0** **0**/cl divisor=**3** alpha=**0.1**;

estimate "Iso" TimeBin*Treat **0** **0** **1** **0** **0** **0** **1** **0** **0** **0** **1** **0** TimeBin*Treat*DrinkOsMAdj **0** **0** -**3.2** **0** **0** **0** -**3.2** **0** **0** **0** -**3.2** **0**/cl divisor=**3** alpha=**0.1**;

estimate "Water" TimeBin*Treat **0** **0** **0** **1** **0** **0** **0** **1** **0** **0** **0** **1** /cl divisor=**3** alpha=**0.1**;

estimate "Hyper-iso" TimeBin*Treat **1** **0** -**1** **0** **1** **0** -**1** **0** **1** **0** -**1** **0** TimeBin*Treat*DrinkOsMAdj **98.6** **0** **3.2** **0** **98.6** **0** **3.2** **0** **98.6** **0** **3.2** **0**/cl divisor=**3** alpha=**0.1**;

estimate "Hypo-iso" TimeBin*Treat **0** **1** -**1** **0** **0** **1** -**1** **0** **0** **1** -**1** **0** TimeBin*Treat*DrinkOsMAdj **0** -**108.2** **3.2** **0** **0** -**108.2** **3.2** **0** **0** -**108.2** **3.2** **0**/cl divisor=**3** alpha=**0.1**;

estimate "Water-iso" TimeBin*Treat **0** **0** -**1** **1** **0** **0** -**1** **1** **0** **0** -**1** **1** TimeBin*Treat*DrinkOsMAdj **0** **0** **3.2** **0** **0** **0** **3.2** **0** **0** **0** **3.2** **0**/cl divisor=**3** alpha=**0.1**;

estimate "Hyper-Hypo" TimeBin*Treat **1** -**1** **0** **0** **1** -**1** **0** **0** **1** -**1** **0** **0** TimeBin*Treat*DrinkOsMAdj **98.6** **108.2** **0** **0** **98.6** **108.2** **0** **0** **98.6** **103.1** **0** **0**/cl divisor=**3** alpha=**0.1**; *check iso is a postivie then ave covar is a -103.1;

estimate "Hyper-Water" TimeBin*Treat **1** **0** **0** -**1** **1** **0** **0** -**1** **1** **0** **0** -**1** TimeBin*Treat*DrinkOsMAdj **98.6** **0** **0** **0** **98.6** **0** **0** **0** **98.6** **0** **0** **0**/cl divisor=**3** alpha=**0.1**;

estimate "Hypo-Water" TimeBin*Treat **0** **1** **0** -**1** **0** **1** **0** -**1** **0** **1** **0** -**1** TimeBin*Treat*DrinkOsMAdj **0** -**108.2** **0** **0** **0** -**108.2** **0** **0** **0** -**108.2** **0** **0**/cl divisor=**3** alpha=**0.1**;

estimate "TimeBinMeansOverall indiv setting";*automatically adjusted for standardised covariates. If not standardised then need to put in the covariate;

estimate "Hyper" TimeBin*Treat **1** **0** **0** **0** **1** **0** **0** **0** **1** **0** **0** **0** TimeBin*Treat*DrinkOsMAdj **98.6** **0** **0** **0** **98.6** **0** **0** **0** **98.6** **0** **0** **0**|StudyID **1** StudyID*ExptID **1** StudyID*EstimateID **1**/cl divisor=**3** alpha=**0.1**;

estimate "Hypo" TimeBin*Treat **0** **1** **0** **0** **0** **1** **0** **0** **0** **1** **0** **0** TimeBin*Treat*DrinkOsMAdj **0** -**108.2** **0** **0** **0** -**108.2** **0** **0** **0** -**108.2** **0** **0**|StudyID **1** StudyID*ExptID **1** StudyID*EstimateID **1**/cl divisor=**3** alpha=**0.1**;

estimate "Iso" TimeBin*Treat **0** **0** **1** **0** **0** **0** **1** **0** **0** **0** **1** **0** TimeBin*Treat*DrinkOsMAdj **0** **0** -**3.2** **0** **0** **0** -**3.2** **0** **0** **0** -**3.2** **0**|StudyID **1** StudyID*ExptID **1** StudyID*EstimateID **1**/cl divisor=**3** alpha=**0.1**;

estimate "Water" TimeBin*Treat **0** **0** **0** **1** **0** **0** **0** **1** **0** **0** **0** **1** |StudyID **1** StudyID*ExptID **1** StudyID*EstimateID **1**/cl divisor=**3** alpha=**0.1**;

estimate "Hyper-iso" TimeBin*Treat **1** **0** -**1** **0** **1** **0** -**1** **0** **1** **0** -**1** **0** TimeBin*Treat*DrinkOsMAdj **98.6** **0** **3.2** **0** **98.6** **0** **3.2** **0** **98.6** **0** **3.2** **0**|StudyID **1** StudyID*ExptID **1** StudyID*EstimateID **1**/cl divisor=**3** alpha=**0.1**;

estimate "Hypo-iso" TimeBin*Treat **0** **1** -**1** **0** **0** **1** -**1** **0** **0** **1** -**1** **0** TimeBin*Treat*DrinkOsMAdj **0** -**108.2** **3.2** **0** **0** -**108.2** **3.2** **0** **0** -**108.2** **3.2** **0**|StudyID **1** StudyID*ExptID **1** StudyID*EstimateID **1**/cl divisor=**3** alpha=**0.1**;

estimate "Water-iso" TimeBin*Treat **0** **0** -**1** **1** **0** **0** -**1** **1** **0** **0** -**1** **1** TimeBin*Treat*DrinkOsMAdj **0** **0** **3.2** **0** **0** **0** **3.2** **0** **0** **0** **3.2** **0**|StudyID **1** StudyID*ExptID **1** StudyID*EstimateID **1**/cl divisor=**3** alpha=**0.1**;

estimate "Hyper-Hypo" TimeBin*Treat **1** -**1** **0** **0** **1** -**1** **0** **0** **1** -**1** **0** **0** TimeBin*Treat*DrinkOsMAdj **98.6** **108.2** **0** **0** **98.6** **108.2** **0** **0** **98.6** **103.1** **0** **0**|StudyID **1** StudyID*ExptID **1** StudyID*EstimateID **1**/cl divisor=**3** alpha=**0.1**; *check iso is a postivie then ave covar is a -103.1;

estimate "Hyper-Water" TimeBin*Treat **1** **0** **0** -**1** **1** **0** **0** -**1** **1** **0** **0** -**1** TimeBin*Treat*DrinkOsMAdj **98.6** **0** **0** **0** **98.6** **0** **0** **0** **98.6** **0** **0** **0**|StudyID **1** StudyID*ExptID **1** StudyID*EstimateID **1**/cl divisor=**3** alpha=**0.1**;

estimate "Hypo-Water" TimeBin*Treat **0** **1** **0** -**1** **0** **1** **0** -**1** **0** **1** **0** -**1** TimeBin*Treat*DrinkOsMAdj **0** -**108.2** **0** **0** **0** -**108.2** **0** **0** **0** -**108.2** **0** **0**|StudyID **1** StudyID*ExptID **1** StudyID*EstimateID **1**/cl divisor=**3** alpha=**0.1**;

estimate " ";

estimate "DrinkOsMAdj V.likely d Analysis";

estimate "TimeBin1.<31 mean setting";

estimate "Hyper" TimeBin*Treat **1** **0** **0** **0** **0** **0** **0** **0** **0** **0** **0** **0** TimeBin*Treat*DrinkOsMAdj **98.6** **0** **0** **0** **0** **0** **0** **0** **0** **0** **0** **0**/cl divisor=**1** alpha=**0.1**;

estimate "Hypo" TimeBin*Treat **0** **1** **0** **0** **0** **0** **0** **0** **0** **0** **0** **0** TimeBin*Treat*DrinkOsMAdj **0** -**108.2** **0** **0** **0** **0** **0** **0** **0** **0** **0** **0**/cl divisor=**1** alpha=**0.1**;

estimate "Iso" TimeBin*Treat **0** **0** **1** **0** **0** **0** **0** **0** **0** **0** **0** **0** TimeBin*Treat*DrinkOsMAdj **0** **0** -**3.2** **0** **0** **0** **0** **0** **0** **0** **0** **0**/cl divisor=**1** alpha=**0.1**;

estimate "Water" TimeBin*Treat **0** **0** **0** **1** **0** **0** **0** **0** **0** **0** **0** **0** /cl divisor=**1** alpha=**0.1**;

estimate "Hyper-iso" TimeBin*Treat **1** **0** -**1** **0** **0** **0** **0** **0** **0** **0** **0** **0** TimeBin*Treat*DrinkOsMAdj **98.6** **0** **3.2** **0** **0** **0** **0** **0** **0** **0** **0** **0**/cl divisor=**1** alpha=**0.1**;

estimate "Hypo-iso" TimeBin*Treat **0** **1** -**1** **0** **0** **0** **0** **0** **0** **0** **0** **0** TimeBin*Treat*DrinkOsMAdj **0** -**108.2** **3.2** **0** **0** **0** **0** **0** **0** **0** **0** **0**/cl divisor=**1** alpha=**0.1**;

estimate "Water-iso" TimeBin*Treat **0** **0** -**1** **1** **0** **0** **0** **0** **0** **0** **0** **0** TimeBin*Treat*DrinkOsMAdj **0** **0** **3.2** **0** **0** **0** **0** **0** **0** **0** **0** **0**/cl divisor=**1** alpha=**0.1**;

estimate "Hyper-Hypo" TimeBin*Treat **1** -**1** **0** **0** **0** **0** **0** **0** **0** **0** **0** **0** TimeBin*Treat*DrinkOsMAdj **98.6** **108.2** **0** **0** **0** **0** **0** **0** **0** **0** **0** **0**/cl divisor=**1** alpha=**0.1**; *check iso is a postivie then ave covar is a -103.1;

estimate "Hyper-Water" TimeBin*Treat **1** **0** **0** -**1** **0** **0** **0** **0** **0** **0** **0** **0** TimeBin*Treat*DrinkOsMAdj **98.6** **0** **0** **0** **0** **0** **0** **0** **0** **0** **0** **0**/cl divisor=**1** alpha=**0.1**;

estimate "Hypo-Water" TimeBin*Treat **0** **1** **0** -**1** **0** **0** **0** **0** **0** **0** **0** **0** TimeBin*Treat*DrinkOsMAdj **0** -**108.2** **0** **0** **0** **0** **0** **0** **0** **0** **0** **0**/cl divisor=**1** alpha=**0.1**;

estimate "TimeBin2.31-60 mean setting";

estimate "Hyper" TimeBin*Treat **0** **0** **0** **0** **1** **0** **0** **0** **0** **0** **0** **0** TimeBin*Treat*DrinkOsMAdj **0** **0** **0** **0** **98.6** **0** **0** **0** **0** **0** **0** **0**/cl divisor=**1** alpha=**0.1**;

estimate "Hypo" TimeBin*Treat **0** **0** **0** **0** **0** **1** **0** **0** **0** **0** **0** **0** TimeBin*Treat*DrinkOsMAdj **0** **0** **0** **0** **0** -**108.2** **0** **0** **0** **0** **0** **0**/cl divisor=**1** alpha=**0.1**;

estimate "Iso" TimeBin*Treat **0** **0** **0** **0** **0** **0** **1** **0** **0** **0** **0** **0** TimeBin*Treat*DrinkOsMAdj **0** **0** **0** **0** **0** **0** -**3.2** **0** **0** **0** **0** **0**/cl divisor=**1** alpha=**0.1**;

estimate "Water" TimeBin*Treat **0** **0** **0** **0** **0** **0** **0** **1** **0** **0** **0** **0** /cl divisor=**1** alpha=**0.1**;

estimate "Hyper-iso" TimeBin*Treat **0** **0** **0** **0** **1** **0** -**1** **0** **0** **0** **0** **0** TimeBin*Treat*DrinkOsMAdj **0** **0** **0** **0** **98.6** **0** **3.2** **0** **0** **0** **0** **0**/cl divisor=**1** alpha=**0.1**;

estimate "Hypo-iso" TimeBin*Treat **0** **0** **0** **0** **0** **1** -**1** **0** **0** **0** **0** **0** TimeBin*Treat*DrinkOsMAdj **0** **0** **0** **0** **0** -**108.2** **3.2** **0** **0** **0** **0** **0**/cl divisor=**1** alpha=**0.1**;

estimate "Water-iso" TimeBin*Treat **0** **0** **0** **0** **0** **0** -**1** **1** **0** **0** **0** **0** TimeBin*Treat*DrinkOsMAdj **0** **0** **0** **0** **0** **0** **3.2** **0** **0** **0** **0** **0**/cl divisor=**1** alpha=**0.1**;

estimate "Hyper-Hypo" TimeBin*Treat **0** **0** **0** **0** **1** -**1** **0** **0** **0** **0** **0** **0** TimeBin*Treat*DrinkOsMAdj **0** **0** **0** **0** **98.6** **108.2** **0** **0** **0** **0** **0** **0**/cl divisor=**1** alpha=**0.1**; *check iso is a postivie then ave covar is a -103.1;

estimate "Hyper-Water" TimeBin*Treat **0** **0** **0** **0** **1** **0** **0** -**1** **0** **0** **0** **0** TimeBin*Treat*DrinkOsMAdj **0** **0** **0** **0** **98.6** **0** **0** **0** **0** **0** **0** **0**/cl divisor=**1** alpha=**0.1**;

estimate "Hypo-Water" TimeBin*Treat **0** **0** **0** **0** **0** **1** **0** -**1** **0** **0** **0** **0** TimeBin*Treat*DrinkOsMAdj **0** **0** **0** **0** **0** -**108.2** **0** **0** **0** **0** **0** **0**/cl divisor=**1** alpha=**0.1**;

estimate "TimeBin3.60-180 mean setting";

estimate "Hyper" TimeBin*Treat **0** **0** **0** **0** **0** **0** **0** **0** **1** **0** **0** **0** TimeBin*Treat*DrinkOsMAdj **0** **0** **0** **0** **0** **0** **0** **0** **98.6** **0** **0** **0**/cl divisor=**1** alpha=**0.1**;

estimate "Hypo" TimeBin*Treat **0** **0** **0** **0** **0** **0** **0** **0** **0** **1** **0** **0** TimeBin*Treat*DrinkOsMAdj **0** **0** **0** **0** **0** **0** **0** **0** **0** -**108.2** **0** **0**/cl divisor=**1** alpha=**0.1**;

estimate "Iso" TimeBin*Treat **0** **0** **0** **0** **0** **0** **0** **0** **0** **0** **1** **0** TimeBin*Treat*DrinkOsMAdj **0** **0** **0** **0** **0** **0** **0** **0** **0** **0** -**3.2** **0**/cl divisor=**1** alpha=**0.1**;

estimate "Water" TimeBin*Treat **0** **0** **0** **0** **0** **0** **0** **0** **0** **0** **0** **1** /cl divisor=**1** alpha=**0.1**;

estimate "Hyper-iso" TimeBin*Treat **0** **0** **0** **0** **0** **0** **0** **0** **1** **0** -**1** **0** TimeBin*Treat*DrinkOsMAdj **0** **0** **0** **0** **0** **0** **0** **0** **98.6** **0** **3.2** **0**/cl divisor=**1** alpha=**0.1**;

estimate "Hypo-iso" TimeBin*Treat **0** **0** **0** **0** **0** **0** **0** **0** **0** **1** -**1** **0** TimeBin*Treat*DrinkOsMAdj **0** **0** **0** **0** **0** **0** **0** **0** **0** -**108.2** **3.2** **0**/cl divisor=**1** alpha=**0.1**;

estimate "Water-iso" TimeBin*Treat **0** **0** **0** **0** **0** **0** **0** **0** **0** **0** -**1** **1** TimeBin*Treat*DrinkOsMAdj **0** **0** **0** **0** **0** **0** **0** **0** **0** **0** **3.2** **0**/cl divisor=**1** alpha=**0.1**;

estimate "Hyper-Hypo" TimeBin*Treat **0** **0** **0** **0** **0** **0** **0** **0** **1** -**1** **0** **0** TimeBin*Treat*DrinkOsMAdj **0** **0** **0** **0** **0** **0** **0** **0** **98.6** **103.1** **0** **0**/cl divisor=**1** alpha=**0.1**; *check iso is a postivie then ave covar is a -103.1;

estimate "Hyper-Water" TimeBin*Treat **0** **0** **0** **0** **0** **0** **0** **0** **1** **0** **0** -**1** TimeBin*Treat*DrinkOsMAdj **0** **0** **0** **0** **0** **0** **0** **0** **98.6** **0** **0** **0**/cl divisor=**1** alpha=**0.1**;

estimate "Hypo-Water" TimeBin*Treat **0** **0** **0** **0** **0** **0** **0** **0** **0** **1** **0** -**1** TimeBin*Treat*DrinkOsMAdj **0** **0** **0** **0** **0** **0** **0** **0** **0** -**108.2** **0** **0**/cl divisor=**1** alpha=**0.1**;

/*

estimate " ";

estimate "DrinkOsm V.likely d Analysis";

estimate "TimeBinMeansOverall mean setting";*automatically adjusted for standardised covariates. If not standardised then need to put in the covariate;

estimate "Hyper" TimeBin*Treat 1 0 0 0 1 0 0 0 1 0 0 0 TimeBin*Treat*DrinkOsM 95.8 0 0 0 95.8 0 0 0 95.8 0 0 0 TimeBin*Treat*FrDi 1 0 0 0 1 0 0 0 1 0 0 0 TimeBin*Treat*FrPoly 1 0 0 0 1 0 0 0 1 0 0 0/cl divisor=3 alpha=0.1;

estimate "Hypo" TimeBin*Treat 0 1 0 0 0 1 0 0 0 1 0 0 TimeBin*Treat*DrinkOsM 0 -109.1 0 0 0 -109.1 0 0 0 -109.1 0 0 TimeBin*Treat*FrDi 0 1 0 0 0 1 0 0 0 1 0 0 TimeBin*Treat*FrPoly 0 1 0 0 0 1 0 0 0 1 0 0/cl divisor=3 alpha=0.1;

estimate "Iso" TimeBin*Treat 0 0 1 0 0 0 1 0 0 0 1 0 TimeBin*Treat*DrinkOsM 0 0 -2.0 0 0 0 -2.0 0 0 0 -2.0 0 TimeBin*Treat*FrDi 0 0 1 0 0 0 1 0 0 0 1 0 TimeBin*Treat*FrPoly 0 0 1 0 0 0 1 0 0 0 1 0/cl divisor=3 alpha=0.1;

estimate "Water" TimeBin*Treat 0 0 0 1 0 0 0 1 0 0 0 1 TimeBin*Treat*FrDi 0 0 0 1 0 0 0 1 0 0 0 1 TimeBin*Treat*FrPoly 0 0 0 1 0 0 0 1 0 0 0 1/cl divisor=3 alpha=0.1;

estimate "Hyper-iso" TimeBin*Treat 1 0 -1 0 1 0 -1 0 1 0 -1 0 TimeBin*Treat*DrinkOsM 95.8 0 2.0 0 95.8 0 2.0 0 95.8 0 2.0 0 TimeBin*Treat*FrDi 1 0 -1 0 1 0 -1 0 1 0 -1 0 TimeBin*Treat*FrPoly 1 0 -1 0 1 0 -1 0 1 0 -1 0/cl divisor=3 alpha=0.1;

estimate "Hypo-iso" TimeBin*Treat 0 1 -1 0 0 1 -1 0 0 1 -1 0 TimeBin*Treat*DrinkOsM 0 -109.1 2.0 0 0 -109.1 2.0 0 0 -109.1 2.0 0 TimeBin*Treat*FrDi 0 1 -1 0 0 1 -1 0 0 1 -1 0 TimeBin*Treat*FrPoly 0 1 -1 0 0 1 -1 0 0 1 -1 0/cl divisor=3 alpha=0.1;

estimate "Water-iso" TimeBin*Treat 0 0 -1 1 0 0 -1 1 0 0 -1 1 TimeBin*Treat*DrinkOsM 0 0 2.0 0 0 0 2.0 0 0 0 2.0 0 TimeBin*Treat*FrDi 0 0 -1 1 0 0 -1 1 0 0 -1 1 TimeBin*Treat*FrPoly 0 0 -1 1 0 0 -1 1 0 0 -1 1/cl divisor=3 alpha=0.1;

estimate "Hyper-Hypo" TimeBin*Treat 1 -1 0 0 1 -1 0 0 1 -1 0 0 TimeBin*Treat*DrinkOsM 95.8 109.1 0 0 95.8 109.1 0 0 95.8 103.1 0 0 TimeBin*Treat*FrDi 1 -1 0 0 1 -1 0 0 1 -1 0 0TimeBin*Treat*FrPoly 1 -1 0 0 1 -1 0 0 1 -1 0 0/cl divisor=3 alpha=0.1; *check iso is a postivie then ave covar is a -103.1;

estimate "Hyper-Water" TimeBin*Treat 1 0 0 -1 1 0 0 -1 1 0 0 -1 TimeBin*Treat*DrinkOsM 95.8 0 0 0 95.8 0 0 0 95.8 0 0 0 TimeBin*Treat*FrDi 1 0 0 -1 1 0 0 -1 1 0 0 -1 TimeBin*Treat*FrPoly 1 0 0 -1 1 0 0 -1 1 0 0 -1/cl divisor=3 alpha=0.1;

estimate "Hypo-Water" TimeBin*Treat 0 1 0 -1 0 1 0 -1 0 1 0 -1 TimeBin*Treat*DrinkOsM 0 -109.1 0 0 0 -109.1 0 0 0 -109.1 0 0 TimeBin*Treat*FrDi 0 1 0 -1 0 1 0 -1 0 1 0 -1 TimeBin*Treat*FrPoly 0 1 0 -1 0 1 0 -1 0 1 0 -1/cl divisor=3 alpha=0.1;

estimate "TimeBinMeansOverall indiv setting";*automatically adjusted for standardised covariates. If not standardised then need to put in the covariate;

estimate "Hyper" TimeBin*Treat 1 0 0 0 1 0 0 0 1 0 0 0 TimeBin*Treat*DrinkOsM 95.8 0 0 0 95.8 0 0 0 95.8 0 0 0 TimeBin*Treat*FrDi 1 0 0 0 1 0 0 0 1 0 0 0 TimeBin*Treat*FrPoly 1 0 0 0 1 0 0 0 1 0 0 0|StudyID 1 StudyID*ExptID 1 StudyID*EstimateID 1/cl divisor=3 alpha=0.1;

estimate "Hypo" TimeBin*Treat 0 1 0 0 0 1 0 0 0 1 0 0 TimeBin*Treat*DrinkOsM 0 -109.1 0 0 0 -109.1 0 0 0 -109.1 0 0 TimeBin*Treat*FrDi 0 1 0 0 0 1 0 0 0 1 0 0 TimeBin*Treat*FrPoly 0 1 0 0 0 1 0 0 0 1 0 0|StudyID 1 StudyID*ExptID 1 StudyID*EstimateID 1/cl divisor=3 alpha=0.1;

estimate "Iso" TimeBin*Treat 0 0 1 0 0 0 1 0 0 0 1 0 TimeBin*Treat*DrinkOsM 0 0 -2.0 0 0 0 -2.0 0 0 0 -2.0 0 TimeBin*Treat*FrDi 0 0 1 0 0 0 1 0 0 0 1 0 TimeBin*Treat*FrPoly 0 0 1 0 0 0 1 0 0 0 1 0|StudyID 1 StudyID*ExptID 1 StudyID*EstimateID 1/cl divisor=3 alpha=0.1;

estimate "Water" TimeBin*Treat 0 0 0 1 0 0 0 1 0 0 0 1 TimeBin*Treat*FrDi 0 0 0 1 0 0 0 1 0 0 0 1 TimeBin*Treat*FrPoly 0 0 0 1 0 0 0 1 0 0 0 1|StudyID 1 StudyID*ExptID 1 StudyID*EstimateID 1/cl divisor=3 alpha=0.1;

estimate "Hyper-iso" TimeBin*Treat 1 0 -1 0 1 0 -1 0 1 0 -1 0 TimeBin*Treat*DrinkOsM 95.8 0 2.0 0 95.8 0 2.0 0 95.8 0 2.0 0 TimeBin*Treat*FrDi 1 0 -1 0 1 0 -1 0 1 0 -1 0 TimeBin*Treat*FrPoly 1 0 -1 0 1 0 -1 0 1 0 -1 0|StudyID 1 StudyID*ExptID 1 StudyID*EstimateID 1/cl divisor=3 alpha=0.1;

estimate "Hypo-iso" TimeBin*Treat 0 1 -1 0 0 1 -1 0 0 1 -1 0 TimeBin*Treat*DrinkOsM 0 -109.1 2.0 0 0 -109.1 2.0 0 0 -109.1 2.0 0 TimeBin*Treat*FrDi 0 1 -1 0 0 1 -1 0 0 1 -1 0 TimeBin*Treat*FrPoly 0 1 -1 0 0 1 -1 0 0 1 -1 0|StudyID 1 StudyID*ExptID 1 StudyID*EstimateID 1/cl divisor=3 alpha=0.1;

estimate "Water-iso" TimeBin*Treat 0 0 -1 1 0 0 -1 1 0 0 -1 1 TimeBin*Treat*DrinkOsM 0 0 2.0 0 0 0 2.0 0 0 0 2.0 0 TimeBin*Treat*FrDi 0 0 -1 1 0 0 -1 1 0 0 -1 1 TimeBin*Treat*FrPoly 0 0 -1 1 0 0 -1 1 0 0 -1 1|StudyID 1 StudyID*ExptID 1 StudyID*EstimateID 1/cl divisor=3 alpha=0.1;

estimate "Hyper-Hypo" TimeBin*Treat 1 -1 0 0 1 -1 0 0 1 -1 0 0 TimeBin*Treat*DrinkOsM 95.8 109.1 0 0 95.8 109.1 0 0 95.8 103.1 0 0 TimeBin*Treat*FrDi 1 -1 0 0 1 -1 0 0 1 -1 0 0TimeBin*Treat*FrPoly 1 -1 0 0 1 -1 0 0 1 -1 0 0|StudyID 1 StudyID*ExptID 1 StudyID*EstimateID 1/cl divisor=3 alpha=0.1; *check iso is a postivie then ave covar is a -103.1;

estimate "Hyper-Water" TimeBin*Treat 1 0 0 -1 1 0 0 -1 1 0 0 -1 TimeBin*Treat*DrinkOsM 95.8 0 0 0 95.8 0 0 0 95.8 0 0 0 TimeBin*Treat*FrDi 1 0 0 -1 1 0 0 -1 1 0 0 -1 TimeBin*Treat*FrPoly 1 0 0 -1 1 0 0 -1 1 0 0 -1|StudyID 1 StudyID*ExptID 1 StudyID*EstimateID 1/cl divisor=3 alpha=0.1;

estimate "Hypo-Water" TimeBin*Treat 0 1 0 -1 0 1 0 -1 0 1 0 -1 TimeBin*Treat*DrinkOsM 0 -109.1 0 0 0 -109.1 0 0 0 -109.1 0 0 TimeBin*Treat*FrDi 0 1 0 -1 0 1 0 -1 0 1 0 -1 TimeBin*Treat*FrPoly 0 1 0 -1 0 1 0 -1 0 1 0 -1|StudyID 1 StudyID*ExptID 1 StudyID*EstimateID 1/cl divisor=3 alpha=0.1;

*/

ods output covparms=cov;

ods output residualsdPV=residualsdPV;

ods output lsmeans=lsm;

*ods output diffs=lsmdif;

ods output estimates=est;

ods output solutionf=solf;

**run**;

ods listing;

**data** covbtwn;

set cov;

SE2=stderr****2**;

if covparm ne "Residual";

**proc** **means** noprint;

var estimate se2;

output out=covbtwn1 sum=;

**data** covbtwn2;

covparm="Btwn settings";

set covbtwn1;

StdErr=sqrt(se2);

*alpha=&alpha;

lower=estimate+probit(alpha/**2**)*StdErr;*probit not working;

upper=estimate-probit(alpha/**2**)*StdErr;

Zvalue=estimate/StdErr;

**run**;

**data** cov1;

set cov covbtwn2;

if covparm ne "Residual";

DegFree=**2***Zvalue****2**;

a=**1**;b=**1**;c=**1**;

if estimate<**0** then a=-**1**;

if lower<**0** then b=-**1**;

if upper<**0** then c=-**1**;

SD=a*sqrt(a*estimate);

lower=b*sqrt(b*lower);

upper=c*sqrt(c*upper);

array r SD lower upper;

*if &logflag=1 then do over r;

*r=100*exp(r/100)-100;

*end;

*CLtd=sqrt(upper/lower);

CLpm=(upper-lower)/**2**;

Units="Raw";

*if &logflag then Units="%";

title2 "Random effects as SD";

options ls=**100**;

**proc** **print** data=cov1 noobs;

var covparm Units SD CLpm lower upper alpha DegFree;

format SD lower upper CLpm **5.2** DegFree **5.**;

**run**;

title2 " Decisions for random effects";

**data** cov1;

set cov covbtwn2;

if covparm ne "Residual";

DF=**999**;

*MagniThresh=abs(&MagniThresh)/2;

MagniThresh=abs(**0.2*****2.62**)/**2**;*0.2 times baseline SD for dPV as a percent of mean;

*if &logflag then Magnithresh=100*exp(log(1+abs(&magnithresh)/100)/2)-100;

ProbPos=(**1**-ProbT(-(estimate-abs(MagniThresh))/StdErr,DF));

ProbNeg=ProbT(-(estimate+abs(MagniThresh))/StdErr,DF);

*if &LogFlag=1 then do;

if Magnithresh>**0** then do;

ProbPos=(**1**-ProbT(-(estimate-(MagniThresh))/StdErr,DF));

ProbNeg=ProbT(-(estimate+(MagniThresh))/StdErr,DF);

end;

else do;

ProbPos=(**1**-ProbT(-(estimate+(MagniThresh))/StdErr,DF));

ProbNeg=ProbT(-(estimate-(MagniThresh))/StdErr,DF);

end;

*end;

ProbTriv=**1**-ProbPos-ProbNeg;

ORPosNeg=ProfPos/(**1**-ProbPos)/(ProbNeg/(**1**-ProbNeg));

ORNegPos=**1**/ORPosNeg;

ClinFlag=**1**; *want all inferences to be clinical initially;

*if index(label,"2SD") then ClinFlag=0; *covariates definitely need to be non-clinical;

*mechanistic inferences as frequentist;

*ClinFlag=0;

ClearOrNot="unclear";

Strgth="";

Magni="";

ORPosNeg=**.**; ORNegPos=**.**;

if ProbNeg<**0.05** or ProbPos<**0.05** then ClearOrNot="@0.1% ";

if ProbNeg<**0.005** or ProbPos<**0.005** then ClearOrNot="@0.01% ";

if ClearOrNot ne "unclear" then do;

Magni="+ive ";

if estimate<**0** then Magni="-ive ";

if ProbPos>**0.05** or ProbNeg>**0.05** then Strgth="improbable";

if ProbPos>**0.25** or ProbNeg>**0.25** then Strgth="about as likely as not";

if ProbPos>**0.75** or ProbNeg>**0.75** then Strgth="likely ";

if ProbPos>**0.95** or ProbNeg>**0.95** then Strgth="v.likely ";

if ProbPos>**0.995** or ProbNeg>**0.995** then Strgth="virtually_certain";

end;

if ClearOrNot ne "unclear" and ProbTriv>**75** then do;

Magni="triv.";

Strgth="weak ";

if ProbTriv>**0.95** then Prob="v.likely ";

if ProbTriv>**0.995** then Prob="virtually_certain";

end;

*end;

output;

**data** cov2;

set cov1;

a=**1**;b=**1**;c=**1**;

if estimate<**0** then a=-**1**;

if lower<**0** then b=-**1**;

if upper<**0** then c=-**1**;

SD=a*sqrt(a*estimate);

lower=b*sqrt(b*lower);

upper=c*sqrt(c*upper);

DegFree=**2***Zvalue****2**;

array r SD lower upper;

/*

if &logflag=1 then do over r;

r=100*exp(r/100)-100;

end;

*/

*CLtd=sqrt(upper/lower);

CLpm=(upper-lower)/**2**;

Units="Raw";

*if &logflag then Units="%";

**run**;

options ls=**120** ps=**80**;

**proc** **print** data=cov2 noobs;

*where clinflag=0;

var covparm Units SD CLpm lower upper alpha

MagniThresh ProbNeg ProbTriv ProbPos Strgth Magni ClearOrNot;

format SD CLpm lower upper MagniThresh **5.2** Probt best5. ProbNeg ProbTriv ProbPos **5.1** DegFree **5.0**;

title3 "Non-clinical inferences for SD representing random effects";

title4 "Magnithresh is half the smallest difference for means";

**run**;

********************;

**data** est1;

set est;

*Units="Raw dPV";

CLpm=(Upper+Lower)/**2**;/*

*if &logflag then Units="factor";

array a estimate lower upper;

if &logflag then do over a;

a=exp(a/100); *all are factors;

end;

CLtd=sqrt(Upper/Lower);

if estimate=1 then do; estimate=.; Units=""; end;*/

**run**;

/*

title2 "Fixed-effect estimates";

proc print noobs data=est1;

var Label Estimate CLpm Lower Upper alpha;

format Estimate Lower Upper CLpm 5.2;

run;

*/

*magnitude-based inferences for fixed effects;

*clin and non-clin MBIs are output and listed separately;

title3 " Decisions for fixed effects";

**data** est2;

set est1;

length Strgth $ **10** Magni $ **4**;

MagniThresh=**0.2*****2.62**; *%SD baseline PV from young mean 2.03% Davy, K. P., & Seals, D. R. (1994). Total blood volume in healthy young and older men. J Appl Physiol (1985), 76(5), 2059-2062. doi: 10.1152/jappl.1994.76.5.2059

age 40-49 yr sample 3.2% from Yiengst, M. J., & Shock, N. W. (1962). Blood and plasma volume in adult males. J Appl Physiol, 17, 195-198. doi: 10.1152/jappl.1962.17.2.195;

/*

MagniThresh=0.2*2.6135; *2.6135

Hyper 2.68787

Hypo 3.07517

Iso 3.04675

Water 1.16584;

*if _n_=1 then set covarSD(keep=stddev);

*/

*SD=stddev;

Cn=**1**-**3**/(**4***df-**1**); *bias is due to uncertainty in SD;

ES=Cn*estimate/(MagniThresh/**0.2**);*SD;

*NCparm=tValue*sqrt((DFdenom+1)/(DFnum+1));

*alpha=0.1;

*CL_pc=alpha*100;

ESlower=ES/tValue*TINV(alpha/**2**,df,tvalue);

ESupper=ES/tValue*TINV(**1**-alpha/**2**,df,tvalue);

*Changed from Changes to Frequentist Probabilities by deleting 100*;

ProbPos=(**1**-ProbT(-(estimate-abs(MagniThresh))/StdErr,DF));

ProbNeg=ProbT(-(estimate+abs(MagniThresh))/StdErr,DF);

*if &LogFlag=1 then do;

if Magnithresh>**0** then do;

ProbPos=(**1**-ProbT(-(estimate-(MagniThresh))/StdErr,DF));

ProbNeg=ProbT(-(estimate+(MagniThresh))/StdErr,DF);

end;

else do;

ProbPos=(**1**-ProbT(-(estimate+(MagniThresh))/StdErr,DF));

ProbNeg=ProbT(-(estimate-(MagniThresh))/StdErr,DF);

end;

*end;

ProbTriv=**1**-ProbPos-ProbNeg;

ORPosNeg=ProfPos/(**1**-ProbPos)/(ProbNeg/(**1**-ProbNeg));

ORNegPos=**1**/ORPosNeg;

ClinFlag=**1**; *want all inferences to be clinical initially;

*if index(label,"2SD") then ClinFlag=0; *covariates definitely need to be non-clinical;

/*

*clinical inferences;

if clinflag then do;

ChPos=ProbPos; ChNeg=ProbNeg;

if MagniThresh<0 then do;

ChPos=ProbNeg; ChNeg=ProbPos;

end;

Prob=""; Magni=""; ClearOrNot="unclear";

if ChNeg<0.5 then do;

ClearOrNot="@25/.5%";

if ChNeg<0.1 then ClearOrNot="@5/.1% ";

if ProbTriv>25 then Magni="triv";

if ChPos>25 then Magni="bene";

Prob=“about as likely as not”;

if ChPos>75 or ProbTriv>75 then Prob="likely ";

if ChPos>95 or ProbTriv>95 then Prob="v.likely";

if ChPos>99.5 or ProbTriv>99.5 then Prob=“virtually certain”;

end;

else do; *i.e., ChNeg>0.5;

if ChPos<25 then do;

ClearOrNot="@25/.5%";

if ChPos<5 then ClearOrNot="@5/.1% ";

if ProbTriv>25 then Magni="triv";

if ChNeg>25 then Magni="harm";

Prob=“about as likely as not”;

if ChNeg>75 or ProbTriv>75 then Prob="likely ";

if ChNeg>95 or ProbTriv>95 then Prob="v.likely";

if ChNeg>99.5 or ProbTriv>99.5 then Prob=“virtually certain”;

end;

end;

if ClearOrNot="unclear" and

(MagniThresh>0 and ORPosNeg>25/75/(0.5/99.5) or MagniThresh<0 and ORNegPos>25/75/(0.5/99.5))

then do;

ClearOrNot="OR>66.3";

Magni="bene";

if ChPos>25 then Prob=“about as likely as not”;

if ChPos>75 then Prob="likely ";

if ChPos>95 then Prob="v.likely";

if ChPos>99.5 then Prob=“virtually certain”;

end;

output;

end;

*/

*mechanistic inferences as frequentist;

*ClinFlag=0;

ClearOrNot="unclear";

Strgth="";

Magni="";

ORPosNeg=**.**; ORNegPos=**.**;

if ProbNeg<**0.05** or ProbPos<**0.05** then ClearOrNot="@0.1% ";

if ProbNeg<**0.005** or ProbPos<**0.005** then ClearOrNot="@0.01% ";

if ClearOrNot ne "unclear" then do;

Magni="+ive ";

if estimate<**0** then Magni="-ive ";

if ProbPos>**0.05** or ProbNeg>**0.05** then Strgth="improbable";

if ProbPos>**0.25** or ProbNeg>**0.25** then Strgth="about as likely as not";

if ProbPos>**0.75** or ProbNeg>**0.75** then Strgth="likely ";

if ProbPos>**0.95** or ProbNeg>**0.95** then Strgth="v.likely ";

if ProbPos>**0.995** or ProbNeg>**0.995** then Strgth="virtually_certain";

end;

if ClearOrNot ne "unclear" and ProbTriv>**75** then do;

Magni="triv.";

Strgth="weak ";

if ProbTriv>**0.95** then Prob="v.likely ";

if ProbTriv>**0.995** then Prob="virtually_certain";

end;

*end;

output;

/*

*mechanistic inferences;

*ClinFlag=0;

ClearOrNot="unclear";

Prob="";

Magni="";

ORPosNeg=.; ORNegPos=.;

if ProbNeg<0.05 or ProbPos<0.05 then ClearOrNot="@90% ";

if ProbNeg<0.005 or ProbPos<0.005 then ClearOrNot="@99% ";

if ClearOrNot ne "unclear" then do;

Magni="+ive ";

if estimate<0 then Magni="-ive ";

if ProbPos>0.05 or ProbNeg>0.05 then Prob="unlikely";

if ProbPos>0.25 or ProbNeg>0.25 then Prob=“about as likely as not”;

if ProbPos>0.75 or ProbNeg>0.75 then Prob="likely ";

if ProbPos>0.95 or ProbNeg>0.95 then Prob="v.likely";

if ProbPos>0.995 or ProbNeg>0.995 then Prob=“virtually certain”;

end;

if ClearOrNot ne "unclear" and ProbTriv>75 then do;

Magni="triv.";

Prob="likely ";

if ProbTriv>0.95 then Prob="v.likely";

if ProbTriv>0.995 then Prob=“virtually certain”;

end;

*end;

output;

*/

/*

data est2;

Units="Raw ";

if &logflag then Units="factor";

set est1;

if estimate=0 then do;

estimate=.; magnithresh=.; magni=""; clearornot=""; Prob=""; Units="";

end;

array a estimate lower upper;

if &logflag then do over a;

a=exp(a/100); *all are factors;

end;

CLtd=sqrt(Upper/lower);

rename df=DegFree;

run;

*/

**data** est3;

set est2;

*if substr(Label,1,1)="." then delete;

*if substr(Label,1,1)="*" then Label=substr(Label,2);

*options ls=135 ps=80;

title5 "Non-clinical inferences";

title6 "Magnithresh is smallest change";

**proc** **print** data=est3 noobs;

*where clinflag=0;

var label estimate lower upper ES ESlower ESupper alpha DF

MagniThresh ProbNeg ProbTriv ProbPos Probt Strgth Magni ClearOrNot;

format estimate CLpm lower upper MagniThresh **5.1** ES ESlower ESupper **5.2** Probt best5. ProbNeg ProbTriv

ProbPos **5.4** DF **5.0**;

**run**;

**D.** **SAS code for integrating the Bayesian prior information and model into the meta-analysis data model**

*D. SAS code for integrating the Bayesian prior information and model into the meta-analysis data model

*credit for equations used in code W.G. Hopkins Bayesian spreadsheet <https://www.sportsci.org/2019/bayes.htm>. Drawn from Greenland S. Bayesian perspectives for epidemiological research: I. Foundations and basic methods. International Journal of Epidemiology. 2006;35:765-75.;

*takes usual output from an analysis of estimates (i.e. dataset est3 above) and adds priors;

*incoming data have these variables:

Label Modifier Treatment Timebin Estimate Lower Upper ES ESlower ESupper

Alpha DF MagniThresh ProbNeg ProbTriv ProbPos Probt Strgth Magni;

FILENAME REFFILE 'C:\Users\dsrowlan\OneDrive - Massey University\Documents\Massey\Massey_2021\Research\Frucor_Hydration Review\

ECSS_2018_Manuscript meta-analysis\New_Dec 2019\Working SAS_Feb 2020\Bayesian July 2021\

Meta effects for Bayes.xlsx';

**PROC** **IMPORT** DATAFILE=REFFILE

DBMS=XLSX

OUT=est replace;

GETNAMES=YES;

**RUN**;

%let logflag=0;*code to tell SAS to select given code block. if 0, then nothing is 100*log transformed, if =1 then data are 100*log transformed;

%let deceff=1;*decimal places for any effects;

%let MeanPrior=0;

%let CLpmPrior=(2.5/0.3*0.75); *in standardized units and previously =2 when using 2SD as the error;*2.5/0.3*0.75, where 0.3 comes from smallest effect of CV for performance and PV assocation;

%let DFprior=999;

**data** _null_;

if &logflag then call symput('unitsrawlog',"percent");

else call symput('unitsrawlog',"raw");

**data** thresholds;

set est(keep=MagniThresh);

*drop ProbNeg ProbTriv ProbPos;

if _n_=**1**;

call symput('magnithresh',Magnithresh); *makes a macro variable available for the nest title;

Units="Raw"; *not used;

if &logflag then Units="%";

if &logflag=**0** then do; *these raw thresholds are actually via standardization;

Threshold="small "; DeltaMeanBene=magnithresh; DeltaMeanHarm=-magnithresh; SDthreshold=abs(magnithresh)/**2**; output;

Threshold="moderate"; DeltaMeanBene=magnithresh*(**0.9**/**.3**); DeltaMeanHarm=-magnithresh*(**0.9**/**.3**); SDthreshold=abs(DeltaMeanBene)/**2**; output;

Threshold="large"; DeltaMeanBene=magnithresh*(**1.6**/**.3**); DeltaMeanHarm=-magnithresh*(**1.6**/**.3**); SDthreshold=abs(DeltaMeanBene)/**2**; output;

Threshold="vlarge"; DeltaMeanBene=magnithresh*(**2.5**/**.3**); DeltaMeanHarm=-magnithresh*(**2.5**/**.3**); SDthreshold=abs(DeltaMeanBene)/**2**; output;

Threshold="xlarge"; DeltaMeanBene=magnithresh*(**4.0**/**.3**); DeltaMeanHarm=-magnithresh*(**4.0**/**.3**); SDthreshold=abs(DeltaMeanBene)/**2**; output;

end;

/*

Threshold="small "; DeltaMeanBene=magnithresh; DeltaMeanHarm=-magnithresh; SDthreshold=abs(magnithresh)/2; output;

Threshold="moderate"; DeltaMeanBene=magnithresh*3; DeltaMeanHarm=-magnithresh*3; SDthreshold=abs(DeltaMeanBene)/2; output;

Threshold="large"; DeltaMeanBene=magnithresh*6; DeltaMeanHarm=-magnithresh*6; SDthreshold=abs(DeltaMeanBene)/2; output;

Threshold="vlarge"; DeltaMeanBene=magnithresh*10; DeltaMeanHarm=-magnithresh*10; SDthreshold=abs(DeltaMeanBene)/2; output;

Threshold="xlarge"; DeltaMeanBene=magnithresh*20; DeltaMeanHarm=-magnithresh*20; SDthreshold=abs(DeltaMeanBene)/2; output;

end;*/

else do; *these are for percent effects on athletic performance;

magnithresh=**100***log(**1**+magnithresh/**100**);

Threshold="small "; DeltaMeanBene=**100***exp(magnithresh/**100**)-**100**; DeltaMeanHarm=**100***exp(-magnithresh/**100**)-**100**; SDthreshold=**100***exp(abs(magnithresh)/**100**/**2**)-**100**; output;

Threshold="moderate"; DeltaMeanBene=**100***exp(magnithresh/**100*****3**)-**100**; DeltaMeanHarm=**100***exp(-magnithresh/**100*****3**)-**100**; SDthreshold=**100***exp(abs(magnithresh)/**100**/**2*****3**)-**100**; output;

Threshold="large"; DeltaMeanBene=**100***exp(magnithresh/**100*****1.6**/**0.3**)-**100**; DeltaMeanHarm=**100***exp(-magnithresh/**100*****1.6**/**0.3**)-**100**; SDthreshold=**100***exp(abs(magnithresh)/**100**/**2*****1.6**/**0.3**)-**100**; output;

Threshold="vlarge"; DeltaMeanBene=**100***exp(magnithresh/**100*****2.5**/**0.3**)-**100**; DeltaMeanHarm=**100***exp(-magnithresh/**100*****2.5**/**0.3**)-**100**; SDthreshold=**100***exp(abs(magnithresh)/**100**/**2*****2.5**/**0.3**)-**100**; output;

Threshold="xlarge"; DeltaMeanBene=**100***exp(magnithresh/**100*****4.0**/**0.3**)-**100**; DeltaMeanHarm=**100***exp(-magnithresh/**100*****4.0**/**0.3**)-**100**; SDthreshold=**100***exp(abs(magnithresh)/**100**/**2*****4.0**/**0.3**)-**100**; output;

end;

**run**;

title1 "Magnitude thresholds (&unitsrawlog), based on a given smallest important for means of &magnithresh (&unitsrawlog).";

*title2 "Thresholds for moderate, large, v.large and x.large are 3, 6, 10, and 20 times the smallest important,";

*title3 "i.e., standardization thresholds; *thresholds for SDs are half those for means.";

**proc** **print** noobs;

var Threshold Units DeltaMeanBene DeltaMeanHarm SDthreshold;

format DeltaMeanBene DeltaMeanHarm SDthreshold **5.**&deceff;

where &logflag=**0**;

**run**;

/*

proc print noobs;

var Threshold Units DeltaMeanBene DeltaMeanHarm SDthreshold;

format DeltaMeanBene DeltaMeanHarm 5.&deceff SDthreshold 5.2;

where &logflag=1;

run;

*/

*make macro variables for magnitude thresholds for the MBD steps;

**data** _null_;

length ThreshX $ **9**;

set thresholds;

if &logflag then Thresh=abs(**100***log(**1**+DeltaMeanBene/**100**)); *convert back to log value, could do it with DeltaMeanHarm;

else Thresh=abs(DeltaMeanBene);

ThreshX=trim(Threshold)||'X';* ;

call symput(ThreshX,Thresh);*a handy piece of code to generate a macro variable;

**run**;

title2 "MBD for differences in least-squares means";

**data** est1;

set est(rename=(Estimate=EstSample Lower=LowSamp Upper=UppSamp DF=DFsamp Strgth=ProbSamp Magni=MagniSamp));

*Davids code to re-rerun unadjusted previously 2SD for SIE with new SIE (0.75%);

StdErr=(UppSamp-LowSamp)/**2**/tinv(**0.95**,DFsamp);

ProbPosSamp=(**1**-ProbT(-(EstSample-abs(magnithresh))/StdErr,DFsamp));

ProbNegSamp=ProbT(-(EstSample+abs(magnithresh))/StdErr,DFsamp);

*if &LogFlag=1 then do;

if magnithresh>**0** then do;

ProbPosSamp=(**1**-ProbT(-(EstSample-(magnithresh))/StdErr,DFsamp));

ProbNegSamp=ProbT(-(EstSample+(magnithresh))/StdErr,DFsamp);

end;

else do;

ProbPosSamp=(**1**-ProbT(-(EstSample+(magnithresh))/StdErr,DFsamp));

ProbNegSamp=ProbT(-(EstSample-(magnithresh))/StdErr,DFsamp);

end;

*end;

ProbTrivSamp=**1**-ProbPosSamp-ProbNegSamp;

ORPosNegSamp=ProfPosSamp/(**1**-ProbPosSamp)/(ProbNegSamp/(**1**-ProbNegSamp));

ORNegPosSamp=**1**/ORPosNegSamp;

*ClinFlag=1;

ClearOrNot="unclear";

ProbSamp="";

MagniSamp="";

ORPosNegSamp=**.**; ORNegPosSamp=**.**;

if ProbNegSamp<**0.05** or ProbPosSamp<**0.05** then ClearOrNot="@0.1% ";

if ProbNegSamp<**0.005** or ProbPosSamp<**0.005** then ClearOrNot="@0.01% ";

if ClearOrNot ne "unclear" then do;

MagniSamp="+ive ";

if EstSample<**0** then MagniSamp="-ive ";

if ProbPosSamp>**0.05** or ProbNegSamp>**0.05** then ProbSamp="improbable";

if ProbPosSamp>**0.25** or ProbNegSamp>**0.25** then ProbSamp="possibly";

if ProbPosSamp>**0.75** or ProbNegSamp>**0.75** then ProbSamp="likely ";

if ProbPosSamp>**0.95** or ProbNegSamp>**0.95** then ProbSamp="v.likely ";

if ProbPosSamp>**0.995** or ProbNegSamp>**0.995** then ProbSamp="m.likely";

end;

if ClearOrNot ne "unclear" and ProbTrivSamp>**75** then do;

MagniSamp="triv.";

ProbSamp="likely ";

if ProbTrivSamp>**0.95** then Prob="v.likely ";

if ProbTrivSamp>**0.995** then Prob="m.likely";

end;

*end;

output;

*code continues;

CLpmSamp=(UppSamp-LowSamp)/**2**;

SEsamp=CLpmSamp/tinv(**1**-alpha/**2**,DFsamp);

SEprior=&CLpmPrior/tinv(**1**-alpha/**2**,&DFprior);*for when CLPrior is in actual units;

Estimate=**1**/(**1**/SEprior****2**+**1**/SEsamp****2**)*(**1**/SEprior****2***&MeanPrior+**1**/SEsamp****2***EstSample);

StdErr=sqrt((**1**/(**1**/SEprior****2**+**1**/SEsamp****2**)*(**1**/SEprior****2***SEprior))****2**+(**1**/(**1**/SEprior****2**+**1**/SEsamp****2**)*(**1**/SEsamp****2***SEsamp))****2**);

DFnumer=((**1**/(**1**/SEprior****2**+**1**/SEsamp****2**)*(**1**/SEprior****2***SEprior))****2**+(**1**/(**1**/SEprior****2**+**1**/SEsamp****2**)*(**1**/SEsamp****2***SEsamp))****2**)****2**;

DFdenom=(**1**/(**1**/SEprior****2**+**1**/SEsamp****2**)*(**1**/SEprior****2***SEprior))****4**/&DFprior+(**1**/(**1**/SEprior****2**+**1**/SEsamp****2**)*(**1**/SEsamp****2***SEsamp))****4**/DFsamp;

DF=DFnumer/DFdenom;

drop DFnumer DFdenom;

Units="%"; *not used;

length Prob $ **8** Magni $ **9** QualMag $ **8**;

QualMag="Trivial";

if abs(estimate)>&smallx then QualMag="Small";*start of code to get the threshold from the macro under data _null_;

if abs(estimate)>&moderatex then QualMag="Moderate";

if abs(estimate)>&largex then QualMag="Large";

if abs(estimate)>&vlargex then QualMag="Vlarge";

if abs(estimate)>&xlargex then QualMag="Xlarge";

ChancePos=**100***(**1**-ProbT(-(estimate-abs(MagniThresh))/StdErr,DF));

ChanceNeg=**100***ProbT(-(estimate+abs(MagniThresh))/StdErr,DF);

if &LogFlag=**1** then do;

if Magnithresh>**0** then do; *assumes smallest is in percent units;

ChancePos=**100***(**1**-ProbT(-(estimate-**100***log(**1**+MagniThresh/**100**))/StdErr,DF));

ChanceNeg=**100***ProbT(-(estimate+**100***log(**1**+MagniThresh/**100**))/StdErr,DF);

end;

else do;

ChancePos=**100***(**1**-ProbT(-(estimate+**100***log(**1**+MagniThresh/**100**))/StdErr,DF));

ChanceNeg=**100***ProbT(-(estimate-**100***log(**1**+MagniThresh/**100**))/StdErr,DF);

end;

end;

ChanceTriv=**100**-ChancePos-ChanceNeg;

ORPosNeg=ChancePos/(**100**-ChancePos)/(ChanceNeg/(**100**-ChanceNeg));

ORNegPos=**1**/ORPosNeg;

ClinFlag=**1**; *want all inferences to be clinical initially;

*if index(label,"2SD") then ClinFlag=0; *covariates definitely need to be non-clinical;

*clinical inferences;

if clinflag then do;

ChPos=ChancePos; ChNeg=ChanceNeg;

if MagniThresh<**0** then do;

ChPos=ChanceNeg; ChNeg=ChancePos;

end;

Prob=""; Magni=""; Precision="unclear";

if ChNeg<**0.5** then do;

Precision="@25/.5%";

if ChNeg<**0.1** then Precision="@5/.1% ";

if ChanceTriv>**25** then Magni="triv";

if ChPos>**25** then Magni="bene";

Prob="possibly";

if ChPos>**75** or ChanceTriv>**75** then Prob="likely ";

if ChPos>**95** or ChanceTriv>**95** then Prob="v.likely";

if ChPos>**99.5** or ChanceTriv>**99.5** then Prob="m.likely";

end;

else do; *i.e., ChNeg>0.5;

if ChPos<**25** then do;

Precision="@25/.5%";

if ChPos<**5** then Precision="@5/.1% ";

if ChanceTriv>**25** then Magni="triv";

if ChNeg>**25** then Magni="harm";

Prob="possibly";

if ChNeg>**75** or ChanceTriv>**75** then Prob="likely ";

if ChNeg>**95** or ChanceTriv>**95** then Prob="v.likely";

if ChNeg>**99.5** or ChanceTriv>**99.5** then Prob="m.likely";

end;

end;

if Precision="unclear" and

(MagniThresh>**0** and ORPosNeg>**25**/**75**/(**0.5**/**99.5**) or MagniThresh<**0** and ORNegPos>**25**/**75**/(**0.5**/**99.5**))

then do;

Precision="OR>66.3";

Magni="bene";

if ChPos>**25** then Prob="possibly";

if ChPos>**75** then Prob="likely ";

if ChPos>**95** then Prob="v.likely";

if ChPos>**99.5** then Prob="m.likely";

end;

output;

end;

*mechanistic inferences;

ClinFlag=**0**;

Precision="unclear";

Prob="";

Magni="";

ORPosNeg=**.**; ORNegPos=**.**;

if ChanceNeg<**5** or ChancePos<**5** then Precision="@90% ";

if ChanceNeg<**0.5** or ChancePos<**0.5** then Precision="@99% ";

if Qualmag ne "Trivial" then do;

Magni="+ive ";

if estimate<**0** then Magni="-ive ";

end;

if Precision ne "unclear" then do;

if ChancePos>**5** or ChanceNeg>**5** then Prob="unlikely";

if ChancePos>**25** or ChanceNeg>**25** then Prob="possibly";

if ChancePos>**75** or ChanceNeg>**75** then Prob="likely ";

if ChancePos>**95** or ChanceNeg>**95** then Prob="v.likely";

if ChancePos>**99.5** or ChanceNeg>**99.5** then Prob="m.likely";

end;

if Precision ne "unclear" and ChanceTriv>**75** then do;

Magni="triv.";

Prob="likely ";

if ChanceTriv>**95** then Prob="v.likely";

if ChanceTriv>**99.5** then Prob="m.likely";

end;

if **25**<ChancePos<**75** then xtra="/+ive";

if **25**<ChanceNeg<**75** then xtra="/-ive";

if Precision ne "unclear" and QualMag="Trivial" then Magni="triv"||xtra;

if Precision ne "unclear" and QualMag ne "Trivial" and prob="possibly" then do;

if **25**<ChanceTriv<**75** then Magni=trim(Magni)||"/triv";

end;

*end;

output;

**data** est2;

*Units="Raw";

*if &logflag then Units="%";

set est1;

if estimate=**0** or estimate=**.** then do;

estimate=**.**; magnithresh=**.**; magni=""; Precision=""; Prob=""; Units=""; QualMag="";

end;

Lower=estimate-tinv(**1**-alpha/**2**,DF)*Stderr;

Upper=estimate+tinv(**1**-alpha/**2**,DF)*Stderr;

array a estimate lower upper;

if &logflag then do over a;

a=**100***exp(a/**100**)-**100**; *all are factors;

end;

CLpm=(Upper-Lower)/**2**;

ProbNegBayes=ChanceNeg/**100**;

ProbTrivBayes=ChanceTriv/**100**;

ProbPosBayes=ChancePos/**100**;

rename df=DegFree;

if ProbSamp="possible" then ProbSamp="possibly";

if ProbSamp="xlikely" then ProbSamp="m.likely";

if transtrn(prob,".",trimn('')) ne ProbSamp then Shrinkage="yes";

**run**;

title1 "Non-clinical inferences, original sample and Bayesian modified";

title2 "Prior mean = &MeanPrior; prior 90%CLpm = &CLpmPrior in standardized units";

title3 "Magnithresh is smallest mean change";

title4 "Bayesian estimates start at the Estimate column";

**proc** **print** data=est2 noobs;

var Label Modifier Treatment Timebin

Alpha MagniThresh EstSample LowSamp UppSamp CLpmSamp DFsamp ProbNegSamp ProbTrivSamp ProbPosSamp Probt ProbSamp MagniSamp

Estimate lower upper CLpm DegFree

ProbNegBayes ProbTrivBayes ProbPosBayes QualMag Prob Magni Precision Shrinkage;

format estimate CLpm lower upper MagniThresh **5.**&deceff ChanceNeg ChanceTriv

ChancePos **5.1** DegFree **5.0**;

where clinflag=**0**;

**run**;

/*

*options ls=145 ps=80;

title3 "Clinical inferences";

title4 "Magnithresh is smallest beneficial change";

proc print data=est4 noobs;

where clinflag=1;

var label estimate CLpm lower upper alpha DegFree

MagniThresh ChanceNeg ChanceTriv ChancePos ORPosNeg ORNegPos Prob Magni Precision;

format estimate CLpm lower upper MagniThresh 5.&decdep Probt best5. ORPosNeg ORNegPos 5.0 ChanceNeg ChanceTriv

ChancePos 5.1 DegFree 5.0;

run;

*/

**Declarations:** No external funding was provided for the completion of this meta-analysis and review. David Rowlands received funding from Frucor Ltd (NZ), and Lucozade-Ribena-Suntory (UK) to previously report upon hypotonic drink effects on hydration. Brigitte Kopetschny and Claire Badenhorst declare that they have no potential conflicts of interest relevant to the content of this review.
